# NMR-filtered virtual screening leads to non-metal chelating metallo-β-lactamase inhibitors†
†Electronic supplementary information (ESI) available: All experimental details, crystallographic data collection and refinement statistics, details of chemical synthesis, additional figures and tables. See DOI: 10.1039/c6sc04524c



**DOI:** 10.1039/c6sc04524c

**Published:** 2016-12-14

**Authors:** Guo-Bo Li, Martine I. Abboud, Jürgen Brem, Hidenori Someya, Christopher T. Lohans, Sheng-Yong Yang, James Spencer, David W. Wareham, Michael A. McDonough, Christopher J. Schofield

**Affiliations:** a Department of Chemistry , University of Oxford , 12 Mansfield Road , Oxford , OX1 3TA , UK . Email: christopher.schofield@chem.ox.ac.uk ; Email: michael.mcdonough@chem.ox.ac.uk; b Key Laboratory of Drug Targeting and Drug Delivery System of Ministry of Education , West China School of Pharmacy , Sichuan University , Chengdu , 610041 , China; c Medicinal Chemistry Research Laboratories , New Drug Research Division , Otsuka Pharmaceutical Co., Ltd. , 463-10 Kagasuno, Kawauchi-cho , Tokushima 771-0192 , Japan; d State Key Laboratory of Biotherapy/Collaborative Innovation Center for Biotherapy , West China Hospital , West China Medical School , Sichuan University , Sichuan 610041 , China; e School of Cellular and Molecular Medicine , Biomedical Sciences Building , University of Bristol , Bristol BS8 1TD , UK; f Antimicrobial Research Group , Barts & The London School of Medicine and Dentistry , Queen Mary University of London , London , E1 2AT , UK

## Abstract

There are no clinically useful inhibitors of metallo-β-lactamases (MBLs), which are a growing problem because they hydrolyse almost all β-lactam antibacterials. Inhibition by most reported MBL inhibitors involves zinc ion chelation. A structure-based virtual screening approach combined with NMR filtering led to the identification of inhibitors of the clinically relevant Verona Integron-encoded MBL (VIM)-2. Crystallographic analyses reveal a new mode of MBL inhibition involving binding adjacent to the active site zinc ions, but which does not involve metal chelation. The results will aid efforts to develop new types of clinically useful inhibitors targeting MBLs/MBL-fold metallo-enzymes involved in antibacterial and anticancer drug resistance.

## Abstract 2

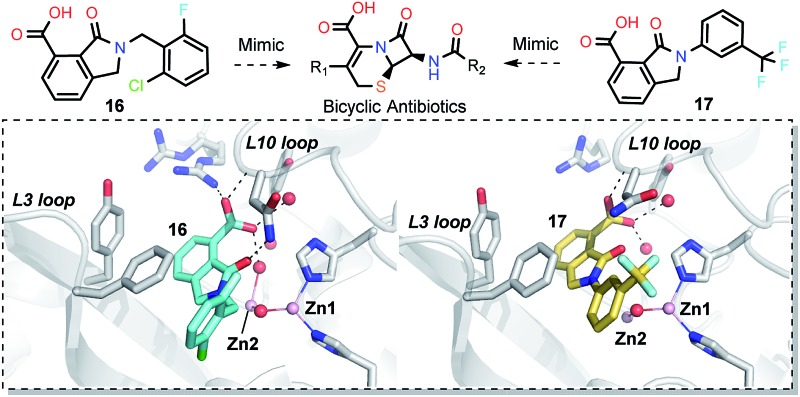
NMR-filtered virtual screening led to the identification of non-Zn(ii)-chelating metallo-β-lactamase inhibitors, which mimic interactions made by the bicyclic β-lactam antibiotic substrates as they initially bind to the enzymes.

## Introduction

**Fig. 1:**
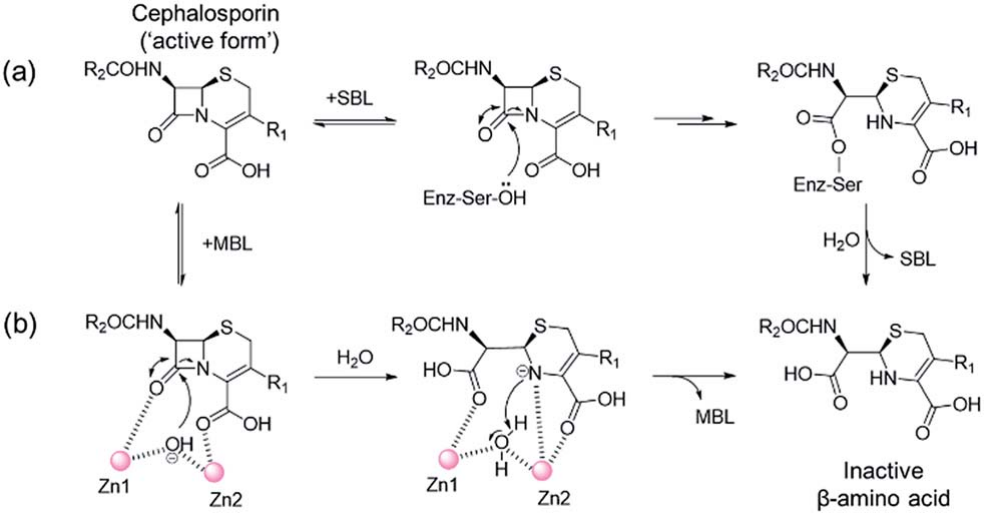
Outline mechanisms for (a) serine- (SBL) and (b) metallo-β-lactamase (MBL) catalysed hydrolysis. Note, in the case of the MBL variations of this mechanism are possible.

One of the most important mechanisms of resistance to β-lactam antibacterials involves their hydrolysis as catalysed by serine-β-lactamases (SBLs) and metallo-β-lactamases (MBLs) (Fig. 1).^1,2^ Although clinically useful inhibitors of the SBLs are established, there are no such MBL inhibitors.^3–7^ Inhibitors of human MBL-fold proteins are also of medicinal interest, including for the human DNA cross-link repair enzymes SNM1-A/B, in order to combat resistance to major anti-cancer drugs such as cisplatin.^8,9^ To date, almost all reported MBL inhibitors work *via* zinc ion chelation (Fig. S1 and S2†).^10–13^ Development of new types of MBL-fold enzyme inhibitors that do not work *via* metal chelation is presently desirable, in part because this may enable improved selectivity than (readily) achievable with zinc ion chelation. Here we report how a virtual screening approach combined with NMR filtering, led to the identification of non-metal chelating inhibitors of the clinically relevant Verona Integron-encoded MBL (VIM)-2. As revealed by crystallographic, NMR, and biochemical studies, the new inhibitors bind *via* a mode that does not involve direct zinc chelation, but which may mimic interactions made by intact β-lactam substrates as they initially bind to VIM-2. VIM-2 is a clinically important representative of the class B1 MBLs (which also includes the imipenemase (IMP)-1, and New Delhi MBL (NDM)-1), that have a broad-spectrum substrate profile that includes penicillins, cephamycins, cephalosporins, oxacephamycins, and carbapenems.^14^ The B1 subfamily MBLs are di-Zn(ii) utilizing enzymes, with both the Zn1 and Zn2 ions having crucial roles in catalysis, with respect to β-lactam substrate binding, and hydrolytic water activation.^15–17^

We began by carrying out a virtual screen with VIM-2 for which several high-resolution (<1.5 Å) crystal structures are available.^12,18–20^ A customized virtual screening method, which combines molecular docking simulations with a molecular interaction fingerprints (IFPs)-based filtering approach, was used to identify compounds that are likely to interact with catalytically important active site residues including Arg228, Asn233, Phe61, Tyr67, and Asp120 (using the standard BBL numbering scheme for class B β-lactamases^21^) as well as zinc ions. Eight types of protein-ligand interactions (hydrogen-bonding donor, hydrogen-bonding acceptor, positively charged, negatively charged, face-to-face π–π stacking, edge-to-face π–π stacking, hydrophobic, and metal–ligand interactions) as defined in our previous work^22,23^ were used to generate the IFPs (for details of the virtual screening methods see ESI Experimental section SE. 1 and Fig. S3†). Although our strategy included the identification of potential zinc ion binding inhibitors, since we have found experimentally that metal ion chelation can serve to ‘template’ ligands to the active sites of metallo-enzymes,^24–26^ we were particularly interested in the identification of non-Zn chelating inhibitors. We subsequently screened selected compounds identified in the virtual screen for activity against VIM-2 using a fluorescence-based assay.^27^ We then used ligand-observe ^1^H Carr–Purcell–Meiboom–Gill (CPMG) NMR spectroscopy^28^ to test for binding to both the apo-VIM-2 and catalytically active di-Zn(ii) VIM-2, with the aim of establishing whether the zinc ions are required for inhibitor binding.

## Results

Application of the virtual screening method led to the identification of a number of fragment-sized compounds, mostly containing acidic groups, that are likely to interact with catalytically important active site features, replicating interactions involved in binding the carboxylate present in β-lactam antibacterials (*i.e.* with Arg228, Fig. S4–S6†). Of the 20 experimentally tested compounds, 15 exhibited inhibitory activity against VIM-2, and 8 compounds manifested IC_50_ values <400 μM, including compounds **6**, **7**, **12**, **13**, **16**, **17**, **18**, and **20** (Table 1 and Fig. S7†). Using the same assay conditions, we observed that l-captopril inhibits VIM-2 with an IC_50_ value of 1.6 μM. As negative controls, we tested four compounds which did not pass the IFP score cut-off in the virtual screen at 2 mM against VIM-2 (**44–47**, Fig. S6, Table S4†); these compounds displayed substantially weaker inhibition than those with IFP scores above the cut-off.

**Table 1:** Chemical structures and inhibitory potencies against VIM-2 of compounds identified by virtual screening

Cpd ID^*a*^	Chemical structure	IC_50_ (μM)/pIC_50_/s.e. log IC_50_ ^*b*^
**1**	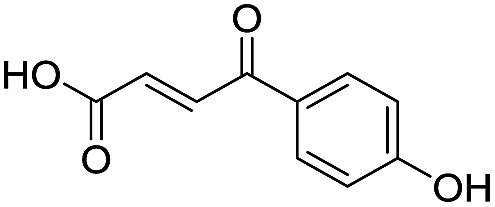	637/3.20/0.063
**2**	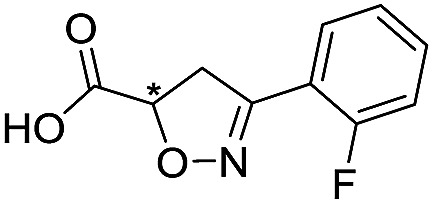	993/3.00/0.064
**3**	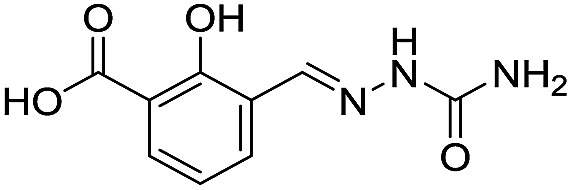	>1600/<2.8/—
**4**	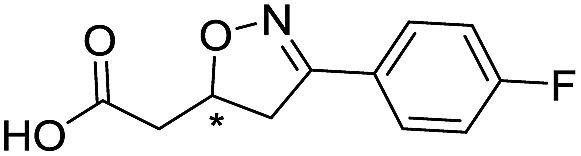	557/3.25/0.135
**5**	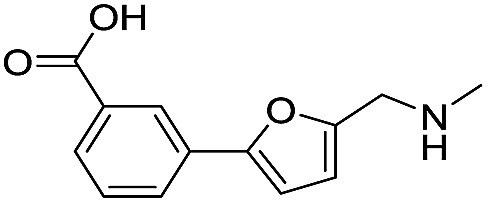	>1600/<2.8/—
**6**	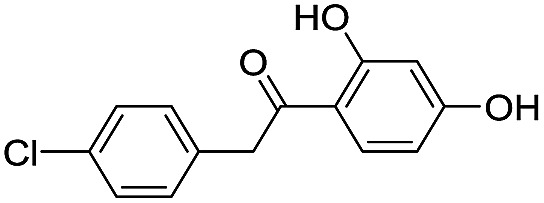	111/3.95/0.121
**7**	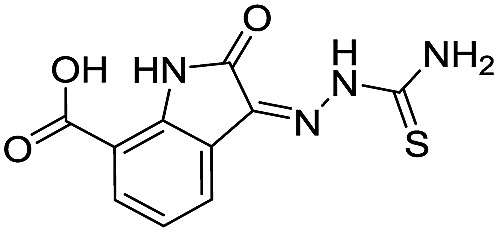	199/3.70/0.060
**8**	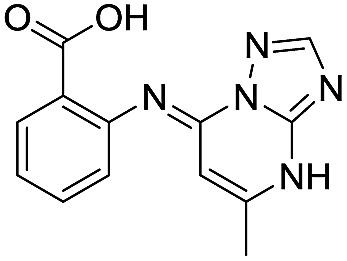	>1600/<2.8/—
**9**	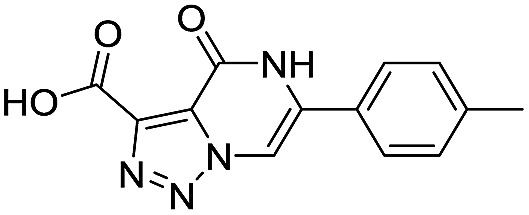	439/3.36/0.072
**10**	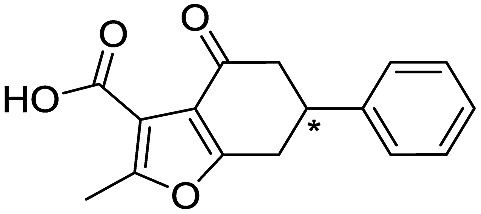	>1600/<2.8/—
**11**	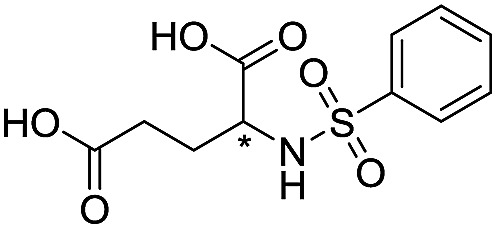	1110/2.96/0.043
**12**	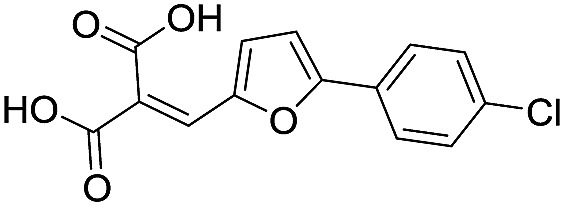	87/4.06/0.167
**13**	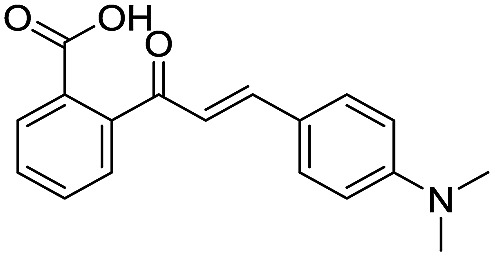	135/3.87/0.111
**14**	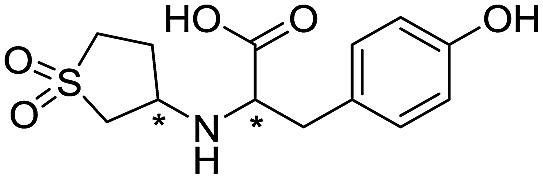	669/3.18/0.107
**15**	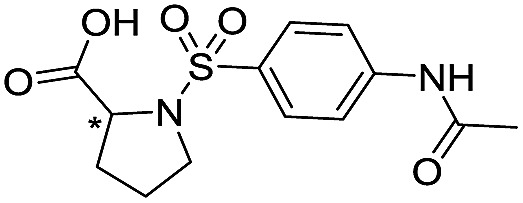	>1600/<2.8/—
**16**	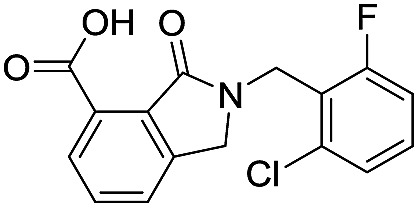	10.6/4.98/0.119
**17**	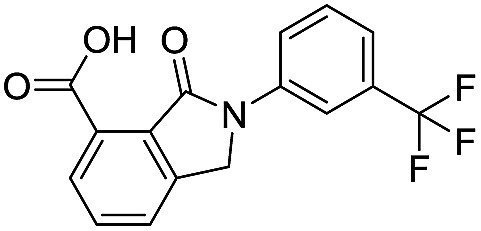	32.8/4.48/0.055
**18**	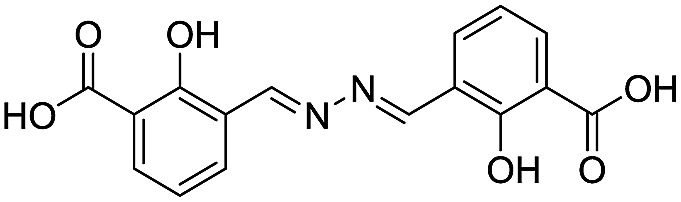	117/3.93/0.104
**19**	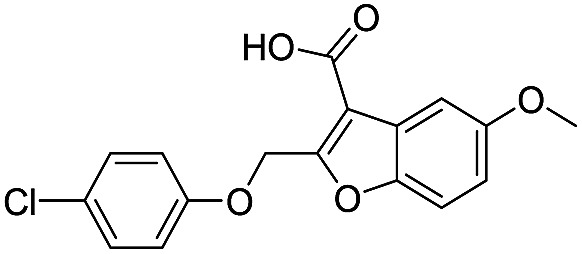	718/3.14/0.212
**20**	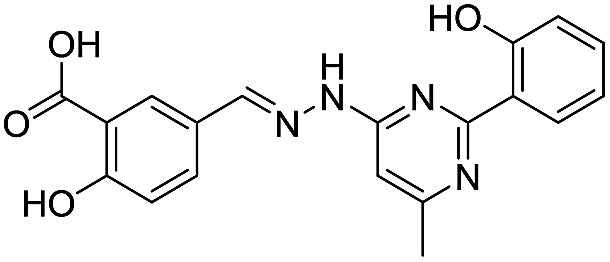	292/2.54/2.26
l-Captopril	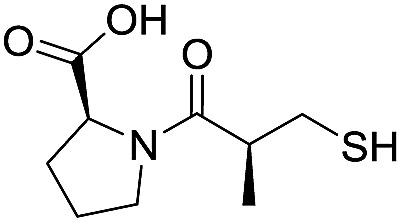	1.6/5.80/0.02

^*a*^Compounds **2**, **4**, **10**, **11**, **14**, and **15** were tested as stereomeric mixtures.

^*b*^The method for measuring IC_50_/pIC_50_ (*n* ≥ 3) values is described in ESI methods;^27^ IC_50_ curves are given in Fig. S7.

Since some of the compounds, *e.g.* **6**, **7**, **12**, **13**, **16**, **17**, **18**, and **20**, possess potential metal-chelating motifs, further VIM-2 inhibition assays at three different concentrations of Zn(ii) (0 μM, 1 μM, and 100 μM) were employed to investigate the potential for metal chelation in solution. With the exception of compound **18**, no obvious differences between the inhibitory activity with or without excess Zn(ii) were observed, suggesting that most of these compounds were not strong Zn(ii) chelators in solution (Fig. S8†). l-Captopril was used as a positive control for active site/metal binding^18^ and showed IC_50_ values of 1.9 μM, 1.6 μM, and 1.4 μM at concentrations of 0 μM, 1 μM, and 100 μM Zn(ii), respectively. Compound **18**, however, likely causes inhibition, at least in part, by chelating Zn(ii) in solution, so sequestering it from the enzyme.

**Fig. 2:**
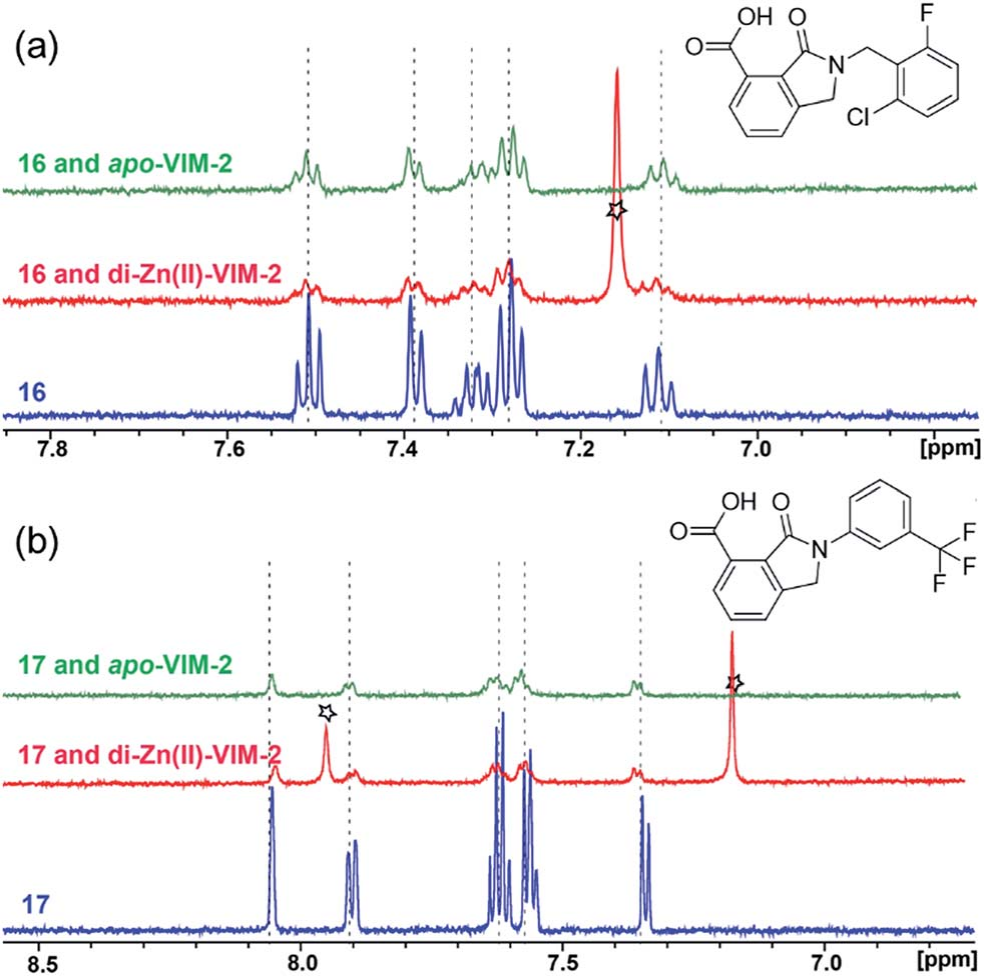
^1^H CPMG NMR analyses reveal that compounds **16** and **17** bind to both di-Zn(ii) and apo-VIM-2 MBL. Binding studies of **16** (a) and **17** (b) to di-Zn(ii)-VIM-2 and apo-VIM-2 by ^1^H CPMG NMR analyses. **16** and **17** bind to both di-Zn(ii) and apo-VIM-2 as indicated by signal intensity reduction in the presence of VIM-2. Assay mixtures contained 50 μM enzyme (either di-Zn(ii) VIM-2 in the presence of 50 μM Zn(ii) or apo-VIM-2), and 50 μM of the compound of interest buffered with 50 mM Tris-D_11_, pH 7.5, in 90% H_2_O and 10% D_2_O. Black stars denote imidazole in the di-Zn(ii)-VIM-2 buffer (Fig. S15†). Note, the ^1^H NMR spectra of **16** and **17** in DMSO-D_6_ (Fig. S16†) are different from those in 50 mM Tris-D_11_, pH 7.5, in 90% H_2_O and 10% D_2_O.

With the aim of identifying compounds that bind (at least, substantially) without active site Zn(ii) chelation, we used ^1^H CPMG NMR (ESI Experimental section SE. 4†) to investigate binding to catalytically active di-Zn(ii) VIM-2 as well as to the apo-VIM-2 form for selected compounds from the virtual screen (including **6**, **7**, **12**, **13**, **16**, **17**, and **18**). Notably, for **16** and **17**, which displayed the most potent VIM-2 inhibition (IC_50_ values of 10.6 μM and 32.8 μM, respectively, Table 1), we observed binding to both the di-Zn(ii) VIM-2 and apo-VIM-2 protein by ^1^H CPMG NMR analyses (Fig. 2a and b). For comparison, we selected a known inhibitor of the VIM-2 MBL, l-captopril, which works *via* an established binding mode that involves direct zinc chelation as revealed by crystallographic analyses;^18,29^ consistent with its reported mode of binding, we observed that l-captopril displays strong binding to di-Zn(ii)-VIM-2, but only very weak binding to apo-VIM-2 (Fig. S9†). The reduction in signal intensity observations with **16** and **17** suggest that they bind more tightly to di-Zn(ii) than apo-VIM-2 (Fig. 2), possibly reflecting the more ordered structure of the di-Zn(ii) enzyme.

Overall, these results show that **16** or **17** can bind to VIM-2 in the absence of zinc ions. However, the presence of zinc indirectly participates in the binding of these inhibitors to VIM-2, possibly through protein stabilisation, bridging interactions or a combination thereof. We also observed that **6** (IC_50_ = 111 μM) displays strong binding to both di-Zn(ii) VIM-2 and apo-VIM-2 (Fig. S10†), indicating that, like **16** and **17**, **6** may bind without metal chelation. In contrast, **12** (IC_50_ = 87 μM) and **13** (IC_50_ = 135 μM) displayed strong binding to di-Zn(ii) VIM-2, but weak binding to the apo-VIM-2 protein (Fig. S11 and S12†), suggesting that these compounds inhibit VIM-2 by a mode directly involving zinc chelation. For **7** and **18**, for which assays showed VIM-2 inhibition (IC_50_ values of 199 μM and 117 μM, respectively, Table 1), we observed binding to both di-Zn(ii)- and apo-VIM-2 (Fig. S13 and S14†) by NMR. In contrast to **16** and **17** (see below), **12** and **13**, which may be metal-chelating VIM-2 inhibitors as observed in the inhibition assays and NMR studies (Table 1, Fig. S11 and S12†), displayed broad-spectrum inhibition against almost all the tested class B MBLs (Fig. S21†).

**Fig. 3:**
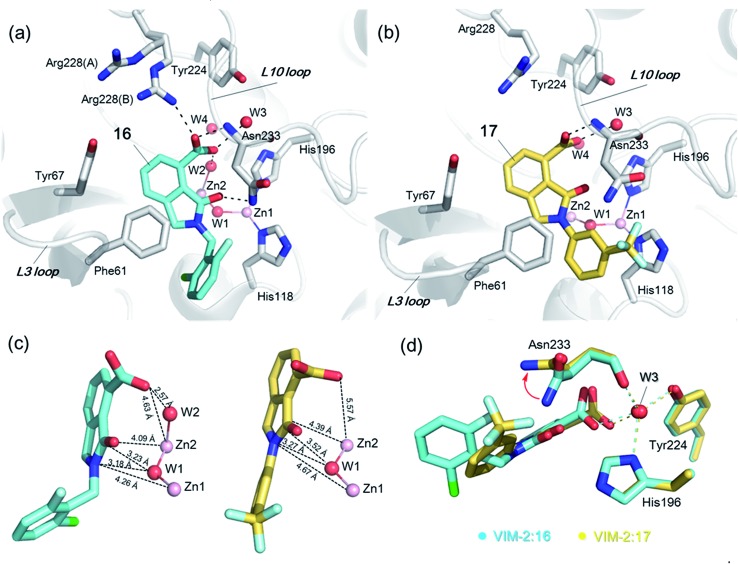
Crystallographic analyses reveals compound **16** and **17** binding modes to VIM-2. (a) View from a crystal structure of VIM-2 in complex with compound **16** (PDB ID ; 5LE1) reveals that the inhibitor binds to form hydrophobic and electrostatic interactions with residues on the L3 and L10 loops (*e.g.*, π–π stacking interactions with Phe61, and hydrogen-bonding interactions with Asn233; see Fig. S19† for further details of binding interaction). (b) View from a crystal structure of VIM-2 in complex with **17** (PDB ID ; 5LCA) reveals that **17** binds *via* a similar mode with that of **16**. (c) Comparison of complex structures of VIM-2:**16** and VIM-2:**17** shows that both bind adjacent to zinc ions of VIM-2, but in a mode which does not involve direct zinc chelation. Distances between the oxygen atoms of the carboxylate of **16** and **17** and Zn2 are 4.63 Å and 5.57 Å, respectively. (d) The water molecule W3 is positioned to form tight hydrogen-bonding interactions with His196, Tyr224, and Asn233, and is important for binding the carboxylate of **16** and **17**.

In order to investigate the inhibitory mechanism of **16** and **17**, we then sought to obtain crystal structures for complexes of VIM-2 with them (ESI Experimental section SE. 5 and Table S1† for details of crystallization and structure determinations). Co-crystallisation experiments yielded structures of VIM-2 in complex with **16** (1.40 Å) and **17** (1.93 Å) (Table S2†). VIM-2:**16** and VIM-2:**17** crystallized in the *P*2_1_22_1_ and *P*2_1_2_1_2_1_ space groups (Table S2†), respectively, with one molecule in the asymmetric unit (ASU); these space groups differ from those previously reported for VIM-2.^12,18,19^ In both structures, there was clear *F*_o_ – *F*_c_ density in the VIM-2 active site, into which **16** and **17** could be confidently modelled (Fig. S17†). The crystal structures reveal that **16** and **17** both bind adjacent to the zinc ions of VIM-2, but in a mode which does not involve direct zinc chelation (Fig. 3a–c), consistent with the NMR results (Fig. 2a and b). All of the atoms of the inhibitor are >4 Å from the zinc ions (Fig. 3c). The predicted binding modes of **16** and **17** are similar to those observed in the crystal structures (Fig. S18†), indicating that the virtual screening method, which searched for compounds likely to interact with catalytically important active site features, is a useful strategy for identification of new MBL-fold enzyme inhibitors.

The crystal structures reveal that **16** and **17** have very similar binding modes, in which the 3-oxoisoindoline-4-carboxylate heterocyclic core is positioned to make hydrophobic and electrostatic interactions with the VIM-2 active site features, including *via* π–π stacking interactions with Phe61 on the L3 loop, hydrogen-bond interactions with Arg228 and Asn233 on the L10 loop and water molecules (such as W3) (Fig. 3a and b and S19†). **16** (IC_50_ = 10.6 μM) appears positioned to make stronger hydrogen-bonding interactions with Arg228 (2.9 Å), Asn233 (2.8 Å), and a water molecule W2 (2.7 Å) (Fig. 3a and b and S19†) than **17** (IC_50_ = 32.8 μM), which may, in part, explain why it inhibits more potently. As revealed by analysis of the Cambridge Structural Database, zinc–ligand coordination bond lengths are generally less than 2.5 Å,^30^ thus **16** and **17** both do not directly chelate the VIM-2 active site zinc ions. A structural water molecule, W3, also observed in other crystal structures of VIM-2,^12,18–20^ is positioned to form hydrogen-bonding interactions with His196 (2.9 Å), Tyr224 (2.7 Å), and Asn233 (2.7 Å) and appears to be important to the binding of the carboxylate groups of **16** and **17** (Fig. 3d).

**Fig. 4:**
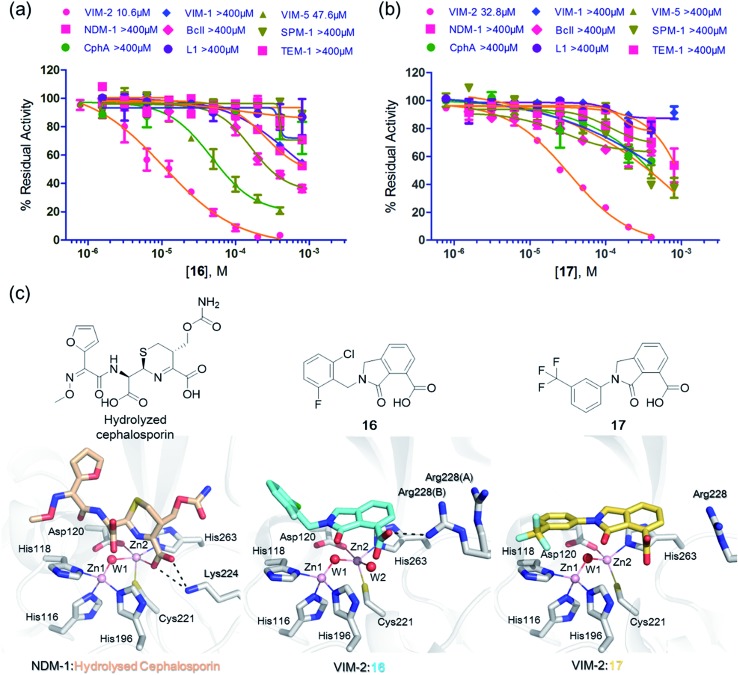
Compounds **16** and **17** mimic interactions made by substrates. Selectivity profiles of **16** (a) and **17** (b) for class B MBLs and the class A serine β-lactamase TEM-1 (for which no inhibition was observed). (c) Comparison of VIM2 structures in complex with **16** (PDB ID ; 5LE1) and **17** (PDB ID ; 5LCA) with that of a representative substrate intermediate of a hydrolysed cephalosporin in complex with NDM-1 (PDB ID ; 4RL0)^33^ indicates that **16** and **17** have related binding modes to the cephalosporin substrate. Docking studies imply the binding modes of **16** and **17** may mimic the binding mode of the substrate prior to β-lactam hydrolysis (Fig. S22†).

We then tested **16** and **17** against; the subclass B1 enzymes VIM-5, VIM-1, NDM-1, SPM-1, and BcII; the subclass B2 enzyme Cph-A; the subclass B3 enzyme L1; and the class A serine β-lactamase TEM-1 using our previously established screening platform^27^ to investigate their selectivity profiles (see ESI Experimental section SE. 3 and Table S3†). Notably, **16**, and to a greater extent, **17** have considerable selectivity for VIM-2 (Fig. 4a and b). Even for VIM-5, which is a close homolog of VIM-2 (sequence identity is 89.85%, Fig. S20a†),^31,32^ **16** and **17** showed lower inhibitory activities with IC_50_ values of 47.6 μM (10.6 μM for VIM-2) and >400 μM (32.8 μM for VIM-2), respectively (Fig. 3a and b and Table 2). As observed by crystallography, the active site features of VIM-2 (PDB ; 4BZ3)^18^ and VIM-5 (PDB ; 5A87)^32^ are highly similar with only three residues being different (Ile223_VIM-2_/Val223_VIM-5_, Tyr224_VIM-2_/Leu224_VIM-5_, and Glu225_VIM-2_/Ala225_VIM-5_) on the L10 loop (Fig. S20†). These substitutions may contribute to the small differences observed by crystallography in the conformations of the L10 loops of VIM-2 and VIM-5 (Fig. S20b†); however, differences in solution may be larger. Notably, a water molecule positioned similarly to W3 in VIM-2 (W3_VIM-2_), which we propose is important for binding of **16** and **17** (Fig. 3a–c), is apparent in the VIM-5 active site (W3_VIM-5_, Fig. S20b†); however, W3_VIM-5_ appears to be not so stable as W3_VIM-2_ (PDB ; 5A87). These differences may explain why **16** and **17** manifest substantial selectivity for VIM-2 over VIM-5 (Fig. 4).

**Table 2:** Structure–activity relationships of 3-oxoisoindoline-4-carboxylate derivatives for VIM-2, VIM-5, and VIM-1

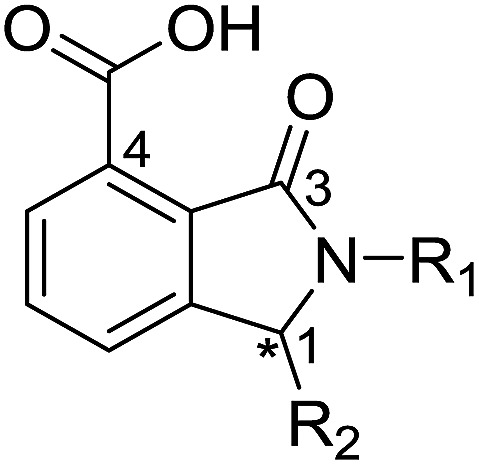					
Cpd ID^*a*^	R_1_	R_2_	IC_50_ (μM)/pIC_50_/s.e. log IC_50_ ^*b*^		
			VIM-2	VIM-5	VIM-1
**16**	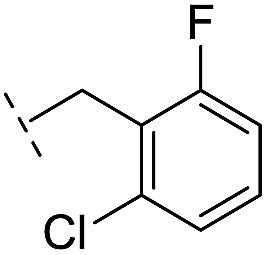	–H	10.6/4.98/0.119	47.6/4.32/0.079	>400/<3.4/—
**17**	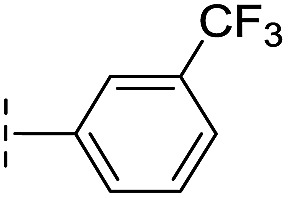	–H	32.8/4.48/0.055	>400/<3.4/—	>400/<3.4/—
**21**	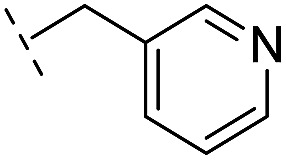	–H	349/3.46/0.15	>400/<3.4/—	>400/<3.4/—
**22**	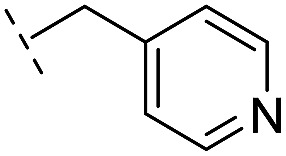	–H	>400/<3.4/—	>400/<3.4/—	>400/<3.4/—
**23**	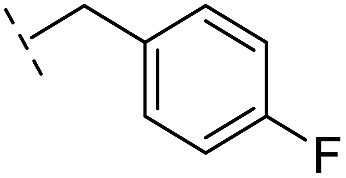	–H	250/3.60/0.17	>400/<3.4/—	>400/<3.4/—
**24**	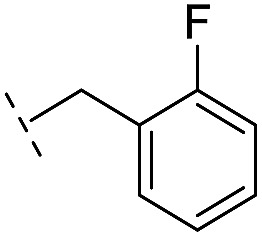	–H	47.1/4.33/0.037	127/3.90/0.075	>400/<3.4/—
**25**	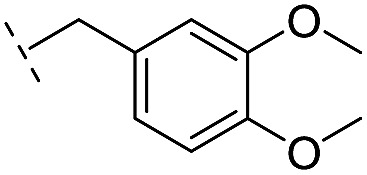	–H	320/3.50/0.096	>400/<3.4/—	>400/<3.4/—
**26**	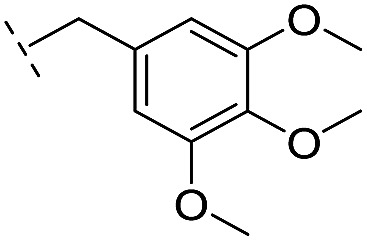	–H	>400/<3.4/—	>400/<3.4/—	>400/<3.4/—
**27**	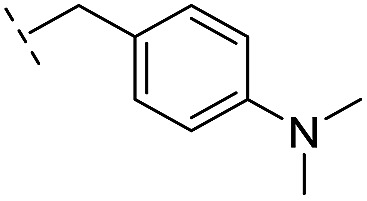	–H	176/3.76/0.079	>400/<3.4/—	>400/<3.4/—
**28**	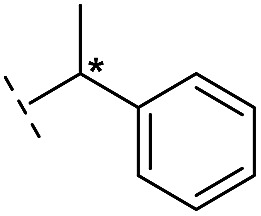	–H	168/3.78/0.153	>400/<3.4/—	>400/<3.4/—
**29**	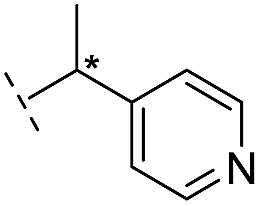	–H	229/3.64/0.062	>400/<3.4/—	>400/<3.4/—
**30**	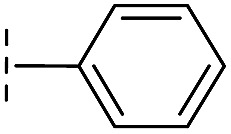	–H	17.8/4.75/0.039	>400/<3.4/—	>400/<3.4/—
**31**	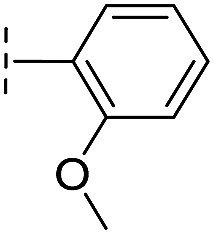	–H	133/3.88/0.056	>400/<3.4/—	>400/<3.4/—
**32**	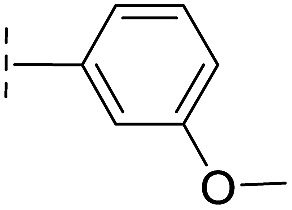	–H	39.3/4.41/0.029	>400/<3.4/—	>400/<3.4/—
**33**	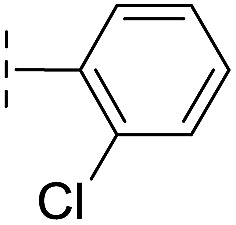	–H	74.0/4.13/0.057	>400/<3.4/—	>400/<3.4/—
**34**	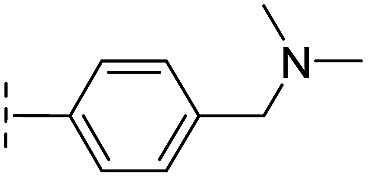	–H	60.3/4.22/0.093	>400/<3.4/—	>400/<3.4/—
**35**	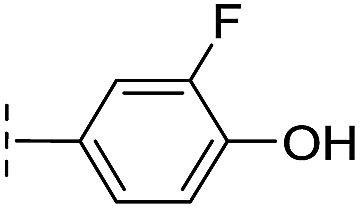	–H	7.7/5.11/0.071	>400/<3.4/—	>400/<3.4/—
**36**	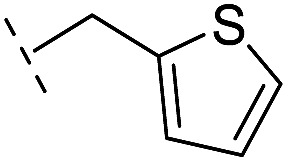	–H	153/3.82/0.138	180/3.75/0.116	>400/<3.4/—
**37**	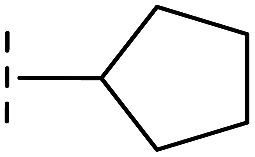	–H	68.1/4.17/0.079	>400/<3.4/—	>400/<3.4/—
**38**	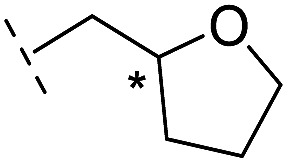	–H	>400/<3.4/—	>400/<3.4/—	>400/<3.4/—
**39**	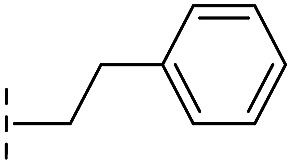	–H	71.9/4.14/0.082	>400/<3.4/—	>400/<3.4/—
**40**	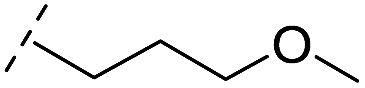	–H	>400/<3.4/—	>400/<3.4/—	>400/<3.4/—
**41**	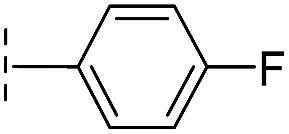	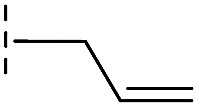	24.6/4.61/0.097	285/3.49/0.133	>400/<3.4/—
**42**	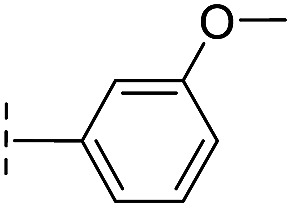	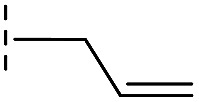	20.3/4.69/0.032	348/3.46/0.210	>400/<3.4/—
**43**	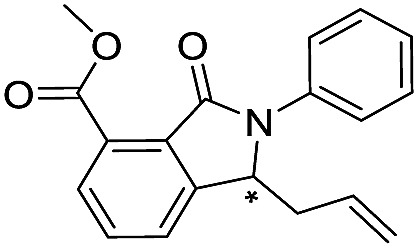		>400/<3.4/—	>400/<3.4/—	>400/<3.4/—

^*a*^Compounds **28**, **29**, **38**, **41**, **42**, and **43** were tested as racemic mixtures.

^*b*^The method for measuring IC_50_/pIC_50_ (*n* ≥ 3) values is described in ESI methods.^27^

We were attracted by the resemblance of the 3-oxoisoindoline-4-carboxylate scaffold to that of the bicyclic β-lactam MBL substrates, *e.g.* cephalosporins (Fig. 1 and 4c). Using molecular docking analyses, we observed that the bicyclic core of a cephalosporin (a representative bicyclic β-lactam substrate) is likely to form hydrogen-bonding interactions with Asn233 and the water molecule W3 (Fig. S22a†), in a similar manner to the 3-oxoisoindoline-4-carboxylate scaffold of **16** and **17**, as observed by our crystallographic analyses (Fig. S22b†). As shown in Fig. 4c, **16** and **17** also have a similar binding mode to that observed in a complex of NDM-1 with a ring-opened cephalosporin intermediate;^33^ the carboxylate groups of **16** and **17** are positioned to make electrostatic interactions with Arg228 in an analogous manner to that observed for the binding of the cephalosporin C-4 carboxylate to Lys224 (a structurally equivalent residue to Arg228) of NDM-1 (ref. 33) (Fig. 4c).

On superimposing the crystal structures of VIM-2:**16**, VIM-2:**17**, and di-Zn(ii) VIM-2, we observe that the L3 loop of VIM-2 appears closer to the zinc ions when **16** and **17** are bound to VIM-2 (3.50 Å and 3.81 Å movements of the C-α atoms of Asp62, respectively, Fig. S23a†), whilst the L10 loop is positioned further away from the zinc ions (1.39 Å and 1.53 Å difference in the C-α atom of Asn233, respectively, Fig. S23†). These results support the proposal that, at least in some cases, these loops contribute to the highly efficient nature of MBL catalysis by capturing potential substrates and delivering them to the zinc ion containing active site for hydrolysis.

**Fig. 5:**
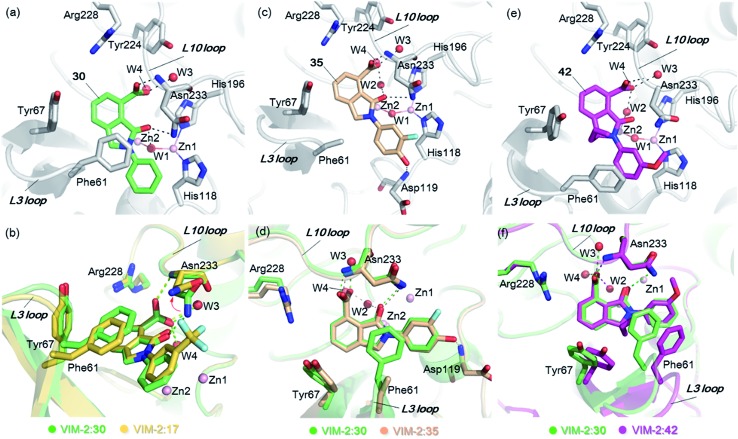
Crystallographic analyses reveals how the 3-oxoisoindoline-4-carboxylate derivatives bind to the VIM-2 MBL. (a) View from a crystal structure of VIM-2 in complex with **30** (PDB ID ; 5LCF). (b) Comparison of structures of VIM-2:**17** and VIM-2:**30** reveals the Asn233 side chain is unable to form hydrogen-bonds with the 3-oxoisoindoline-4-carboxylate of **17** due to its trifluoromethyl group, which may explain why **17** is less potent than **30** (Table 2). (c) View from a crystal structure of VIM-2 in complex with **35** (PDB ID ; 5LM6). (d) Comparison of VIM-2:**30** and VIM-2:**35** complex structures reveals **30** and **35** have the same binding mode. (e) View from a crystal structure of VIM-2 in complex with **42** (PDB ID ; 5LCH). (f) Comparison of structures of VIM-2:**30** and VIM-2:**42** reveals evidence for flexibility in the conformation of Tyr67 and Phe61 on the L3 loop, suggesting Tyr67 and Phe61 may play important roles in capturing substrates and delivering them to the zinc ions for hydrolysis.

From the results above, we propose that the 3-oxoisoindoline-4-carboxylate scaffold of **16** and **17** is an important factor contributing to the inhibitory potency and the selectivity for VIM-2. We carried out structure–activity relationship (SAR) studies using commercially available and synthesised 3-oxoisoindoline-4-carboxylate derivatives with different indole-*N*-substituents (R_1_) and/or *C*-1 substituents (R_2_) (compounds **21–43** in Table 2). We synthesized **34** and **35** *via* the routes in ESI methods SE. 2 and Scheme S1.† Compared to the hit compound **16** identified in initial screening (IC_50_ = 10.6 μM), which has a 2-chloro-6-fluorobenzyl moiety at the R_1_ position, compounds **21–29** with different substituted-benzyl moieties at the R_1_ position exhibited lower inhibitory activities against VIM-2. The results indicated that chloro- and fluoro-substituents at the *ortho* position of the *N*-benzyl group are important in binding. The VIM-2:**16** complex structure implies that the fluorine and chlorine atoms of **16** are likely positioned to form halogen-bonding interactions with Asn233 (2.88 Å), and Trp87 (3.41 Å), and Asp120 (3.34 Å), respectively (Fig. 3a and S19a†). Compounds bearing substituted phenyl moieties at the R_1_ position (**30–35**) displayed better VIM-2 inhibitory activities than **21–29**, some having comparable activity to **16** and **17**. Compound **30** is more potent than **17** (IC_50_ = 32.8 μM) with an IC_50_ value of 17.8 μM, although its *N*-phenyl group is unsubstituted. As revealed by subsequent crystallographic analyses of VIM-2 complexes, **30** and **17** have similar binding modes (Fig. 5a and b and 3b). However, compared with **17**, **30** is apparently better positioned to interact with the Asn233 side chain (the trifluoromethyl group of **17** hinders hydrogen bonding interactions between the Asn233 side chain and the 3-oxoisoindoline-4-carboxylate, Fig. 5b and S24†). Compound **35** (IC_50_ = 7.7 μM), which contains a 3-fluoro-4-hydroxyphenyl motif at its R_1_ position, was more potent against VIM-2 than compounds **16** and **17** (Table 2). As observed by crystallography, **35** has the same overall binding mode as **30** (Fig. 5c and d); compared with **30**, **35** is positioned to make additional hydrogen-bonding interactions with Asp119 (3.03 Å), as well as halogen-bonding interactions with Asn233 (3.75 Å) (Fig. 5c and d and S25†). In addition to various substituted-benzyl/phenyl groups, compounds **36–40**, containing thiophen-2-ylmethyl, cyclopentyl, tetrahydrofuran-2-yl, phenethyl, and 3-methoxypropyl at R_1_ position, respectively, were tested. These compounds displayed only moderate inhibitory potencies against VIM-2. **41** and **42**, which both have an allyl group at the R_2_ position, showed relatively good inhibitory activities against VIM-2 with IC_50_ values of 24.6 μM and 20.3 μM, respectively (Table 2). As observed in a VIM-2:**42** complex crystal structure, the allyl group of **42** is positioned to make hydrophobic interactions with Phe61 and Tyr67 (Fig. 5e and S26†).

Notably, comparison of structures of VIM-2:**30** and VIM-2:**42** reveals evidence for flexibility in the conformation of the L3 loop, suggesting that Tyr67 and Phe61 may be important in substrate/inhibitor capture (Fig. 5f). **43** showed much lower inhibitory potency, supporting the proposed role of the 3-oxoisoindoline-4-carboxylate motif in binding/inhibition. For VIM-5 and VIM-1, almost all the tested 3-oxoisoindoline-4-carboxylate derivatives (except **16**, **24**, and **36**) displayed low inhibitory activities, indicating that the 3-oxoisoindoline-4-carboxylate scaffold is an important factor (along with its precise substitution pattern) in determining selectivity for VIM-2. Together, these results led us to conclude that the 3-oxoisoindoline-4-carboxylate derivatives selectively inhibit the VIM-2 MBL *via* a mode which does not involve direct chelation with zinc ions.

## Discussion

The overall results clearly reveal the potential of a customized virtual screening approach targeting specific active site features for the identification of hit compounds for MBL/MBL-fold/metallo-enzymes. We successfully used an NMR-based approach employing di-Zn(ii)- and apo-VIM-2 to identify hits from virtual screening that bind to both the apo- and the di-Zn(ii) containing enzymes, hence are less likely to inhibit solely *via* direct zinc ion coordination. Indeed, the approach led to some compounds which bind *via* direct zinc ion chelation and some which do not, *e.g.* **16** and **17**, as supported by subsequent crystallographic analyses.

The combined biophysical analyses reveal 3-oxoisoindoline-4-carboxylate derivatives as a new class of MBL inhibitor, which do not, at least with the analysed compounds, strongly chelate the active site zinc ions. MBL selectivity profiling analyses showed 3-oxoisoindoline-4-carboxylate derivatives can be highly selective for VIM-2, even with respect to very closely related variants, *e.g.* VIM-5. The apparently narrow selectivity of the 3-oxoisoindoline-4-carboxylate derivatives may limit their use for potentiating β-lactam antibacterials, since clinically useful MBL inhibitors should be broad spectrum targeting multiple MBL types, at least the B1 subclass. However, the limited SAR results presented here suggest that increasing the potency and broadening the spectrum of the 3-oxoisoindoline-4-carboxylate inhibitors towards clinically relevant MBLs should be possible. Most importantly, the results reveal that non-metal-chelating inhibitors present a route to new types of selective MBL-fold enzyme inhibitors. This may be particularly useful in targeting human MBL-fold enzymes involved in cancer drug resistance,^8,9^ *e.g.* where highly selective inhibition is desirable. When coupled with appropriate assays, such selective inhibitors may be useful in profiling clinically observed MBL variants.

From a mechanistic perspective, the biophysical analyses are of interest because the crystallographically observed binding modes for the 3-oxoisoindoline-4-carboxylate inhibitors, to some extent, mimic those of intact β-lactam substrates to MBLs. In this regard, the interactions of the 3-oxoisoindoline-4-carboxylate inhibitors with the L3/L10 loops are of particular interest, since it is possible, at least in some cases, that these loops may contribute to the highly efficient nature of MBL catalysis by capturing potential substrates and delivering them to the zinc ion containing active site for hydrolysis.

## Conflict of interest

The authors declare no competing financial interest.
