# Supplementary material for: NMR-filtered virtual screening leads to non-metal chelating metallo-β-lactamase inhibitors
Source: Chem Sci. 2016 Dec 14;8(2):928–37. doi: 10.1039/c6sc04524c (PMC5369532; doi:10.1039/c6sc04524c)
Supplement: Supplementary file 1 [file SC-008-C6SC04524C-s001.pdf]

Electronic Supplementary Material (ESI) for Chemical Science.
This journal is © The Royal Society of Chemistry 2016

Supplementary Information

NMR-Filtered Virtual Screening Leads to Non-Metal Chelating Metallo-β-Lactamase Inhibitors

Guo-Bo Li[a,b], Martine I. Abboud[a], Jürgen Brem[a], Hidenori Someya[a,c], Christopher T. Lohans[a], Sheng-Yong Yang[d], James Spencer[e], David W. Wareham[f], Michael A. McDonough[a,*], Christopher J. Schofield[a,*]

[a] *Department of Chemistry, University of Oxford, 12 Mansfield Road, Oxford, OX1 3TA, UK.*

[b] *Key Laboratory of Drug Targeting and Drug Delivery System of Ministry of Education, West China School of Pharmacy, Sichuan University, Chengdu, 610041, China.*

[c] *Medicinal Chemistry Research Laboratories, New Drug Research Division, Otsuka Pharmaceutical Co., Ltd., 463-10 Kagasuno, Kawauchi-cho, Tokushima 771-0192, Japan.*

[d] *State Key Laboratory of Biotherapy/Collaborative Innovation Center for Biotherapy, West China Hospital, West China Medical School, Sichuan University, Sichuan 610041, China.*

[e] *School of Cellular and Molecular Medicine, Biomedical Sciences Building, University of Bristol, Bristol BS8 1TD, UK.*

[f] *Antimicrobial Research Group, Barts & The London School of Medicine and Dentistry, Queen Mary University of London, London, E1 2AT, UK.*

[*] *Correspondence: christopher.schofield@chem.ox.ac.uk (C. J. Schofield) or michael.mcdonough@chem.ox.ac.uk (M. A. McDonough).*

Contents

Supplementary Experimental Section 2
- SE. 1 Virtual Screen Details 2
- SE. 2 Chemistry 4
- SE. 3 Inhibition Assays 7
- SE. 4 NMR experiments 7
- SE. 5 Crystallography 8

Supplementary Figures 9
Supplementary Tables 40
References 42

Supplementary Experimental Section

SE. 1 Virtual Screen Details

The customized virtual screen protocol employed the following general steps (Fig. S3): (1) library preparation for docking; (2) docking simulation using AutoDock Vina[1]; (3) docking pose analysis/scoring using interaction fingerprints (IFPs) as defined below; (4) a second round of docking using GOLD[2] and docking pose analysis/scoring using IFPs; (5) manual analyses to select hits for biochemical validation. These steps are sequentially described below.

(1) *Library Preparation for Docking.* The chemical database from Vitas-M laboratory Ltd. (http://www.vitasmlab.com/) was used as the screening database; this contains more than 1,200,000 small-molecule compounds. The chemical structures (mol2 format) were downloaded from the ZINC database[3], because they are well prepared for docking in this database. The following rules were used to filter the chemical database by an in-house script with the aim of excluding non-drug-like compounds, and large complicated compounds that are not easy to screen by molecular docking: (i) Lipinski's rule of five (molecule weight $\leq$ 500; number of hydrogen bond donors $\leq$ 5; number of hydrogen bond acceptors $\leq$ 10; logP $\leq$ 5), (ii) number of polar atoms $\leq$ 10, and (iii) number of rotatable bonds $\leq$ 10. The mol2 formatted files for the ~800,000 compounds remaining were converted to pdbqt format for AutoDock Vina using the Raccoon script (http://autodock.scripps.edu/resources/raccoon)[4].

(2) *Molecular Docking Using AutoDock Vina*. AutoDock Vina was used for the virtual screen.[1] An X-ray crystal structure of VIM-2 in complex with 2-(4-fluorophenyl)carbonylbenzoic acid (PDB ID: 5ACX)[5] was used as the docking template. The two active site zinc ions and the bridging water molecule were included; other solvent molecules were removed. Gasteiger-Marsili charges were added to the protein model. Non-polar hydrogens were then merged onto their respective heavy atoms using AutoDockTools (http://autodock.scripps.edu/resources/adt). The grid center was set to coordinates of [x, y, z = -35.3, 8.8, 7.9] and the grid size was set to 25Å × 25Å × 25Å encompassing the entire VIM-2 active site. The other parameters for Vina were set as default. The docking simulations were carried out by parallel computing using high-performance computers in the Advanced Research Computing center, University of Oxford. Note, ~10% of compounds in the original database failed to complete docking, likely due to conflicts in input pdbqt formatted files and or limited computing time; These compounds were not pursued further.

(3) *Docking Pose Analysis/Scoring Using IFPs*. An IFP-based method was used to rescore the docking poses with the aim of searching for compounds likely to interact with key active site residues and metal ions. IFPs were defined by eight types of protein-ligand interactions comprising: those involved as hydrogen-bond donors (D), hydrogen-bond acceptors (A), positive charges (P), negative charges (N), face-to-face π-π stacking (F), edge-to-face π-π stacking (E), hydrophobic interactions (H), and metal-ligand interactions (M) as defined in our previously reported ID-Score method[6, 7]. A weighted reference IFPs for VIM-2 was established (Fig. S3c), in which catalytically important residues Phe61, Tyr67, Asp119, Arg228, Asn233 (BBL numbering) and the two active site zinc ions were included. The IFP scores for each of the docking poses were calculated using an in-house program, called IFP-Analyse. The similarity between the docking pose IFPs and reference IFPs value was compared using the following formula: $\text{IFP score} = \sum C_i * W_i \,/ \sum R_i * W_i$. C_i is derived from the sum of common bits between the docking pose's IFPs and the reference IFPs. R_i is derived from the reference IFPs. W_i is the corresponding weight for each interaction type.

(4) *Docking using GOLD and Pose Analyses/Scoring by IFPs*. The AutoDock Vina derived hits (>30,000) with an IFP score >0.4 were submitted to another round of docking simulation using GOLD[8] followed by pose analysis/scoring using the IFP method. More than 16,000 compounds with similar predicted binding poses based on IFP scores from both Vina and GOLD were retained.

(5) *Manual Analyses*. The resulting compounds were further inspected visually to check whether the predicted binding poses are reasonable and to select structurally diverse compounds. Due to cost consideration, only the top 20 commercially available compounds (out of >2000) were selected for testing against VIM-2 activity *in vitro* (see Table 1).

SE. 2 Chemistry.

Compounds **1-33**, **36-43** were from Vitas-M Laboratory Ltd. (Hong Kong) and used without purification. Compounds **34** and **35** were synthesized via the synthetic route shown in Scheme S1. The synthesis of **35** is representative.

Scheme S1.

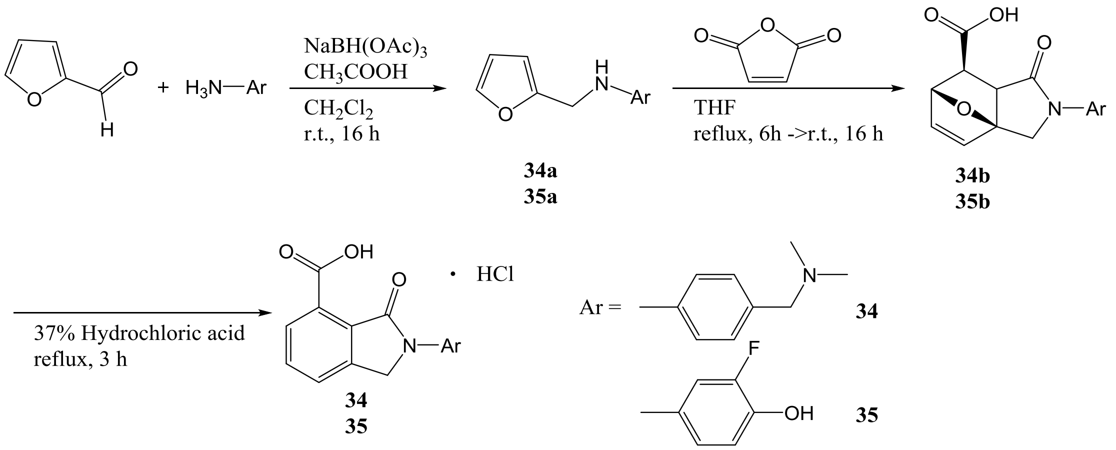

Synthesis of 2-fluoro-4-{(furan-2-ylmethyl)amino}phenol (35a) [9]

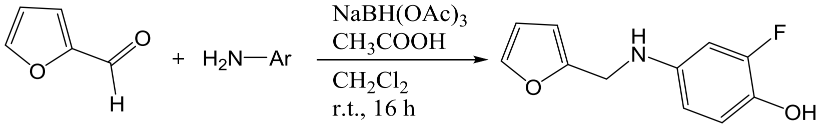

A solution of furan-2-carbaldehyde (0.12 mL, 1.5 mmol) in CH_2Cl_2 (50 mL) was placed in a 100-mL flask followed by addition of 4-amino-2-fluorophenol (0.23 g, 1.8 mmol). Glacial acetic acid (0.09 mL, 1.5 mmol) was added to the reaction mixture at 0 ˚C. The mixture was stirred for 10 min at room temperature. Sodium triacetoxyborohydride was added to the reaction mixture at 0 ˚C. After stirring for 22 h at room temperature, the reaction mixture was carefully poured into saturated $NaHCO_3$ aqueous solution. The products were extracted with ethyl acetate (10 mL × 3). The combined organic layers were concentrated using a rotary evaporator. Silica gel column purification (cyclohexane/ethyl acetate = 5:1) afforded **35a** (0.2 g, 0.97 mmol) as a red solid in 65% isolated yield.

2-Fluoro-4-{(furan-2-ylmethyl)amino}phenol (35a): Red solid. mp 118-120 ˚C; IR (neat) 3550, 3025, 1681, 1625, 1595, 1520, 145, 1244, 1225, 1072, 1011, 806, 723 cm^{-1}; ^{1}H NMR ($CDCl_3$, 400 MHz) δ 7.39 (dd, *J* = 2.0, 1.0 Hz, 1H), 6.85 (t, *J* = 9.0 Hz, 1H), 6.49 (dd, *J* = 12.5, 2.5 Hz, 1H), 6.39 (ddd, *J* = 8.5, 2.5, 1.5 Hz, 1H), 6.34 (dd, *J* = 3.0, 2.0 Hz, 1H), 6.24 (d, *J* = 3.0 Hz, 1H), 4.26 (s, 2H); ^{13}C NMR ($CDCl_3$, 100 MHz) δ 152.78, 152.39, 150.43, 142.05, 135.62 (d, *J* = 14.5 Hz, 1C), 117.91 (d, *J* = 3.0 Hz, 1C), 110.36, 109.88 (d, *J* = 2.5 Hz, 1C), 107.20, 101.49 (d, *J* = 22.0 Hz, 1C), 42.21; HRMS:

(ESI+): calcd for $C_{11}H_{11}O_2NF^+$ $[M+H]^+$ 208.0768; found 208.0773.

Synthesis of 2-(3-fluoro-4-hydroxyphenyl)-1-oxo-1,2,3,6,7,7a-hexahydro-3a,6-epoxyisoindole-7-carboxylic acid (35b) [9]

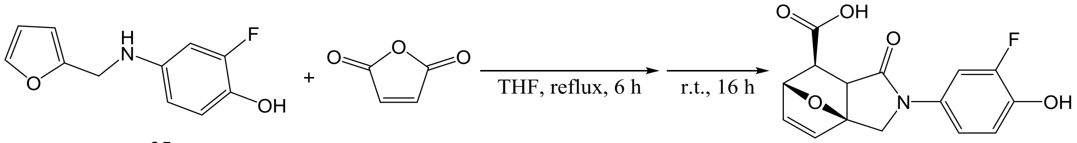

35a **35b**

A solution of **35a** (0.17 g, 0.8 mmol) in tetrahydrofuran (2 mL) was placed in a 50-mL flask. Maleic anhydride (0.08 g, 0.8 mmol) was added to the solution at room temperature. The reaction mixture was refluxed for 6 h, then the mixture was stirred 16 h at room temperature. Diethyl ether was added to the reaction mixture to form a precipitate. The precipitate was collected by filtration, washed with diethyl ether, and dried to afford the compound **35b** (0.18 g, 0.58 mmol) as a white solid in 71% yield with a small amount of by-products.

2-(3-Fluoro-4-hydroxyphenyl)-1-oxo-1,2,3,6,7,7a-hexahydro-3a,6-epoxyisoindole-7-carboxylic acid (35b): White solid. mp 192-195 ˚C; IR (neat) 3050, 1734, 1675, 1526, 1387, 1317, 1213, 883, 826, 714 cm^{-1}; ^1H NMR (MeOD, 500 MHz) δ 7.49 (dd, *J* = 13.0, 2.5 Hz, 1H), 7.14 (ddd, *J* = 9.0, 2.5, 1.5 Hz, 1H), 6.94 (dd, *J* = 9.5, 9.0 Hz, 1H), 6.68 (d, *J* = 6.0 Hz, 1H), 6.53 (dd, *J* = 6.0, 2.0 Hz, 1H), 5.15 (d, *J* = 2.0 Hz, 1H), 4.56 (d, *J* = 11.5 Hz, 1H), 4.06 (d, *J* = 11.5 Hz, 1H), 3.10 (d, *J* = 9.0 Hz, 1H), 2.82 (d, *J* = 9.0 Hz, 1H); ^{13}C NMR (MeOD, 125 MHz) δ 174.42, 171.18, 151.78, 149.87, 142.40 (d, *J* = 13.0 Hz, 1C), 136.56, 135.14, 131.15 (d, *J* = 9.0 Hz, 1C), 117.07 (d, *J* = 12.5 Hz, 1C), 109.88 (d, *J* = 22.5 Hz, 1C), 88.08, 82.09, 51.84, 50.40, 45.21; HRMS: (ESI+): calcd for $C_{15}H_{13}O_5NF^+$ $[M+H]^+$ 306.0772; found 306.0773.

Synthesis of 2-(3-fluoro-4-hydroxyphenyl)-3-oxoisoindoline-4-carboxylic acid (35)[9]

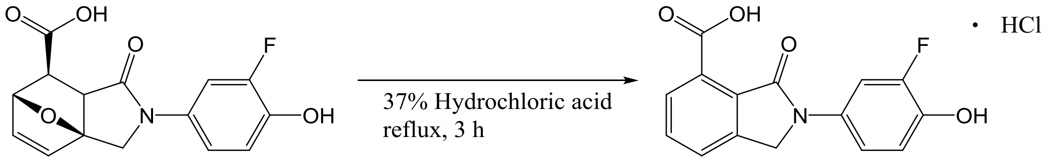

35b **35**

35b (0.13 g, 0.8 mmol) was placed in a 50 mL flask, and dissolved in 37% hydrochloric acid (1.5 mL). The reaction mixture was refluxed for 3 h, then the solvent was removed using a rotary evaporator. The crude material was dissolved in methanol, followed by adding diethyl ether to form precipitate. The precipitate was collected by filtration, washed with diethyl ether and dried to obtain the desired product **35** (0.08 g, 0.24 mmol) as hydrochloride salt in 55% isolated yield.

2-(3-Fluoro-4-hydroxyphenyl)-3-oxoisoindoline-4-carboxylic acid (35): Brown

solid. mp 249-250 ˚C; IR (neat) 3050, 1694, 1600, 1518, 1487, 1287, 1167, 780, 748 cm^{-1}; ^{1}H NMR (DMSO-d6, 500 MHz) δ 10.12 (br-s, 1H, -COOH), 8.13 (d, *J* = 7.5 Hz, 1H), 7.94 (d, *J* = 7.0 Hz, 1H), 7.87 (t, *J* = 7.5 Hz, 1H), 7.74 (dd, *J* = 13.0, 2.5 Hz, 1H), 7.46 (ddd, *J* = 9.0, 2.5, 1.0 Hz, 1H), 7.07 (t, *J* = 9.5 Hz, 1H), 5.16 (s, 2H); ^{13}C NMR (DMSO-d6, 125 MHz) δ 168.10, 165.71, 151.82, 149.90, 143.62 (d, *J* = 12.0 Hz, 1C), 143.04, 133.23, 131.82, 129.80 (d, *J* = 9.0 Hz, 1C), 129.63 (d, *J* = 16.5 Hz, 1C), 127.65, 118.57 (d, *J* = 3.0 Hz, 1C), 118.19 (d, *J* = 4.0 Hz, 1C), 110.83 (d, *J* = 22.5 Hz, 1C), 52.89; HRMS: (ESI+): calcd for $C_{15}H_{11}O_4NF^+$ $[M+H]^+$ 288.0667; found 288.0663.

Compounds **34a**, **34b** and **34** were obtained following the general procedure described above.

4-{(Dimethylamino)methyl}-N-(furan-2-ylmethyl)aniline (34a):

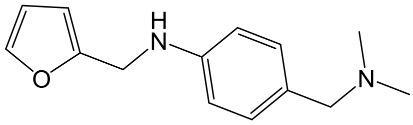

34a

Brown oil. 94% yield (0.13 g, 0.56 mmol); IR (neat) 2929, 2858, 2812, 2768, 1614, 1525, 1456, 1317, 1180, 1145, 1011, 851, 804, 725cm^{-1}; ^{1}H NMR ($CDCl_3$, 500 MHz) δ 7.39 (d, *J* = 5.0 Hz, 1H), 7.14 (d, *J* = 10.0 Hz, 2H), 6.66 (d, *J* = 10.0 Hz, 2H), 6.35 (d, *J* = 5.0 Hz, 1H), 6.26 (d, *J* = 5.0 Hz, 1H), 4.33 (s, 2H), 4.02 (br-s, 1H), 3.38 (s, 2H), 2.26 (s, 6H); ^{13}C NMR ($CDCl_3$, 125 MHz) δ 152.73, 146.88, 141.92, 130.38, 127.44, 112.97, 110.34, 107.00, 63.64, 44.88, 41.54; HRMS: (ESI+): calcd for $C_{14}H_{19}ON_2^+$ $[M+H]^+$ 231.1492; found 231.1491.

2-{4-(Dimethylaminomethyl)phenyl}-1-oxo-1,2,3,6,7,7a-hexahydro-3a,6-epoxyiso indole-7-carboxylic acid (**34b**):

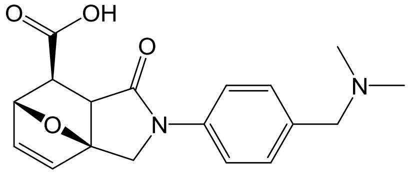

34b

Brown solid. 98% yield (0.16 g, 0.49 mmol); mp 180-182 ˚C; IR (neat) 3572, 1699, 1614, 1583, 1516, 1467, 1350, 1196, 851, 812 719, 685 cm^{-1}; ^{1}H NMR (MeOD, 400 MHz) δ 7.87 (d, *J* = 8.5 Hz, 2H), 7.62 (d, *J* = 8.5 Hz, 2H), 6.65 (d, *J* = 5.5 Hz, 1H), 6.56 (dd, *J* = 5.5, 1.5 Hz, 1H), 5.12 (d, *J* = 1.5 Hz, 1H), 4.58 (d, *J* = 11.5 Hz, 1H), 4.23–4.17 (m, 3H), 3.06 (d, *J* = 9.0 Hz, 1H), 2.76 (d, *J* = 5.0 Hz, 1H), 2.72 (s, 6H); ^{13}C NMR (MeOD, 100 MHz) δ 177.56, 171.87, 140.74, 137.13, 135.37, 134.45, 131.16, 126.46, 119.63, 87.41, 82.53, 59.90, 51.45, 49.30, 41.17; HRMS: (ESI+): calcd for $C_{18}H_{21}O_4N_2^+$ $[M+H]^+$ 329.1496; found 329.1495.

2-{4-(Dimethylaminomethyl)phenyl}-3-oxoisoindoline-4-carboxylic acid (34):

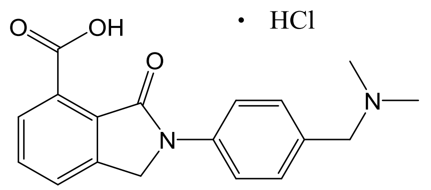

34

Brown solid. 64% yield (0.076 g, 0.22 mmol); mp 276-278 ˚C; IR (neat) 3400, 2550, 1705, 1650, 1518, 1398, 1310, 1288, 1016, 947, 804, 743, 692 cm^{-1}; ^1H NMR (MeOD, 500 MHz) δ 7.76 (d, J = 8.5 Hz, 2H), 7.75–7.40 (m, 3H), 7.26 (d, J = 8.5 Hz, 2H), 4.81 (s, 2H), 3.41 (s, 2H), 2.18 (s, 6H); ^{13}C NMR (MeOD, 125 MHz) δ 179.00, 167.30, 141.45, 138.72, 133.32, 131.75, 130.63, 130.01, 127.47, 122.09. 119.87, 114.89, 62.75, 50.48, 43.61; HRMS: (ESI+): calcd for $C_{18}H_{19}O_3N_2^+$ $[M+H]^+$ 311.1390; found 311.1385.

The ^1H NMR and ^{13}C NMR spectra for compounds **34a**, **34b**, **34**, **35a**, **35b**, and **35** are given in Figures S29-38.

SE. 3 Inhibition Assays

Except where noted, recombinant forms of NDM-1, VIM-2, SPM-1, IMP-1, and BcII MBLs were produced in *Escherichia coli* and assays were carried out as described previously[10]. The IC_{50} values of all the compounds for VIM-2 were determined using the FC-5 based assay[10]. For compounds **6**, **7**, **12**, **13**, **16**, **17**, **18**, and **20**, which have IC_{50} values < 400 μM, their inhibitory activities were further tested with different zinc concentrations (0 μM, 1 μM, and 100 μM, see Fig. S8). The selectivity profiles of compounds of interest against other B1 MBLs including VIM-5[11], VIM-1[10], NDM-1, SPM-1, and BcII, and TEM-1[12] (class A SBL) were determined using the same method as described above. The inhibitory activities of these compounds against CphA (B2 MBL)[13] and L1 (B3 MBL)[14] were determined using meropenem and nitrocefin, respectively. The IC_{50} values were determined for compounds showing inhibition >30% at 100 μM. The details regarding enzyme concentrations, substrate concentration are in Table S1.

SE. 4 NMR experiments

Nuclear Magnetic Resonance (NMR) spectra were recorded using a Bruker AVIII 600 MHz NMR spectrometer equipped with a BB-$^{19}F/^{1}H$ Prodigy N_2 cryoprobe using 5 mm diameter NMR tubes (Norell). Typical experimental parameters for Carr-Purcell-Meiboom-Gill (CPMG) NMR spectroscopy were: total echo time, 40 ms; relaxation delay, 2 s; and number of transients, 128. The PROJECT-CPMG sequence (90°x−[τ−180°y−τ−90°y−τ−180°y−τ]n−acq) was applied. [15] Water suppression was achieved by pre-saturation. Data were processed with Bruker 3.1 software. Prior to

Fourier transformation, data were multiplied with an exponential function with 0.5 Hz line broadening. Unless otherwise stated, the assay mixtures contained 50 µM di-Zn(II)-VIM-2 supplemented with 50 µM Zn(II) or 50 µM apo-VIM-2, and 50 µM of the compound to be studied buffered with 50 mM Tris-D11 (pH 7.5) and 0.02 % NaN_3 in 90 % H_2O and 10 % D_2O.

SE. 5 Crystallography

Structures of VIM-2 in complex with compounds **16**, **17**, **30** or **42** were obtained by co-crystallization; the structure of the VIM-2:**35** was obtained by soaking. Purified VIM-2 proteins were freshly prepared to a concentration of 22.36 mg/mL (except for VIM-2:**35** crystals – see below) in crystallization buffer (50 mM HEPES, pH 7.5, 100 mM NaCl), followed by adding 1 mM tris(2-carboxyethyl)phosphine (TCEP) and 5 mM compound. The protein-compound mixtures were co-crystallized using the sitting drop vapour diffusion method in 96 well 3-subwell Intelliplates® (Art Robbins). The reservoir buffer for growing crystals 21% - 27% polyethylene glycol (PEG) 3350 and 0.1 M magnesium formate. Crystals used for soaking in **35** were obtained as reported[12]. Crystals appeared after several days. The crystals were cryoprotected with 25% (v/v) glycerol, and flash-cooled in liquid nitrogen. Data were then collected on single crystals in-house or at the Diamond Light Source synchrotron beamline. Initial phases were obtained by the molecular replacement (MR) method[16] using the PHASER[16] subroutine within PHENIX[17], with the structure of VIM-2 (PDB code: 4BZ3) as the search model. Crystallographic structure refinements were carried out by iterative rounds of model building using Coot[18] and maximum likelihood restrained refinement using PHENIX. Crystallization conditions are in Table S2, and data collection and refinement statistics are in Table S3 and S4.

Protein Structure Accession Number. Coordinates and structure factors for structures of VIM-2:**16**, VIM-2:**17**, VIM-2:**30**, VIM-2:**35** and VIM-2:**42** have been deposited in the Protein Data Bank with the accession codes of 5LE1, 5LCA, 5LCF, 5LM6 and 5LCH, respectively.

Supplementary Figures

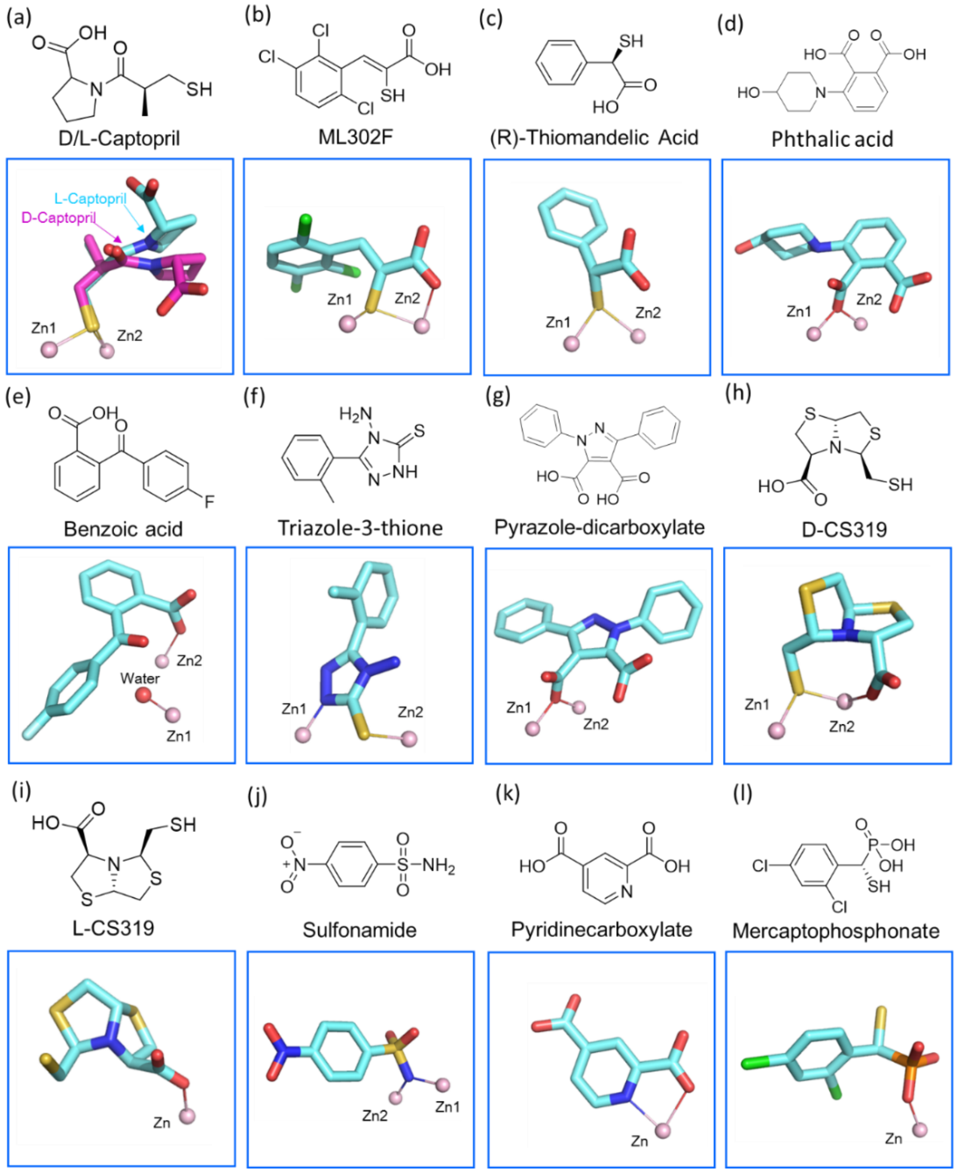

Fig. S1 Views from reported MBL protein crystal structures in complex with inhibitors (Part I). (a) Class B1 MBL VIM-2 in complex with L-captopril (PDB: 4C1D) or D-captopril (PDB: 4C1E)[19]; (b) VIM-2 in complex with ML302F (PDB: 4PVO)[12]; (c) class B1 MBL BcII in complex with (R)-thiomandelic acid (PDB: 2M5D)[20]; (d) class B1 MBL IMP-1 in complex with phthalic acid (PDB: 3WXC)[21]; (e) VIM-2 in complex with benzoic acid (PDB: 5ACX)[5]; (f) class B3 MBL L1 in complex with the triazole-3-thione scaffold (PDB: 2HB9)[14]; (g) L1 in complex with pyrazole-dicarboxylate (PDB: 2GFJ)[14]; (h) IMP-1 in complex with D-CS319 (PDB: 5EV8)[22]; (i) class B2 MBL Sfh-I in complex with L-CS319 (PDB: 5EW0)[22]; (j) class B3 MBL BJP-1 in complex with 4-nitrobenzene-sulfonamide (PDB: 3M8T)[23]; (k) class B2 MBL CphA in complex with pyridinecarboxylate (PDB: 2GKL)[13]; (l) CphA in complex with mercaptophosphonate (PDB: 3IOG)[24].

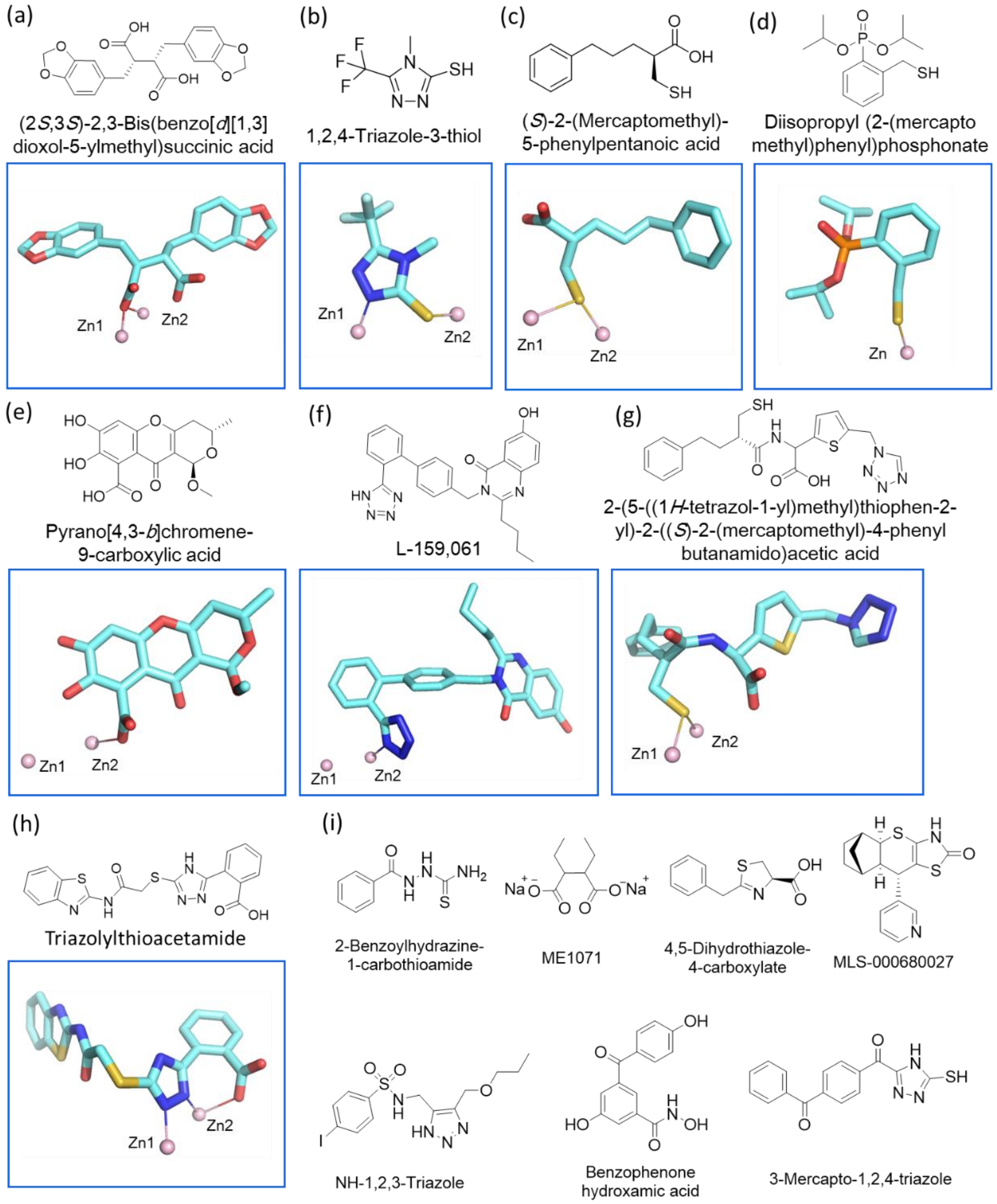

Fig. S2 Views from crystal structures of reported MBL inhibitors (Part II). (a) IMP-1 in complex with succinic acid (PDB: 1JJT)[25]; (b) VIM-2 in complex with triazole-3-thiol (PDB: 5ACW)[5]; (c) VIM-2 in complex with (R)-phenylpentanoic acid (PDB: 2YZ3)[26]; (d) CphA in complex with mercaptophosphonate (PDB: 3IOF)[24]; (e) CfiA in complex with pyrano[4,3-b]chromene-9-carboxylate (PDB: 1HLK)[27]; (f) CfiA in complex with L-159,061 (PDB: 1A8T)[28]; (g) IMP-1 in complex with a mercaptocarboxylate inhibitor (PDB: 1DD6)[29]; (h) VIM-2 in complex with a triazolylthioacetamide inhibitor (PDB: 5LSC)[30]; (i) Chemical structures of other reported MBL inhibitors[30-35], for which binding modes are unknown.

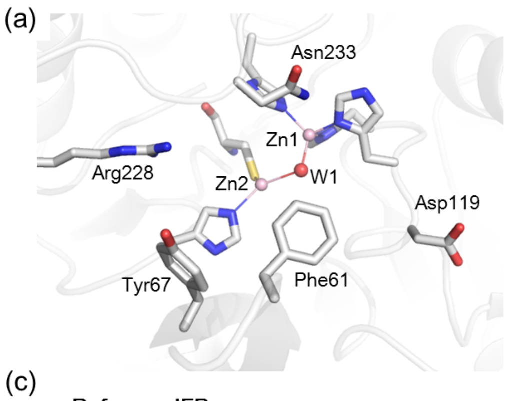

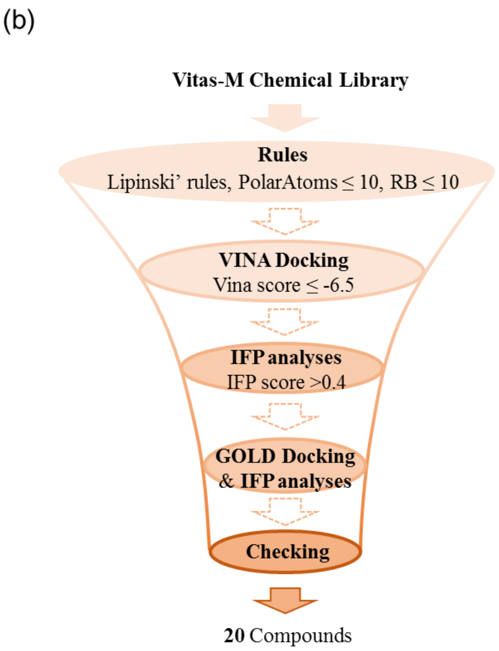

(c) Reference IFPs

Residue	D	A	P	N	F	E	H	M	Weight
Zn1	0	0	0	0	0	0	0	1	2
Zn2	0	0	0	0	0	0	0	1	2
Phe61	0	0	0	0	1	1	1	0	1
Tyr67	0	0	0	0	1	1	1	0	1
Asp119	0	1	0	1	0	0	0	0	1
Arg228	1	0	1	0	0	0	0	0	2
Asn233	1	0	0	0	0	0	0	0	1

Fig. S3 VIM-2 binding site features used in the virtual screen. (a) Binding site and key residues of VIM-2. (b) Virtual screen workflow. (c) The reference molecular interaction fingerprint (IFP) defined for the VIM-2 active site that was used in the virtual screen; Types of protein-ligand interactions D: hydrogen-bonding donor; A: hydrogen-bonding acceptor; P: positively charged feature; N: negatively charged feature; F: face-to-face π-π stacking interaction; E: edge-to-face π-π stacking interaction; H: hydrophobic interaction; M: metal-ligand interaction; Weight: the weight coefficient for key residues and zinc ions in the active site were defined and used to generate an IFP for each docked pose. The protein-ligand interactions were calculated *via* similar methods as used in previous work[6].

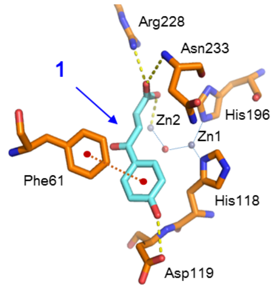

IFPscore = 0.5882

Residue	D	A	P	N	F	E	H	M
Zn1	0	0	0	0	0	0	0	0
Zn2	0	0	0	0	0	0	0	1
Phe61	0	0	0	0	1	0	1	0
Tyr67	0	0	0	0	0	0	0	0
Asp119	1	1	0	0	0	0	0	0
Arg228	1	0	1	0	0	0	0	0
Asn233	1	0	0	0	0	0	0	0

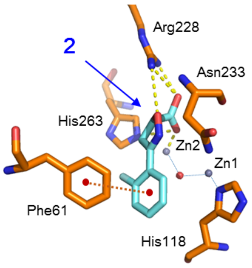

IFPscore = 0.4706

Residue	D	A	P	N	F	E	H	M
Zn1	0	0	0	0	0	0	0	0
Zn2	0	0	0	0	0	0	0	1
Phe61	0	0	0	0	1	0	1	0
Tyr67	0	0	0	0	0	0	0	0
Asp119	0	0	0	0	0	0	0	0
Arg228	1	0	1	0	0	0	0	0
Asn233	0	0	0	0	0	0	0	0

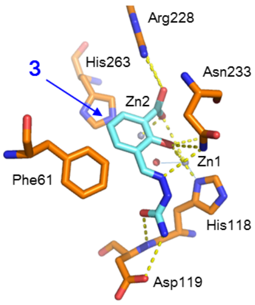

IFPscore = 0.6471

Residue	D	A	P	N	F	E	H	M
Zn1	0	0	0	0	0	0	0	1
Zn2	0	0	0	0	0	0	0	1
Phe61	0	0	0	0	0	0	1	0
Tyr67	0	0	0	0	0	0	0	0
Asp119	1	1	0	0	0	0	0	0
Arg228	1	0	1	0	0	0	0	0
Asn233	1	0	0	0	0	0	0	0

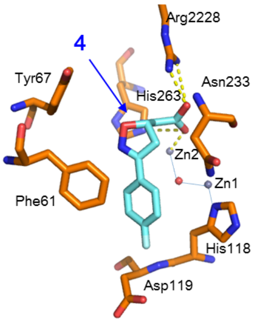

IFPscore = 0.5294

Residue	D	A	P	N	F	E	H	M
Zn1	0	0	0	0	0	0	0	0
Zn2	0	0	0	0	0	0	0	1
Phe61	0	0	0	0	1	0	1	0
Tyr67	0	0	0	0	0	0	0	0
Asp119	0	0	0	0	0	0	0	0
Arg228	1	0	1	0	0	0	0	0
Asn233	1	0	0	0	0	0	0	0

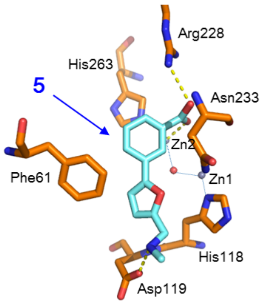

IFPscore = 0.5294

Residue	D	A	P	N	F	E	H	M
Zn1	0	0	0	0	0	0	0	0
Zn2	0	0	0	0	0	0	0	1
Phe61	0	0	0	0	0	0	1	0
Tyr67	0	0	0	0	0	0	0	0
Asp119	0	1	0	1	0	0	0	0
Arg228	1	0	1	0	0	0	0	0
Asn233	0	0	0	0	0	0	0	0

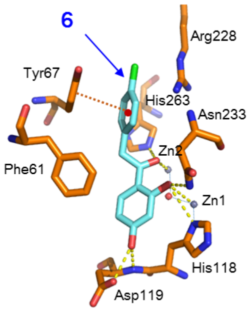

IFPscore = 0.4118

Residue	D	A	P	N	F	E	H	M
Zn1	0	0	0	0	0	0	0	0
Zn2	0	0	0	0	0	0	0	1
Phe61	0	0	0	0	0	0	1	0
Tyr67	0	0	0	0	1	0	1	0
Asp119	1	1	0	0	0	0	0	0
Arg228	0	0	0	0	0	0	0	0
Asn233	1	0	0	0	0	0	0	0

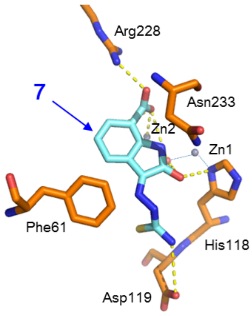

IFPscore = 0.4706

Residue	D	A	P	N	F	E	H	M
Zn1	0	0	0	0	0	0	0	0
Zn2	0	0	0	0	0	0	0	1
Phe61	0	0	0	0	0	0	1	0
Tyr67	0	0	0	0	0	0	0	0
Asp119	0	0	0	0	0	0	0	0
Arg228	1	0	1	0	0	0	0	0
Asn233	1	0	0	0	0	0	0	0

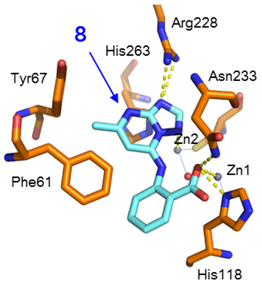

IFPscore = 0.4118

Residue	D	A	P	N	F	E	H	M
Zn1	0	0	0	0	0	0	0	1
Zn2	0	0	0	0	0	0	0	0
Phe61	0	0	0	0	0	0	1	0
Tyr67	0	0	0	0	0	0	1	0
Asp119	0	0	0	0	0	0	0	0
Arg228	1	0	0	0	0	0	0	0
Asn233	1	0	0	0	0	0	0	0

Fig. S4 Docking poses of compounds **1-8** and their corresponding interaction fingerprints (IFPs) generated in the virtual screening.

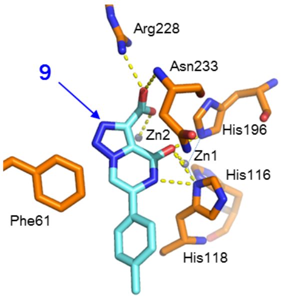

IFPscore = 0.4706

Residue	D	A	P	N	F	E	H	M
Zn1	0	0	0	0	0	0	0	0
Zn2	0	0	0	0	0	0	0	1
Phe61	0	0	0	0	0	0	1	0
Tyr67	0	0	0	0	0	0	0	0
Asp119	0	0	0	0	0	0	0	0
Arg228	1	0	1	0	0	0	0	0
Asn233	1	0	0	0	0	0	0	0

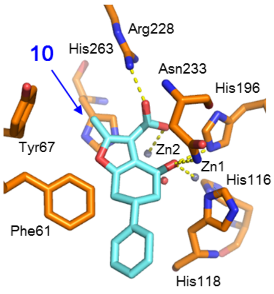

IFPscore = 0.4706

Residue	D	A	P	N	F	E	H	M
Zn1	0	0	0	0	0	0	0	0
Zn2	0	0	0	0	0	0	0	1
Phe61	0	0	0	0	1	0	1	0
Tyr67	0	0	0	0	0	0	0	0
Asp119	0	0	0	0	0	0	0	0
Arg228	1	0	1	0	0	0	0	0
Asn233	0	0	0	0	0	0	0	0

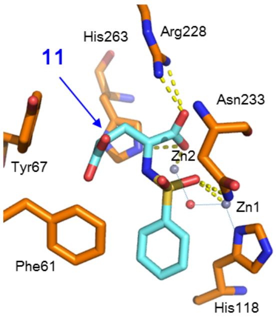

IFPscore = 0.5294

Residue	D	A	P	N	F	E	H	M
Zn1	0	0	0	0	0	0	0	0
Zn2	0	0	0	0	0	0	0	1
Phe61	0	0	0	0	0	0	1	0
Tyr67	0	0	0	0	0	0	1	0
Asp119	0	0	0	0	0	0	0	0
Arg228	1	0	1	0	0	0	0	0
Asn233	1	0	0	0	0	0	0	0

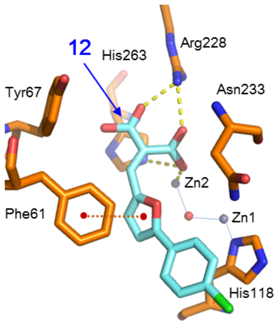

IFPscore = 0.5294

Residue	D	A	P	N	F	E	H	M
Zn1	0	0	0	0	0	0	0	0
Zn2	0	0	0	0	0	0	0	1
Phe61	0	0	0	0	1	0	1	0
Tyr67	0	0	0	0	0	0	0	0
Asp119	0	0	0	0	0	0	0	0
Arg228	1	0	1	0	0	0	0	0
Asn233	1	0	0	0	0	0	0	0

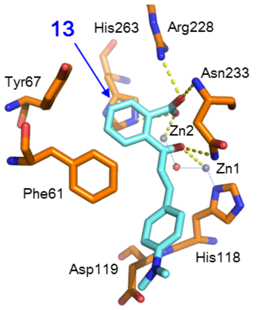

IFPscore = 0.5294

Residue	D	A	P	N	F	E	H	M
Zn1	0	0	0	0	0	0	0	0
Zn2	0	0	0	0	0	0	0	1
Phe61	0	0	0	0	0	0	1	0
Tyr67	0	0	0	0	0	0	1	0
Asp119	0	0	0	0	0	0	0	0
Arg228	1	0	1	0	0	0	0	0
Asn233	1	0	0	0	0	0	0	0

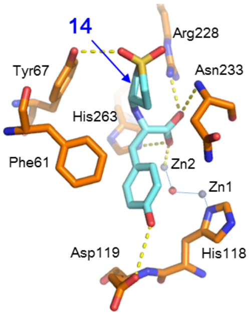

IFPscore = 0.4706

Residue	D	A	P	N	F	E	H	M
Zn1	0	0	0	0	0	0	0	0
Zn2	0	0	0	0	0	0	0	1
Phe61	0	0	0	0	0	0	1	0
Tyr67	1	0	0	0	0	0	0	0
Asp119	0	0	0	0	0	0	0	0
Arg228	1	0	1	0	0	0	0	0
Asn233	1	0	0	0	0	0	0	0

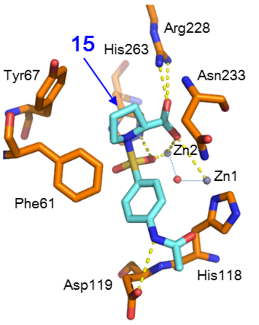

IFPscore = 0.4706

Residue	D	A	P	N	F	E	H	M
Zn1	0	0	0	0	0	0	0	0
Zn2	0	0	0	0	0	0	0	1
Phe61	0	0	0	0	0	0	1	0
Tyr67	0	0	0	0	0	0	0	0
Asp119	0	1	0	0	0	0	0	0
Arg228	1	0	1	0	0	0	0	0
Asn233	0	0	0	0	0	0	0	0

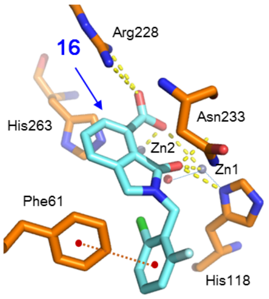

IFPscore = 0.5294

Residue	D	A	P	N	F	E	H	M
Zn1	0	0	0	0	0	0	0	0
Zn2	0	0	0	0	0	0	0	1
Phe61	0	0	0	0	1	0	1	0
Tyr67	0	0	0	0	0	0	0	0
Asp119	0	0	0	0	0	0	0	0
Arg228	1	0	1	0	0	0	0	0
Asn233	1	0	0	0	0	0	0	0

Fig. S5 Docking poses of compounds **9-16** and their corresponding interaction fingerprints (IFPs) generated in the virtual screening.

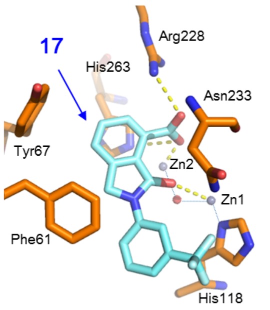

IFPscore = 0.5294

Residue	D	A	P	N	F	E	H	M
Zn1	0	0	0	0	0	0	0	0
Zn2	0	0	0	0	0	0	0	1
Phe61	0	0	0	0	0	0	1	0
Tyr67	0	0	0	0	0	0	1	0
Asp119	0	0	0	0	0	0	0	0
Arg228	1	0	1	0	0	0	0	0
Asn233	1	0	0	0	0	0	0	0

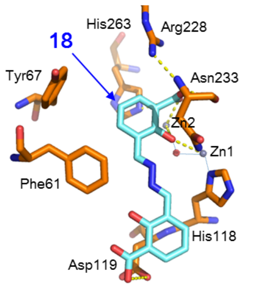

IFPscore = 0.5294

Residue	D	A	P	N	F	E	H	M
Zn1	0	0	0	0	0	0	0	1
Zn2	0	0	0	0	0	0	0	1
Phe61	0	0	0	0	0	0	1	0
Tyr67	0	0	0	0	0	0	0	0
Asp119	0	0	0	0	0	0	0	0
Arg228	1	0	1	0	0	0	0	0
Asn233	0	0	0	0	0	0	0	0

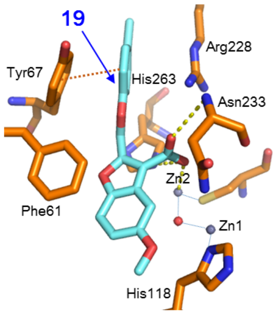

IFPscore = 0.4706

Residue	D	A	P	N	F	E	H	M
Zn1	0	0	0	0	0	0	0	0
Zn2	0	0	0	0	0	0	0	1
Phe61	0	0	0	0	0	0	0	0
Tyr67	0	0	0	0	1	0	1	0
Asp119	0	0	0	0	0	0	0	0
Arg228	1	0	1	0	0	0	0	0
Asn233	0	0	0	0	0	0	0	0

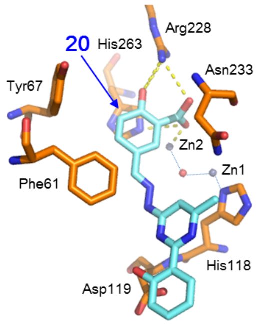

IFPscore = 0.4706

Residue	D	A	P	N	F	E	H	M
Zn1	0	0	0	0	0	0	0	0
Zn2	0	0	0	0	0	0	0	1
Phe61	0	0	0	0	0	0	1	0
Tyr67	0	0	0	0	0	0	0	0
Asp119	0	1	0	0	0	0	0	0
Arg228	1	0	1	0	0	0	0	0
Asn233	0	0	0	0	0	0	0	0

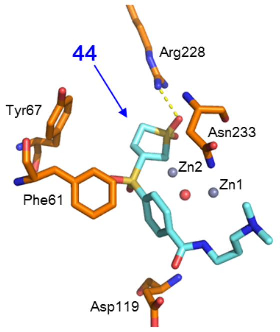

IFPscore = 0.1765

Residue	D	A	P	N	F	E	H	M
Zn1	0	0	0	0	0	0	0	0
Zn2	0	0	0	0	0	0	0	0
Phe61	0	0	0	0	0	0	1	0
Tyr67	0	0	0	0	0	0	0	0
Asp119	0	0	0	0	0	0	0	0
Arg228	1	0	0	0	0	0	0	0
Asn233	0	0	0	0	0	0	0	0

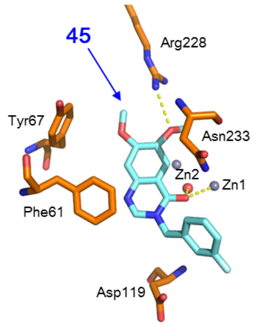

IFPscore = 0.2353

Residue	D	A	P	N	F	E	H	M
Zn1	0	0	0	0	0	0	0	0
Zn2	0	0	0	0	0	0	0	0
Phe61	0	0	0	0	1	0	1	0
Tyr67	0	0	0	0	0	0	0	0
Asp119	0	0	0	0	0	0	0	0
Arg228	1	0	0	0	0	0	0	0
Asn233	0	0	0	0	0	0	0	0

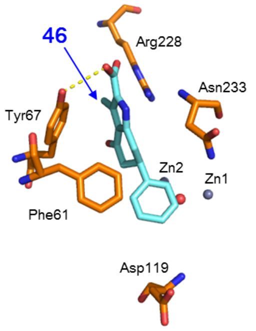

IFPscore = 0.2353

Residue	D	A	P	N	F	E	H	M
Zn1	0	0	0	0	0	0	0	0
Zn2	0	0	0	0	0	0	0	0
Phe61	0	0	0	0	0	0	1	0
Tyr67	1	0	0	0	0	0	0	0
Asp119	0	0	0	0	0	0	0	0
Arg228	0	0	1	0	0	0	0	0
Asn233	0	0	0	0	0	0	0	0

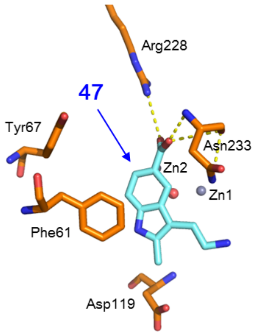

IFPscore = 0.3529

Residue	D	A	P	N	F	E	H	M
Zn1	0	0	0	0	0	0	0	0
Zn2	0	0	0	0	0	0	0	0
Phe61	0	0	0	0	0	0	0	0
Tyr67	0	0	0	0	0	0	1	0
Asp119	0	0	0	0	0	0	0	0
Arg228	1	0	1	0	0	0	0	0
Asn233	1	0	0	0	0	0	0	0

Fig. S6 Docking poses of compounds **17-20** (of which IFPscore <0.4) and **44-47** (with IFP scores below the acceptance cutoff as controls – see Table S4 for inhibition data) and their corresponding interaction fingerprints (IFPs) generated in the virtual screening.

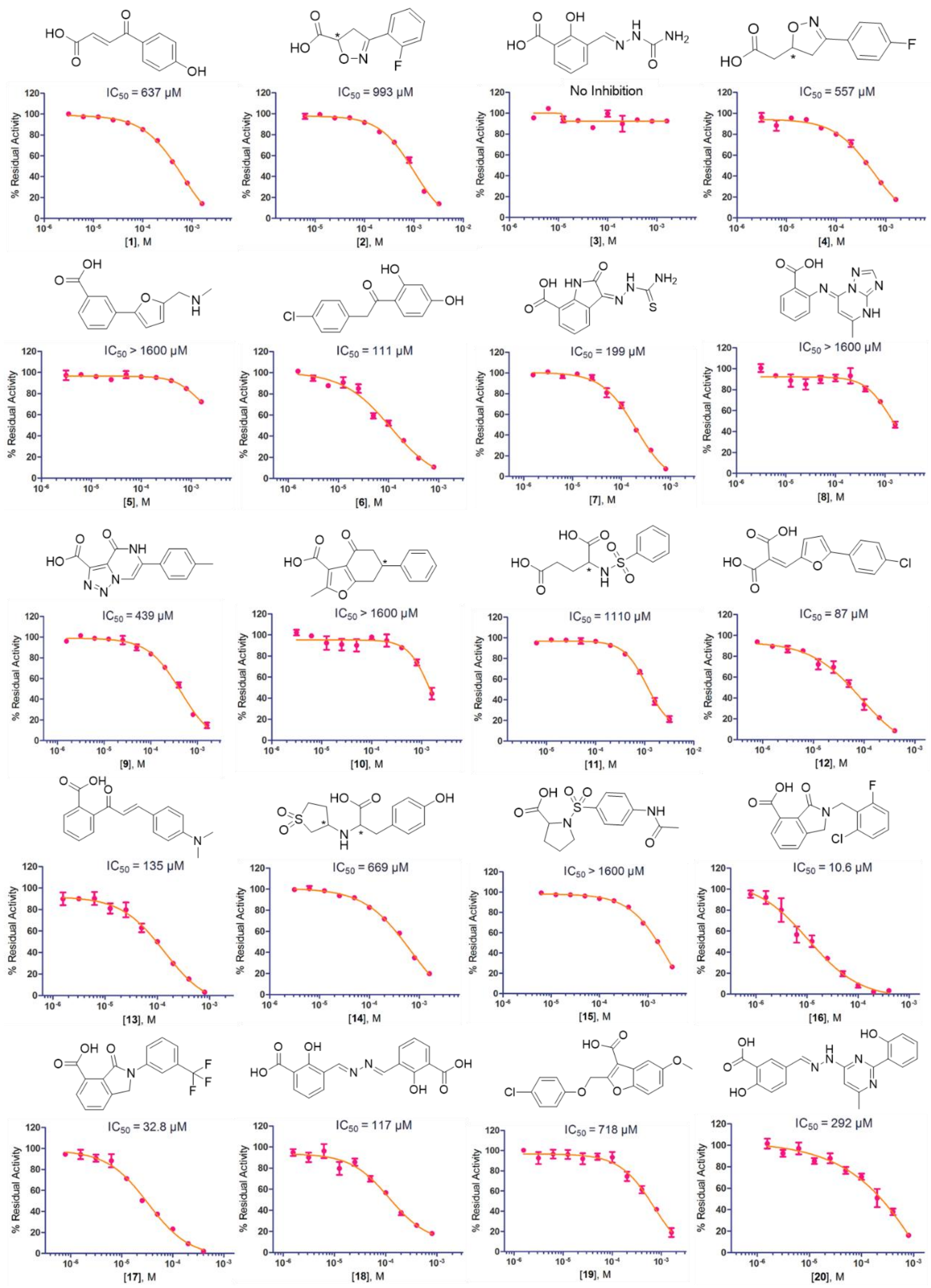

Fig. S7 IC_{50} curves of compounds **1-20** with the VIM-2 MBL.

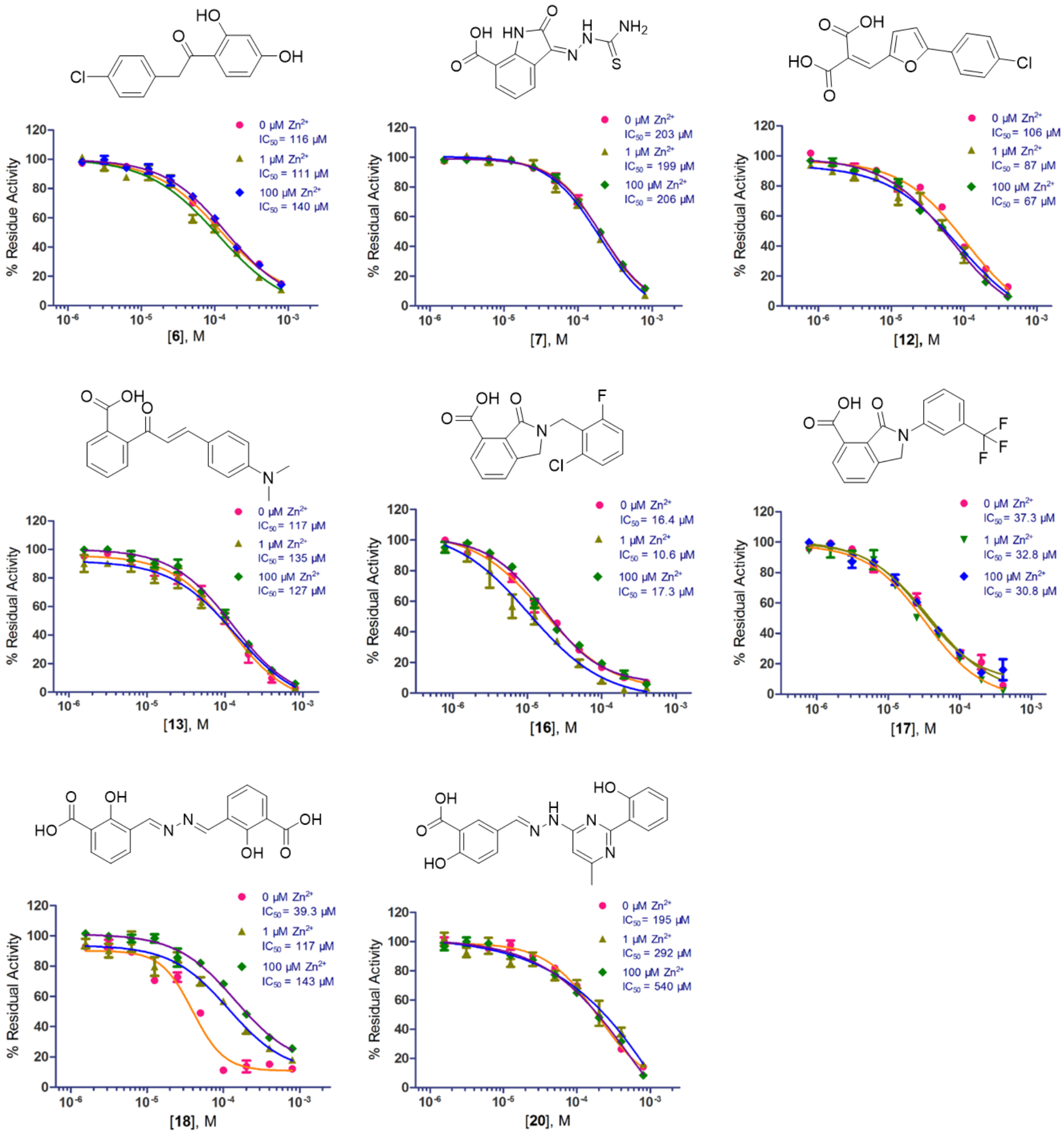

Fig. S8 IC_{50} curves of compounds **6**, **7**, **12**, **13**, **16**, **17**, **18**, and **20** with VIM-2 obtained at three different zinc ion concentrations (0 μM, 1 μM, and 100 μM).

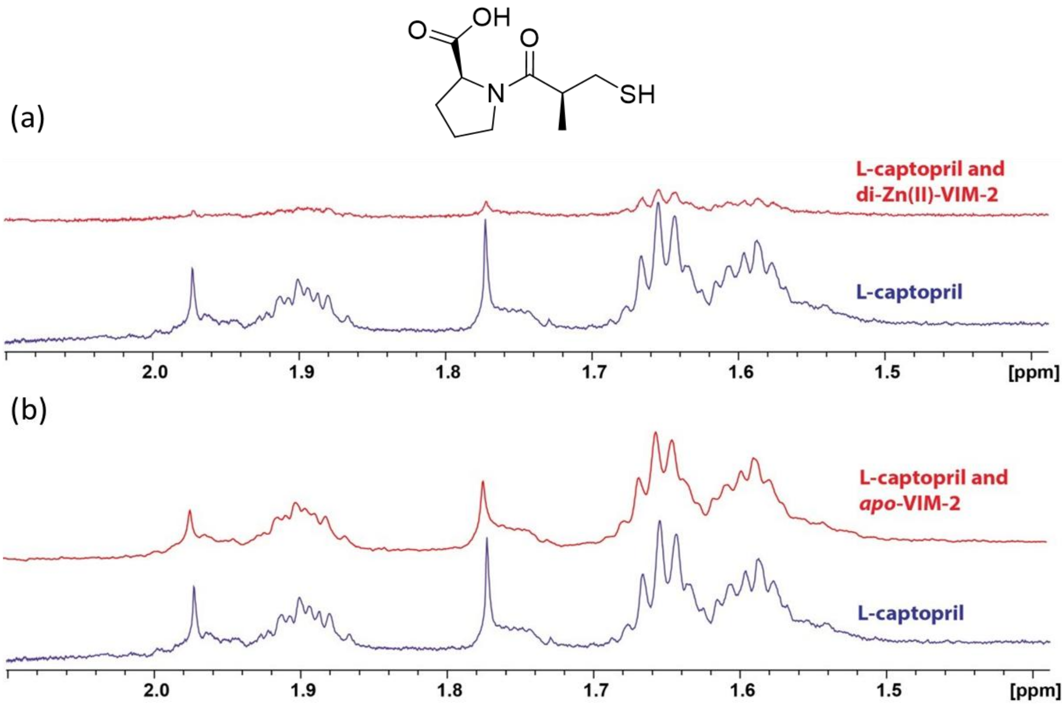

Fig. S9 NMR analyses reveal that L-captopril binds strongly to di-Zn(II)-VIM-2 but very weakly to *apo*-VIM-2. Binding studies of L-captopril to catalytically active di-Zn(II) VIM-2 by ^{1}H CPMG indicated that L-captopril has strong binding to di-Zn(II)-VIM-2 (a). Binding studies of L-captopril to *apo*-VIM-2 by ^{1}H CPMG showed that L-captopril binds very weakly to *apo*-VIM-2 (b). Assay mixtures contained 50 μM enzyme (either 50 μM di-Zn(II)-VIM-2 and 50 μM Zn(II) or *apo*-VIM-2), and 50 μM of L-captopril buffered with 50 mM Tris-D_{11}, pH 7.5, in 90 % H_2O and 10 % D_2O. Structural studies suggest that the observed weak binding to apo-VIM-2 for inhibitors with acid groups may in part be due to electrostatic interactions with Arg228[36].

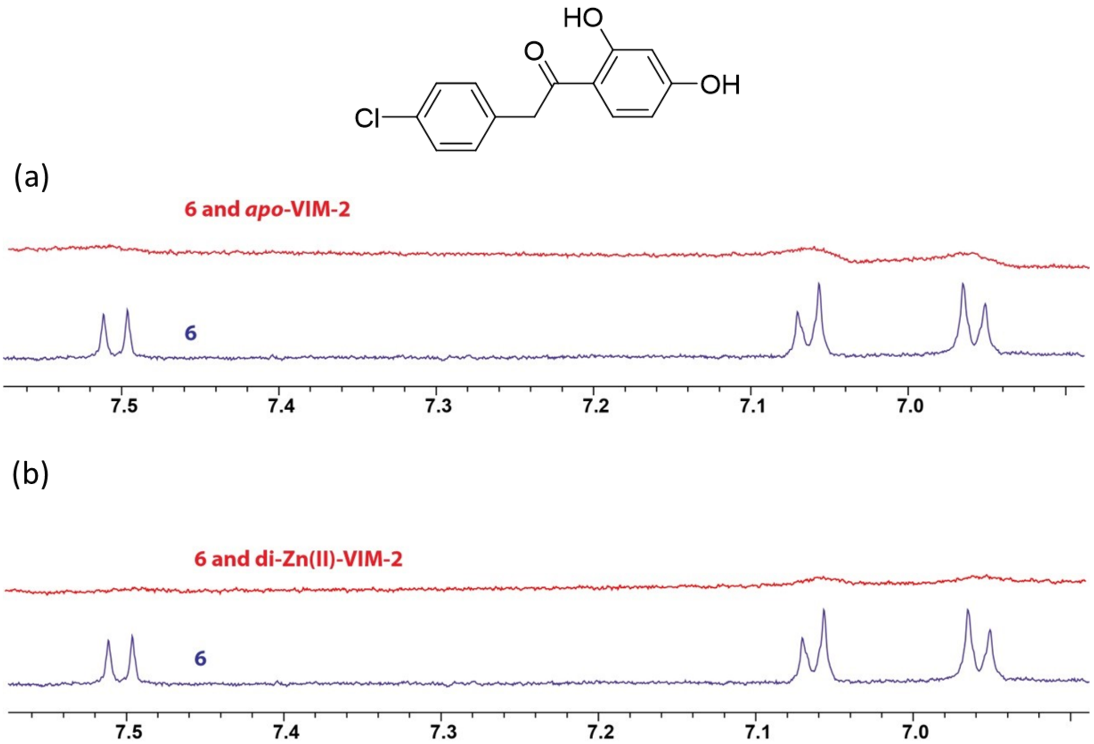

Fig. S10 NMR analyses reveal that compound 6 binds to both di-Zn(II) and *apo*-VIM-2. Binding studies of **6** to di-Zn(II) VIM-2 by ^{1}H CPMG analyses (a) indicate **6** is a strong binder to the di-Zn(II) VIM-2 MBL. (b) **6** also binds to *apo*-VIM-2. Assay mixtures contained 50 μM enzyme (either 50 μM di-Zn(II)-VIM-2 and 50 μM Zn(II) or *apo*-VIM-2), and 50 μM of **6** buffered with 50 mM Tris-D_{11}, pH 7.5, in 90 % H_2O and 10 % D_2O.

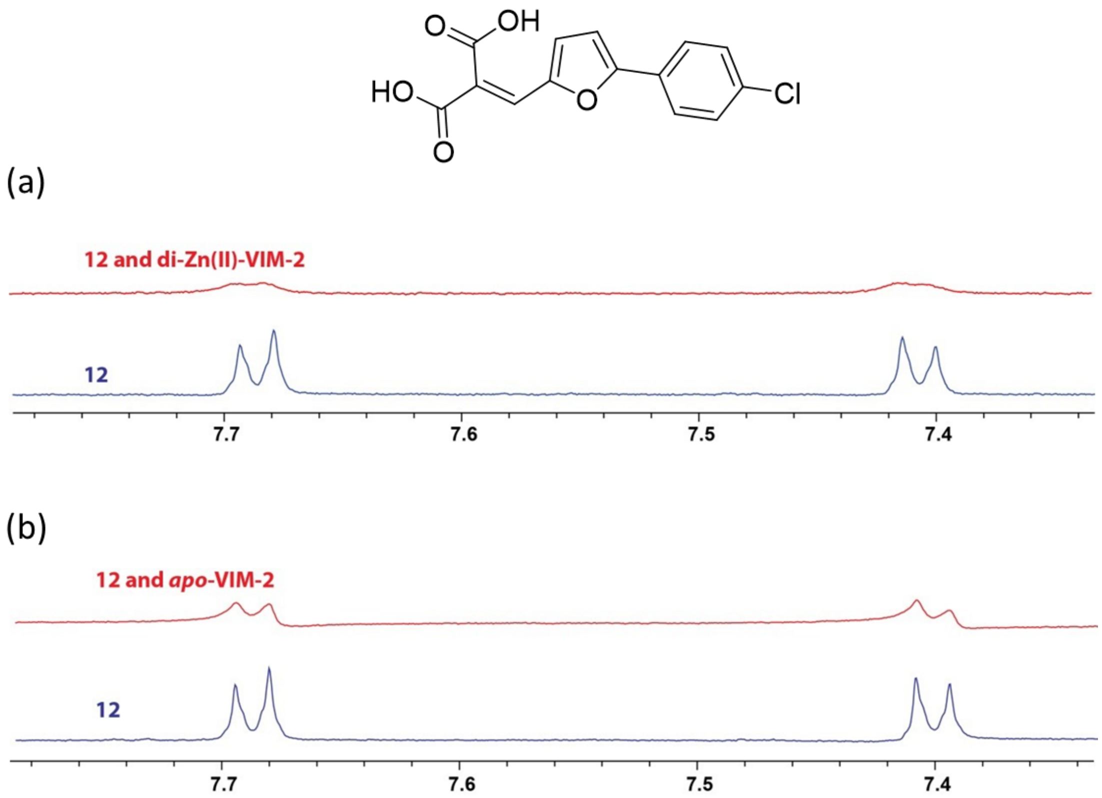

Fig. S11 NMR analyses reveal that compound 12 binds strongly to di-Zn(II)-VIM-2 but weakly to *apo*-VIM-2. Binding studies of **12** to di-Zn(II) VIM-2 (a) by ^{1}H CPMG NMR analyses indicate that **12** binds to the di-Zn(II) VIM-2 MBL. (b) **12** also binds *apo*-VIM-2. Assay mixtures contained 50 μM enzyme (either 50 μM di-Zn(II)-VIM-2 and 50 μM Zn(II) or *apo*-VIM-2), and 50 μM of **12** buffered with 50 mM Tris-D_{11}, pH 7.5, in 90 % H_2O and 10 % D_2O.

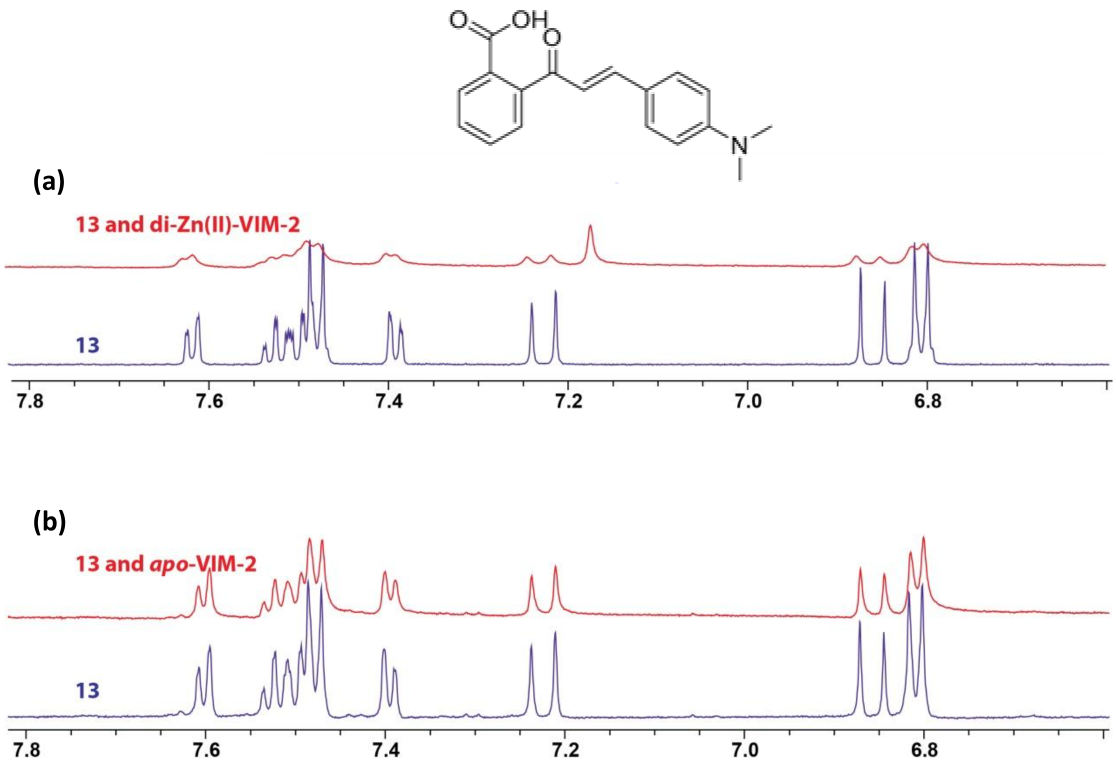

Fig. S12 NMR analyses reveal that compound 13 binds strongly to di-Zn(II)-VIM-2 proteins but weakly to *apo*-VIM-2 protein. Binding studies of **13** to catalytically active di-Zn(II) VIM-2 by ^{1}H CPMG show **13** has binds strongly to di-Zn(II)-VIM-2 (a). (b) ^{1}H CPMG analyses imply **13** binds weakly to the *apo*-VIM-2 protein. Assay mixtures contained 50 μM enzyme (either 50 μM di-Zn(II)-VIM-2 and 50 μM Zn(II) or *apo*-VIM-2), and 50 μM of **13** buffered with 50 mM Tris-D_{11}, pH 7.5, in 90 % H_2O and 10 % D_2O.

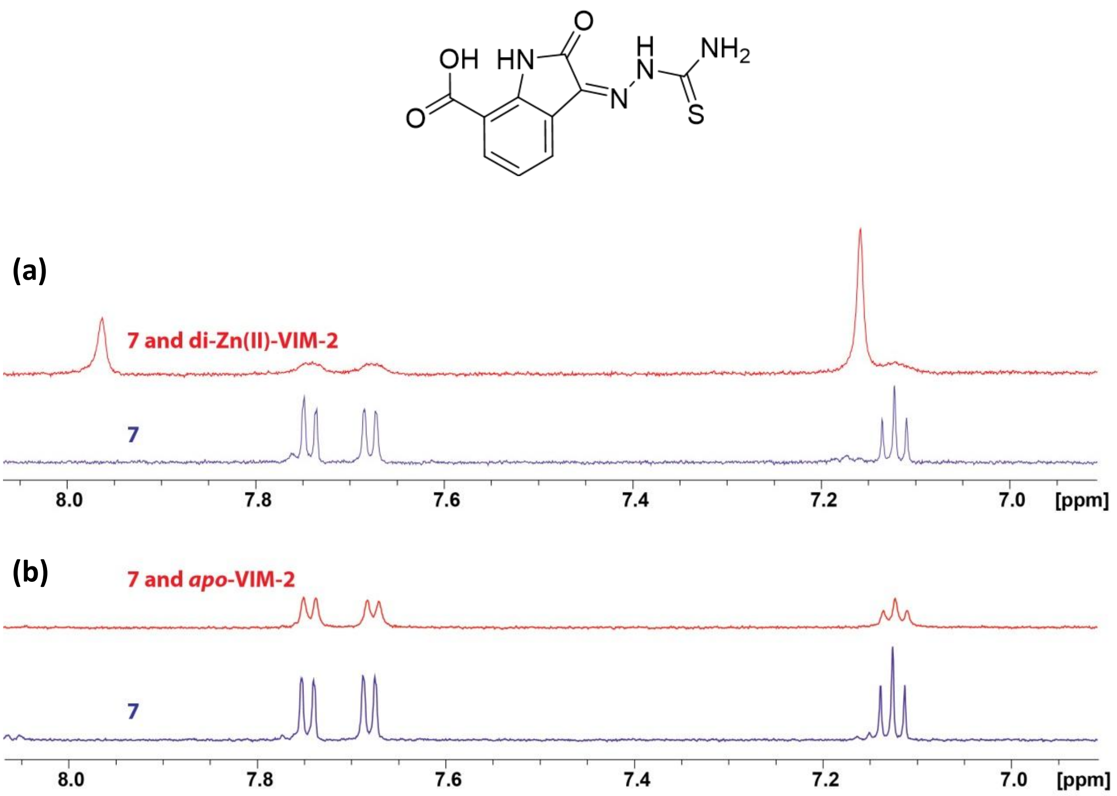

Fig. S13 NMR analyses reveal that compound 7 binds to the di-Zn(II)-VIM-2 protein. Binding studies of **7** to catalytically active di-Zn(II) VIM-2 by ^{1}H CPMG NMR analyses imply **7** binds to di-Zn(II)-VIM-2. Assay mixtures contained 50 µM di-Zn(II)-VIM-2, 50 µM Zn(II) and 50 µM of **7** buffered with 50 mM Tris-D_{11}, pH 7.5, in 90 % H_2O and 10 % D_2O. Additional peaks near 7.18 and 7.98 ppm are contaminating imidazole from protein purification step (see figure S15 below).

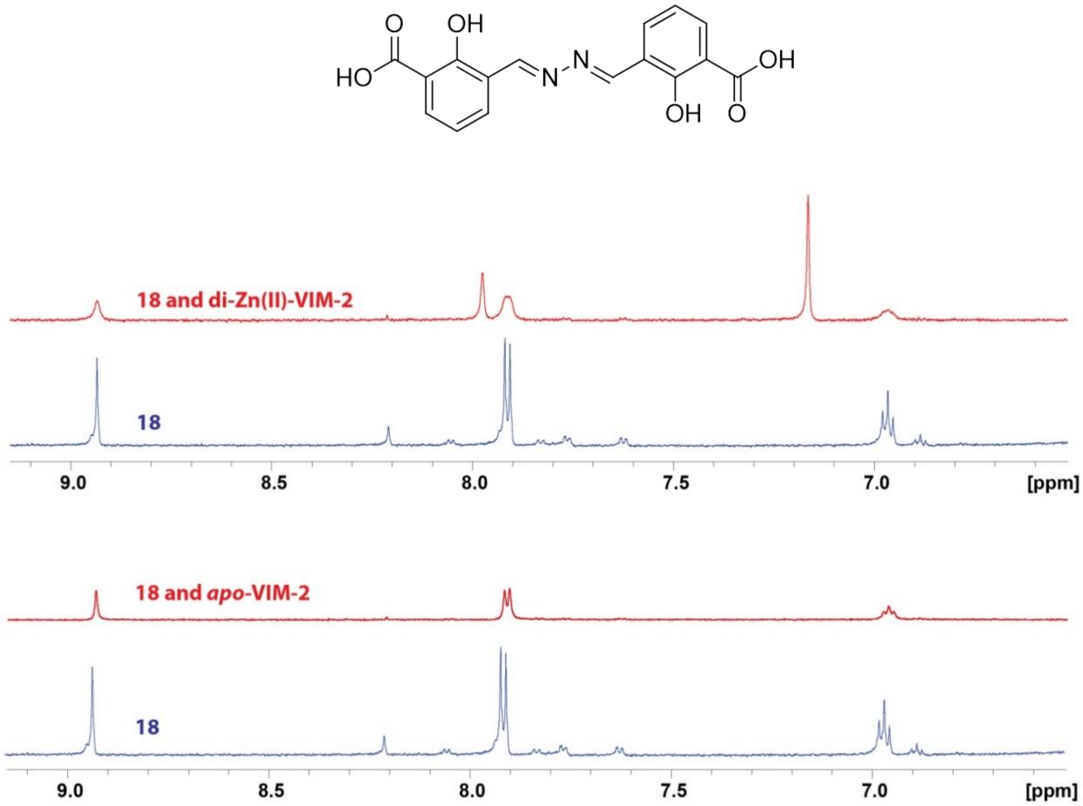

Fig. S14 NMR analyses showed that compound 18 binds to the di-Zn(II)-VIM-2 protein. Binding studies of **18** to catalytically active di-Zn(II) VIM-2 by ^{1}H CPMG NMR analyses demonstrates that **18** binds to di-Zn(II)-VIM-2 (a). (b) **18** also binds to the *apo*-VIM-2 protein. Assay mixtures contained 50 µM enzyme (either 50 µM di-Zn(II)-VIM-2 and 50 µM Zn(II) or *apo*-VIM-2), and 50 µM of **18** buffered with 50 mM Tris-D_{11}, pH 7.5, in 90 % H_2O and 10 % D_2O. Additional peaks near 7.18 and 7.98 ppm are contaminating imidazole from protein purification step (see figure S15 below).

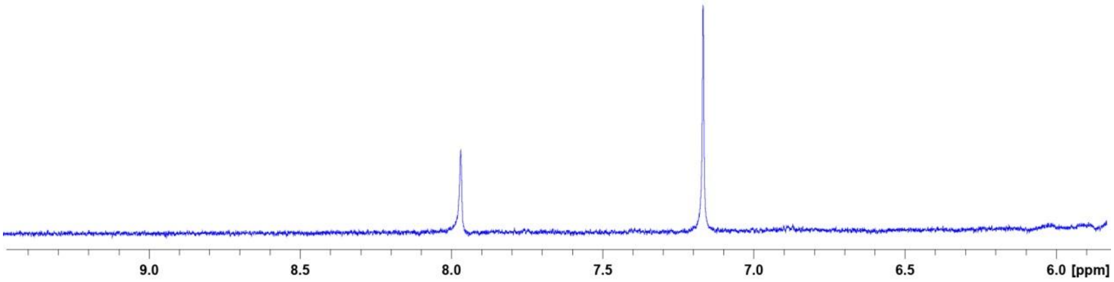

Fig. S15 ^{1}H CPMG NMR spectrum of 50 µM di-Zn(II)-VIM-2 and 50 µM Zn(II) buffered with 50 mM Tris-D_{11}, pH 7.5, in 90 % H_2O and 10 % D_2O, showing the presence of imidazole from the affinity purification.

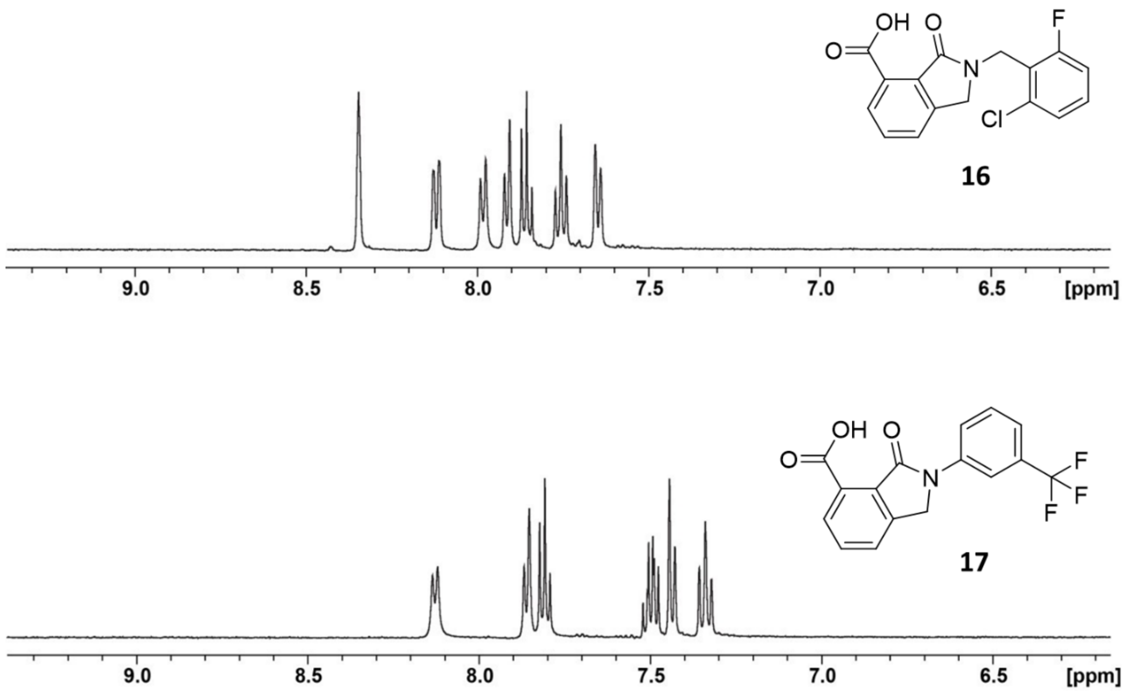

Fig. S16 ^{1}H NMR spectra of 2 mM compounds **16** and **17** in DMSO-D_6, which are different from those in 50 mM Tris-D_{11}, pH 7.5, in 90 % H_2O and 10 % D_2O.

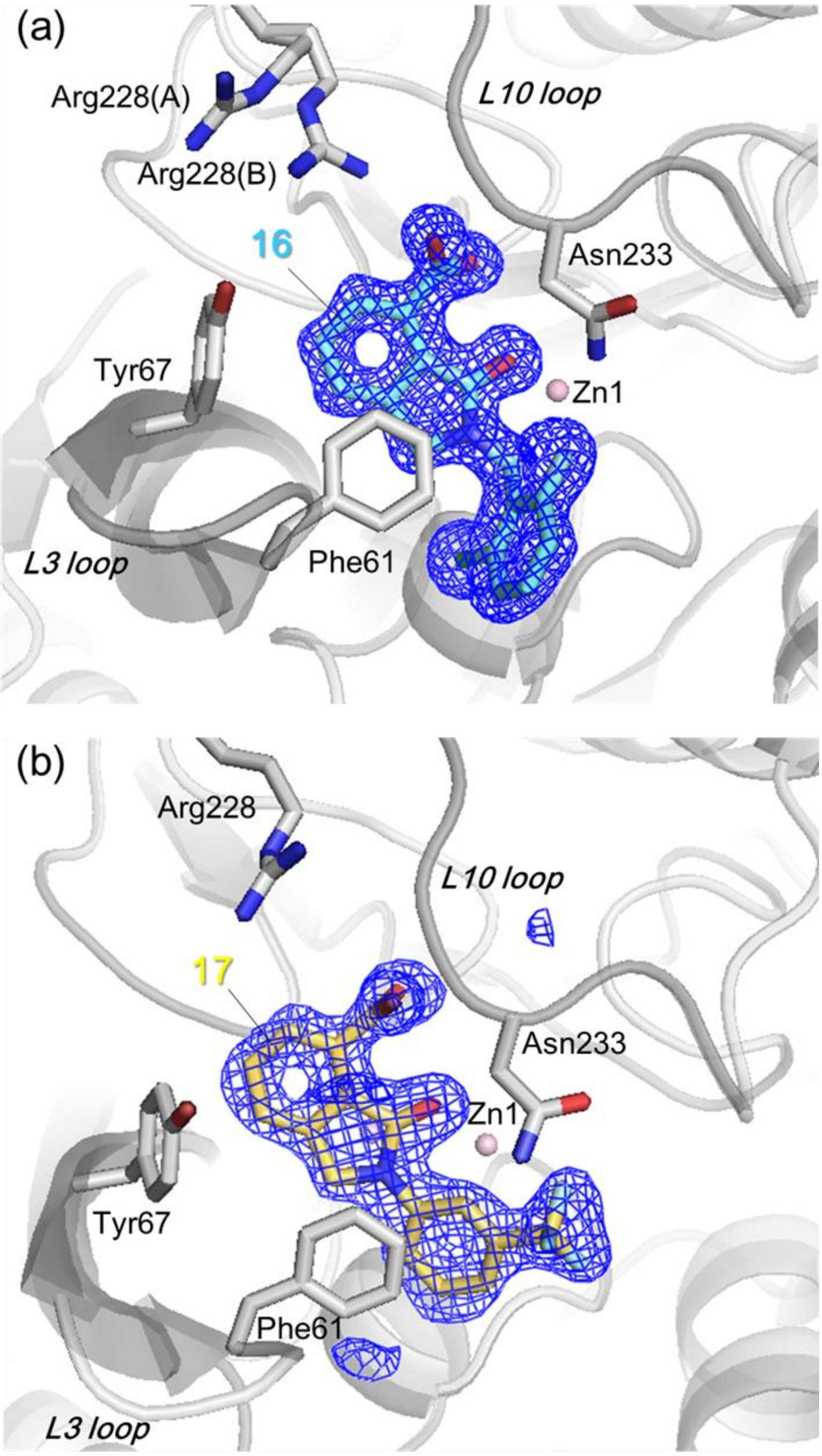

Fig. S17 Modes of 16 and 17 binding as defined by electron density maps. Complex structures of (a) VIM-2:**16** (PDB ID: 5LE1) and (b) VIM-2:**17** (PDB ID: 5LCA) (protein and compound colors and representations as in Fig. 2) with the mF_o-DF_c electron density (OMIT maps) around **16** and **17** (blue mesh, contoured to 3σ) calculated from the final refined model.

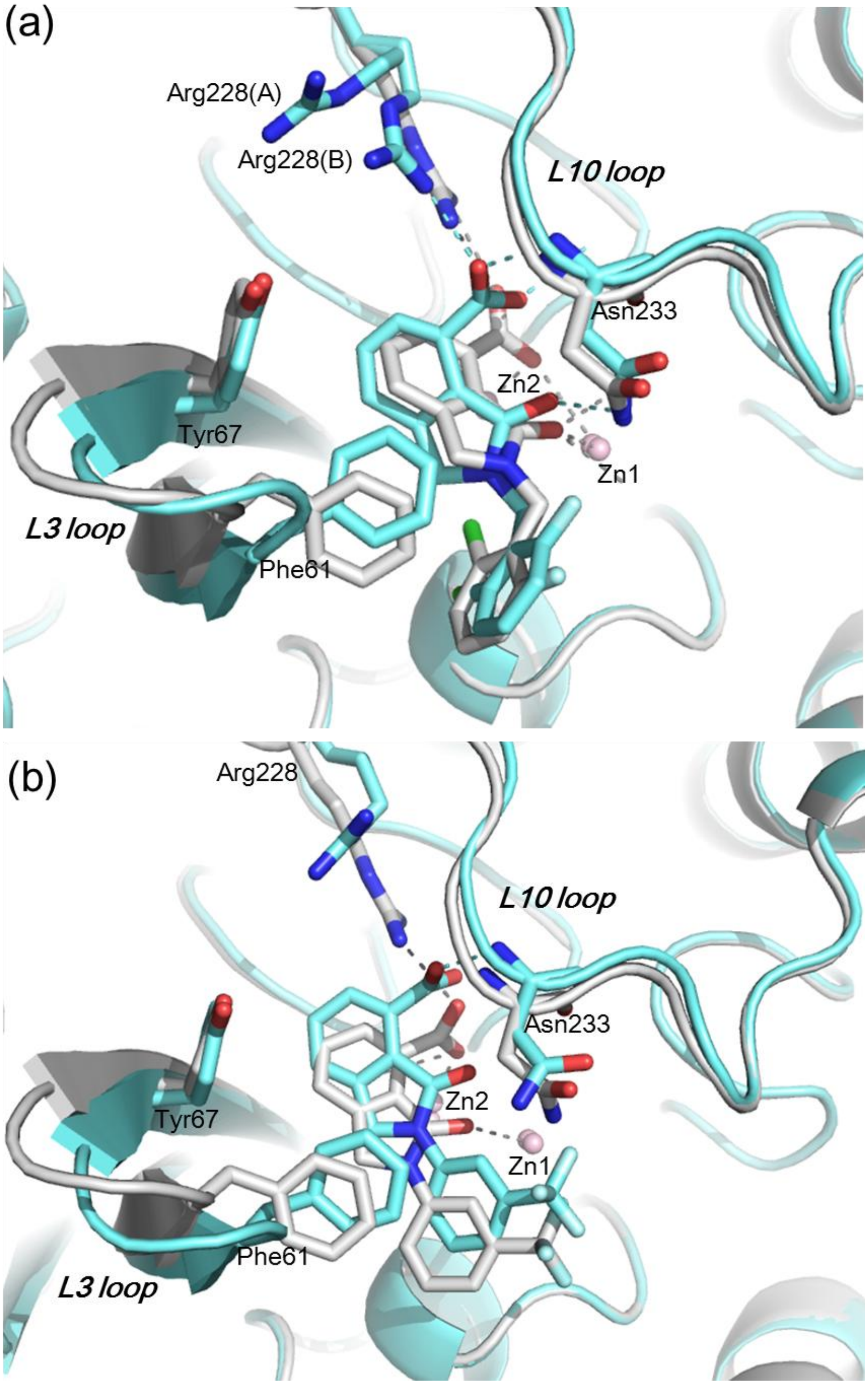

Fig. S18 Comparison of the predicted binding modes of compounds 16 and 17 with their crystal structures. (a) Superimposition of a docking pose of **16** (white) with a crystal structure of the VIM-2:**16** complex (cyan, PDB ID: 5LE1). (b) Superimposition of the docking pose of **17** (white) and a crystal structure of the VIM-2:**17** complex (cyan, PDB ID: 5LCA). The predicted binding modes of **16** and **17** are quite similar to those observed in their crystal structures, including with respect to the hydrogen-bonding interactions formed with Arg228 and π-π stacking interactions with Phe61 (Fig. S5 and S6). The RMSD values (all atoms) between **16** and **17** docking poses with the observed crystal structures are 1.8 and 1.6 Å, respectively.

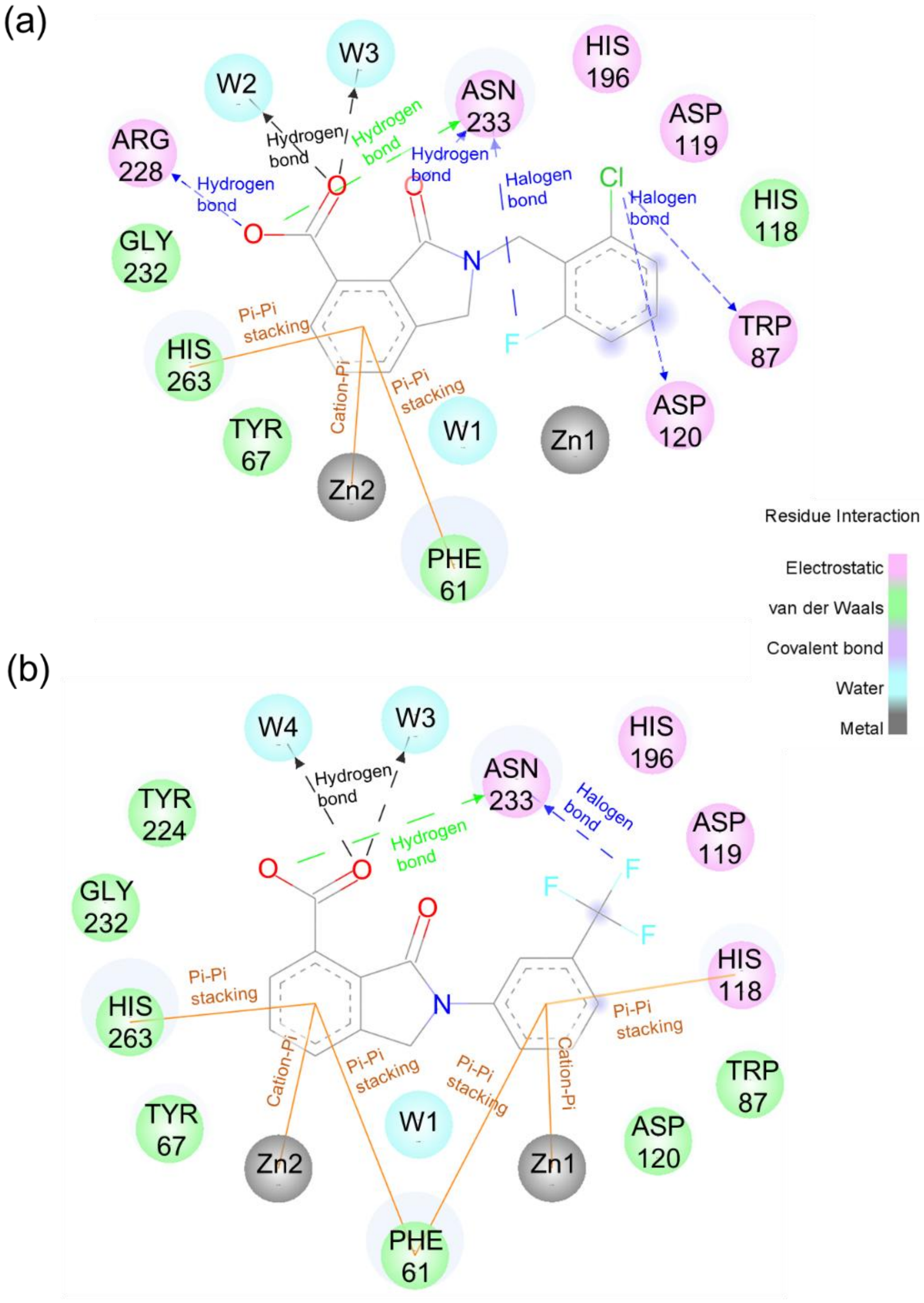

Fig. S19 Protein-ligand interactions between 16 and 17 and VIM-2 defined using the Discovery Studio Visualizer. Compounds **16** and **17** display very similar interactions with VIM-2 active site residues and zinc ions, including with respect to the 3-oxoisoindoline-4-carboxylate being positioned to form π-π stacking interactions with Phe61 and His263, hydrogen-bonding interactions with Asn233 and the 'structural molecule water' W3, and the phenyl ring forming a *cation*-π interaction with Zn2.

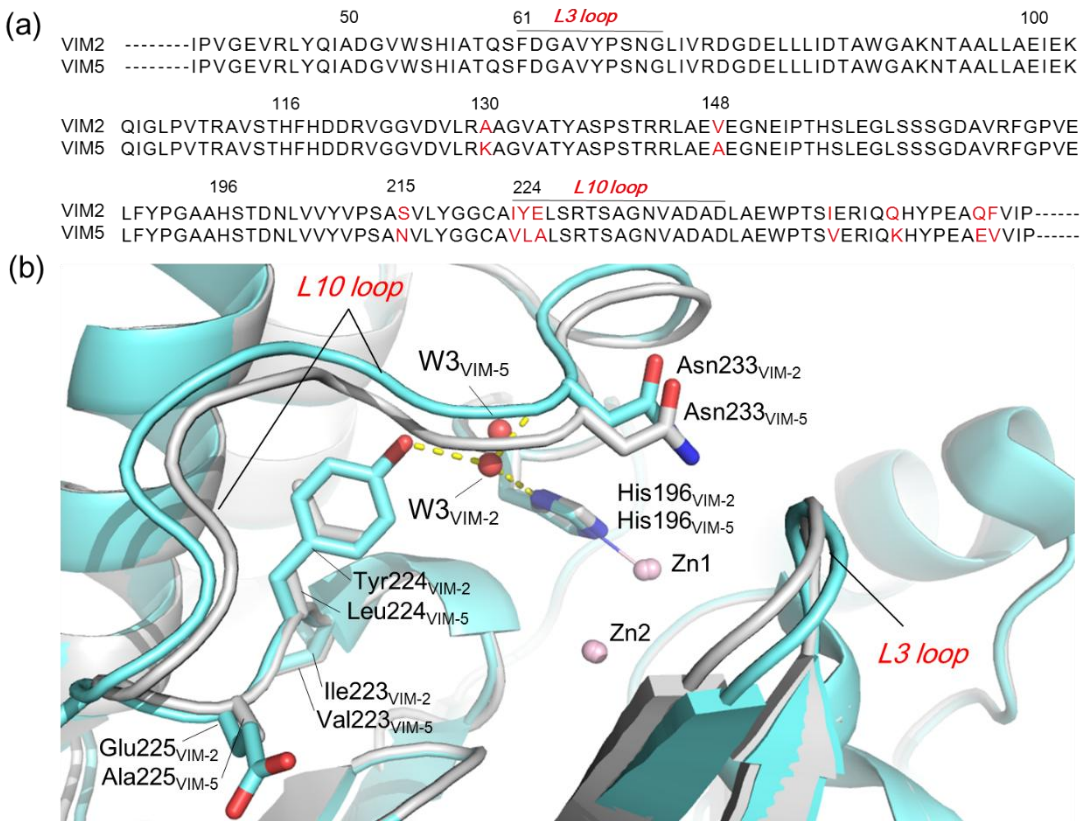

Fig. S20 Comparison of VIM-2 and VIM-5 MBLs. (a) Sequence alignments of VIM-2 and VIM-5 show high similarity[11, 37]. (b) As observed by crystallography, the active site features of VIM-2 (PDB: 4BZ3) and VIM-5 (PDB: 5A87) are very similar with only three different residues (Ile223$_{VIM-2}$/Val223$_{VIM-5}$, Tyr224$_{VIM-2}$/Leu224$_{VIM-5}$, and Glu225$_{VIM-2}$/Ala225$_{VIM-5}$) and a small difference in their L10 loops.

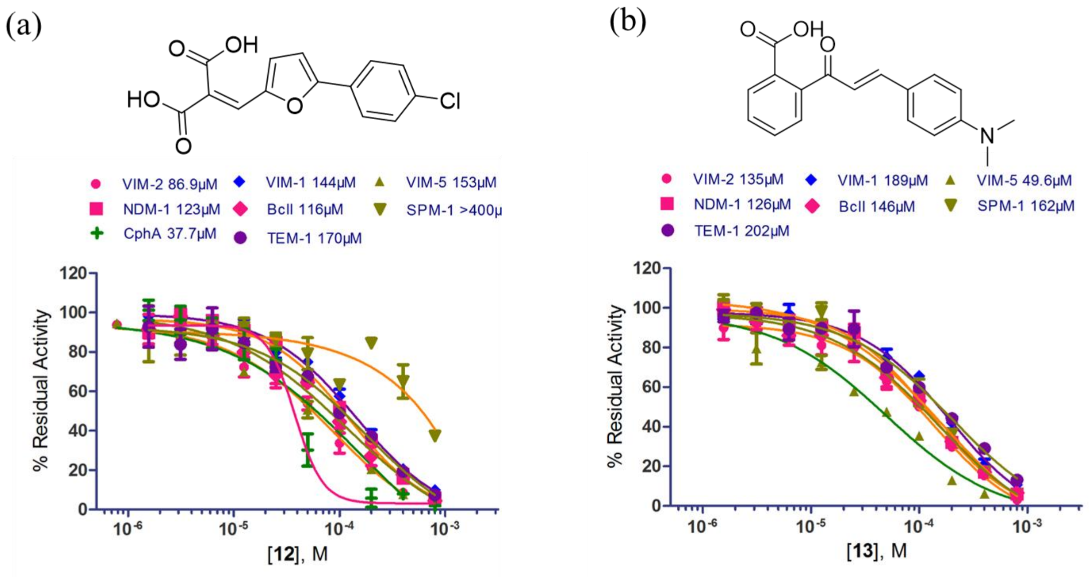

Fig. S21 Selectivity profile of compounds 12 and 13 with the shown β-lactamases. Detailed assay conditions please see Supplementary Experimental Section SE. 3 and Table S1.

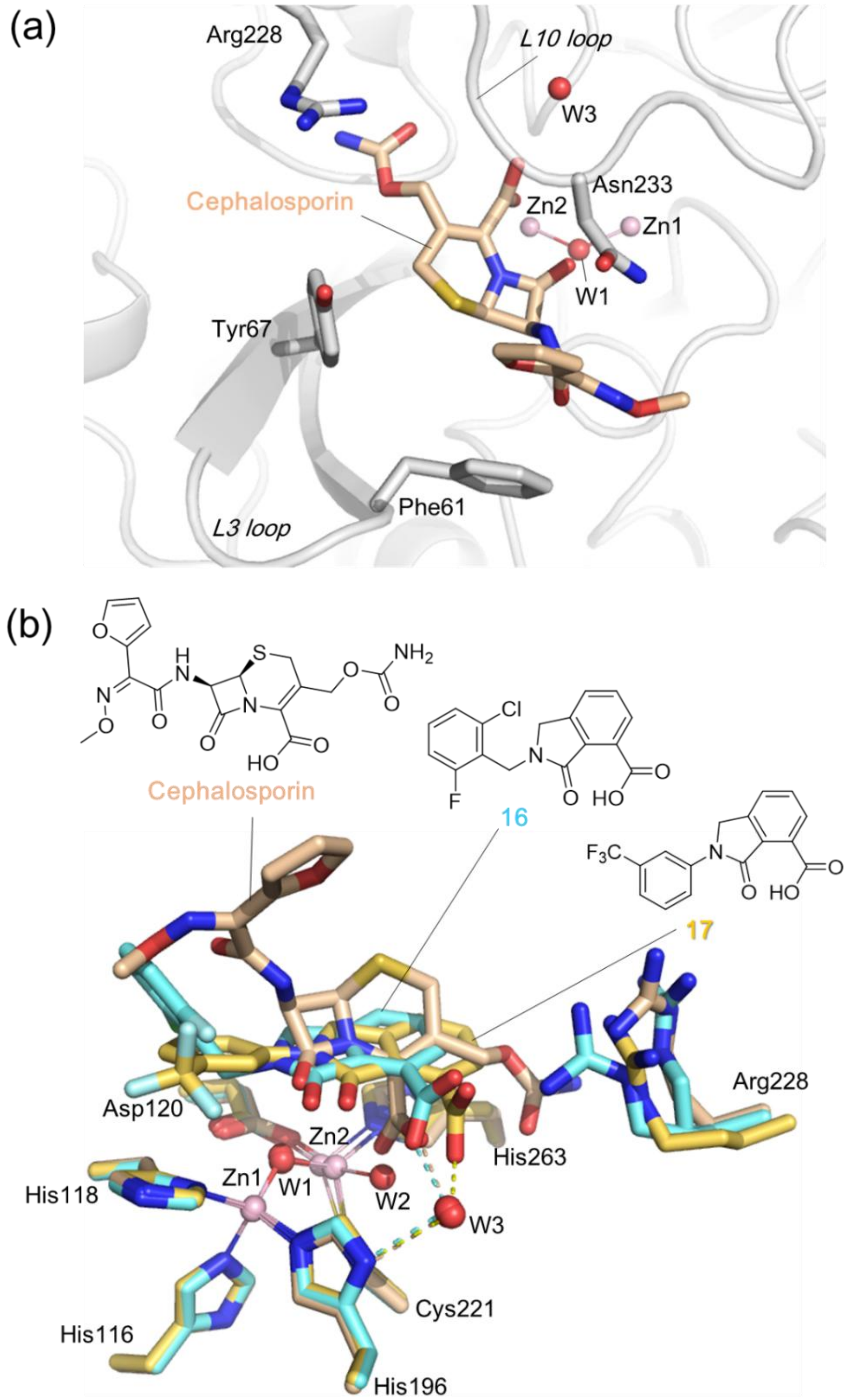

Fig. S22 Comparison of a proposed binding mode for an intact cephalosporin with the observed binding modes of 16 and 17. (a) The predicted docking pose of a cephalosporin (Cefuroxime) with VIM-2 (PDB ID: 4BZ3). The docked cephalosporin is in position to form hydrogen-bond interactions with Asn233 and the water molecule W3, as well as hydrophobic interactions with Phe61 and Tyr67. (b) Superimposition of the predicted docking pose of the cephalosporin with the crystal structures of VIM-2:**16** (PDB ID: 5LE1) and VIM-2:**17** (PDB ID: 5LCA) reveals that **16** and **17**, in particular their heterocyclic core, bind similarly to the cephalosporin.

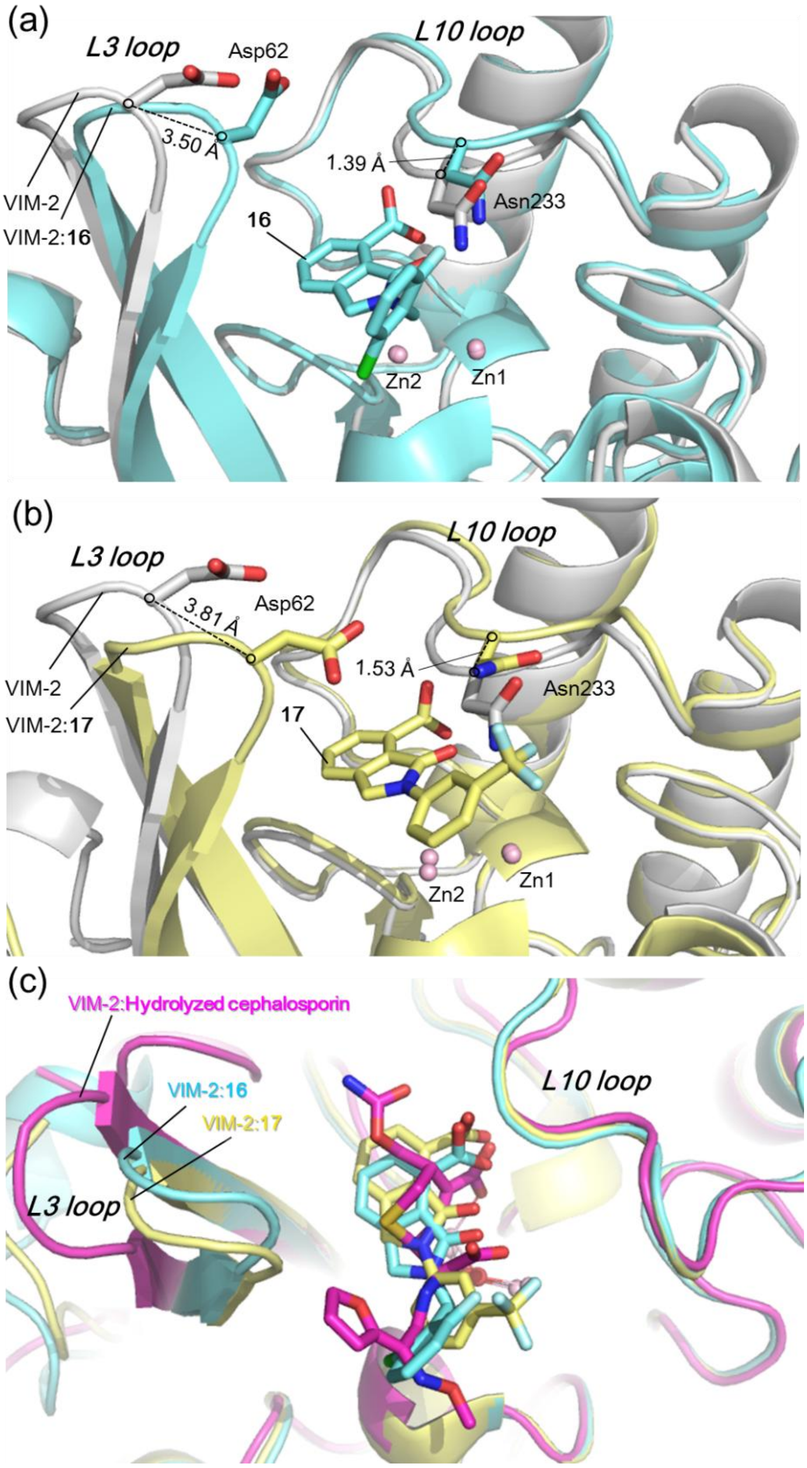

Fig. S23 VIM-2 loop movements. Comparison of VIM-2 (white, PDB ID: 4BZ3) with (a) VIM-2:**16** (aquamarine, PDB ID: 5LE1) and (b) VIM-2:**17** (yellow, PDB ID: 5LCA) shows the L3 loop appears to clamp down on the active site upon **16** and **17** binding, whilst the L10 loop appears to move slightly away from the active site. (c) Superimposing structures of VIM-2:**16** and VIM-2:**17** with that of a representative ring-opened cephalosporin 'intermediate' in complex with NDM-1 (magenta, PDB ID: 4RL0)[38], reveals that **16** and **17** have a similar binding mode with the cephalosporin, including interactions around the L3 and L10 loops.

(a)

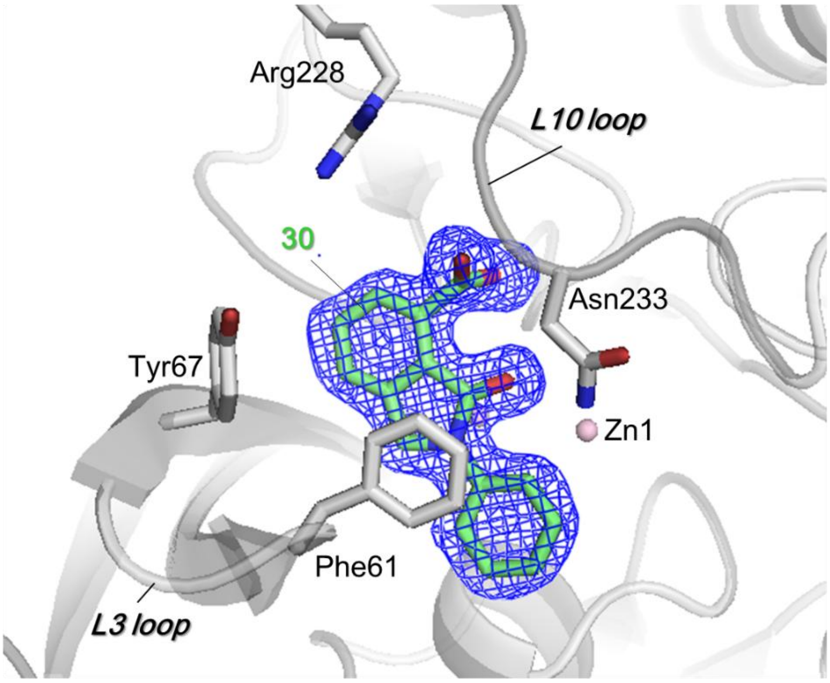

(b)

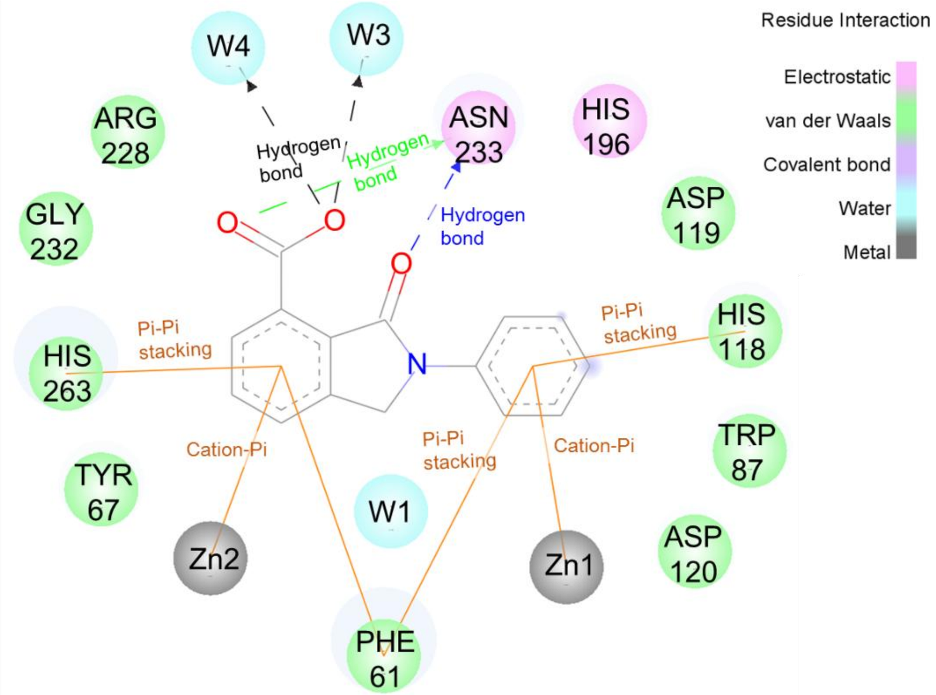

Fig. S24 Mode of compound 30 binding as defined by electron density maps. (a) A structure of the VIM-2:**30** complex (PDB ID: 5LCF) with the mF_o-DF_c electron density (OMIT map) around **30** (blue mesh, contoured to 3σ) calculated from the final refined model (protein and compound colors and representations as in Fig. 5a). (b) Protein-ligand interactions between VIM-2 and **30** as described using the Discovery Studio Visualizer.

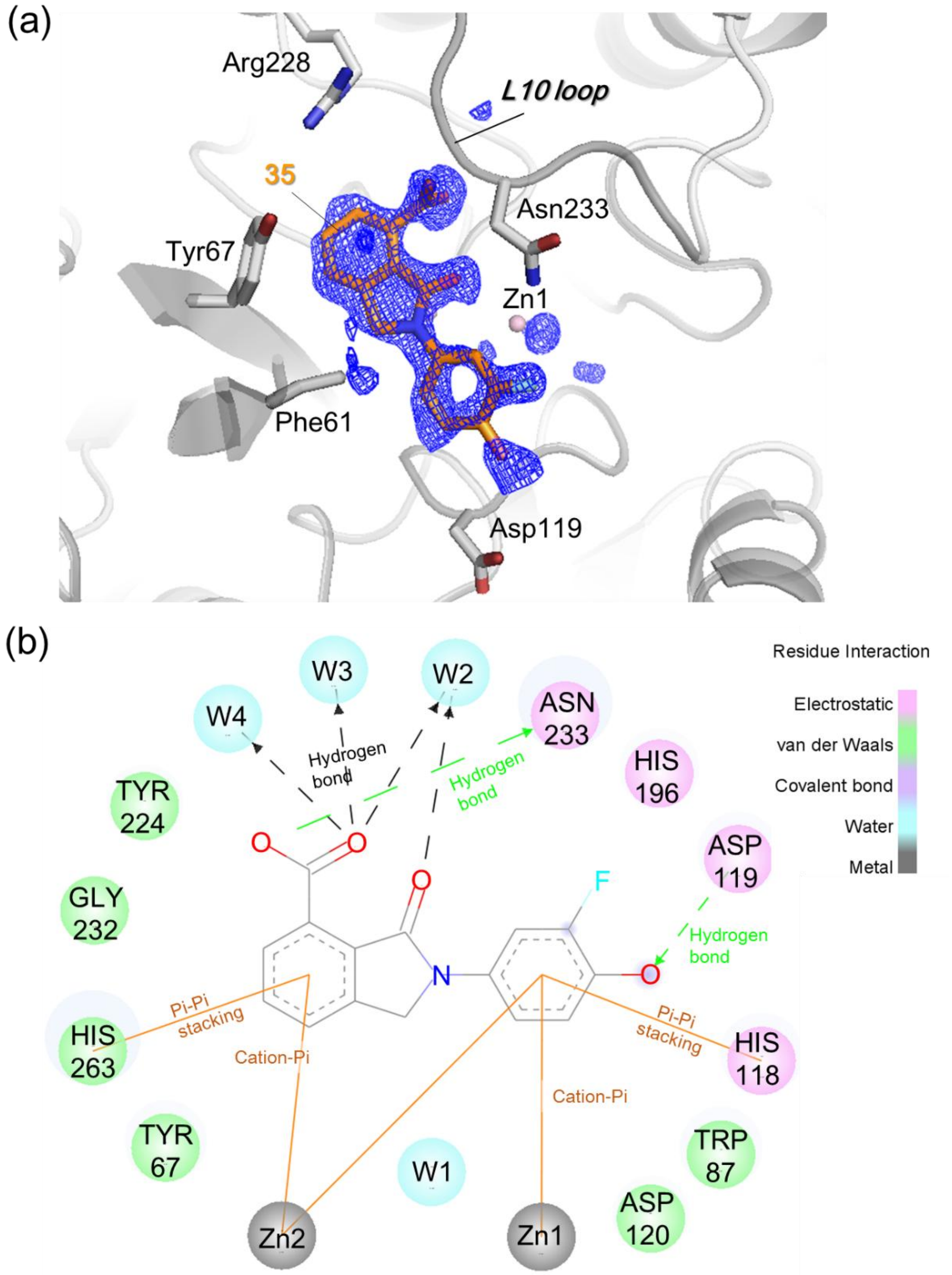

Fig. S25 Mode of compound 35 binding as defined by electron density maps. (a) A structure of VIM-2:**35** complex (PDB ID: 5LM6) with the mF_o-DF_c density (blue mesh, contoured to 3σ) calculated from the final refinement model (protein and compound colors and representations as in Fig. 5c). Although the density for the Phe61 side chain is not observed in the VIM-2:**35** complex structure, **35** appears to fit well with the density maps; note **30** and **35** have the same binding mode as observed by crystallography (Fig. 5d). (b) Protein-ligand interactions between VIM-2 and **35** as described using the Discovery Studio Visualizer.

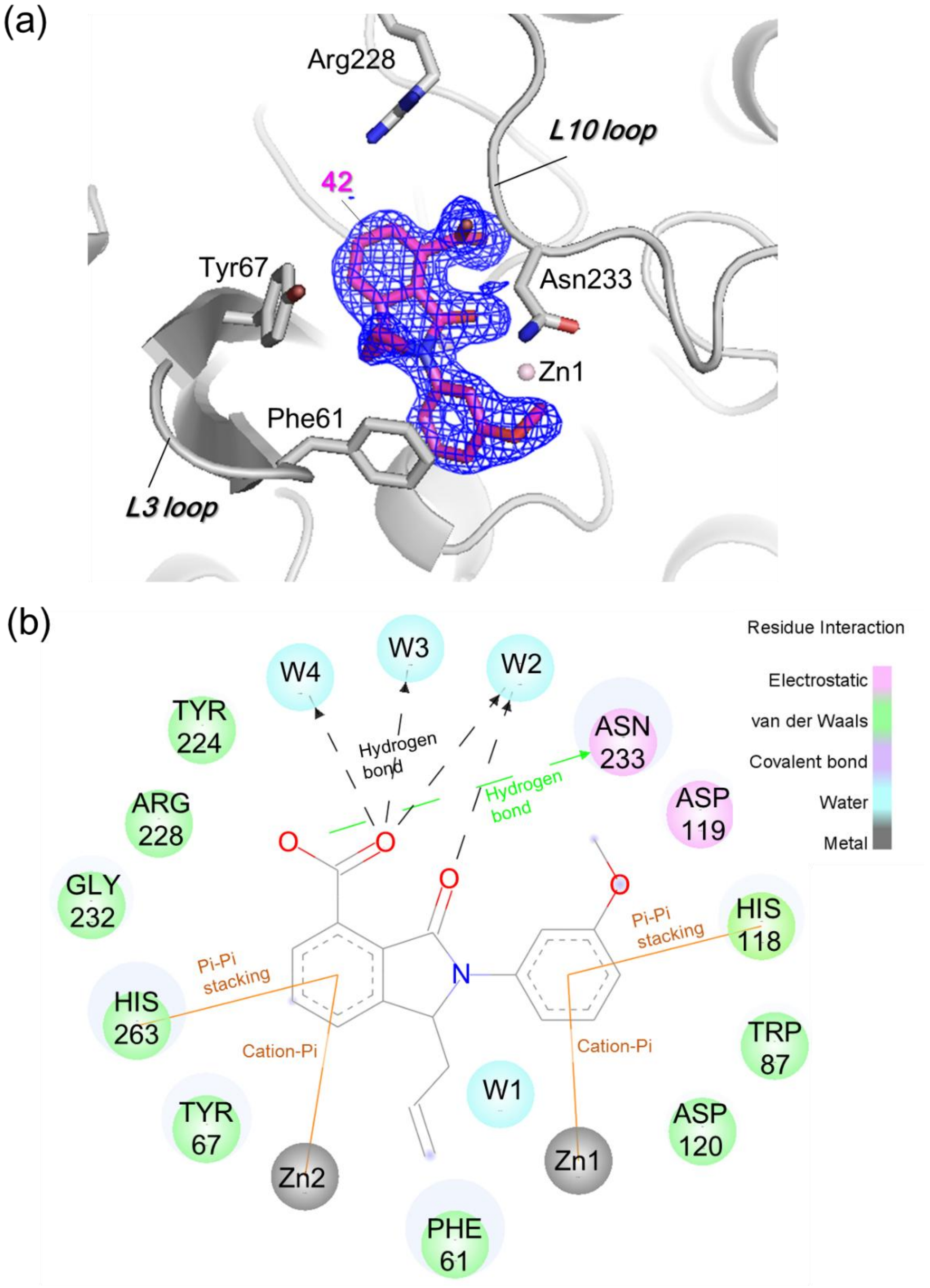

Fig. S26 Mode of compound 42 binding as defined by electron density maps. (a) A structure of VIM-2:**42** complex (PDB ID: 5LCH) with the mF_o-DF_c density (blue mesh, contoured to 3σ) calculated from the final refinement model (protein and compound colors and representations as in Fig. 5e). (b) Protein-ligand interactions between VIM-2 and **42** as described using the Discovery Studio Visualizer.

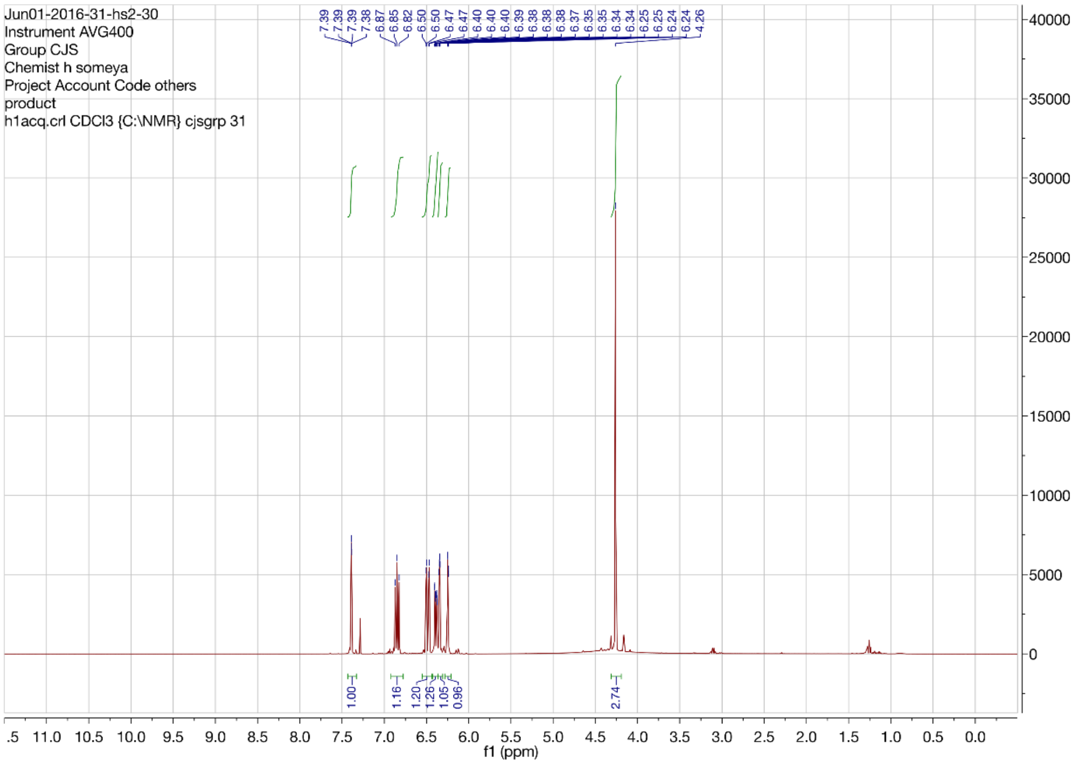

Fig. S27 ^{1}H NMR spectrum of **35a**.

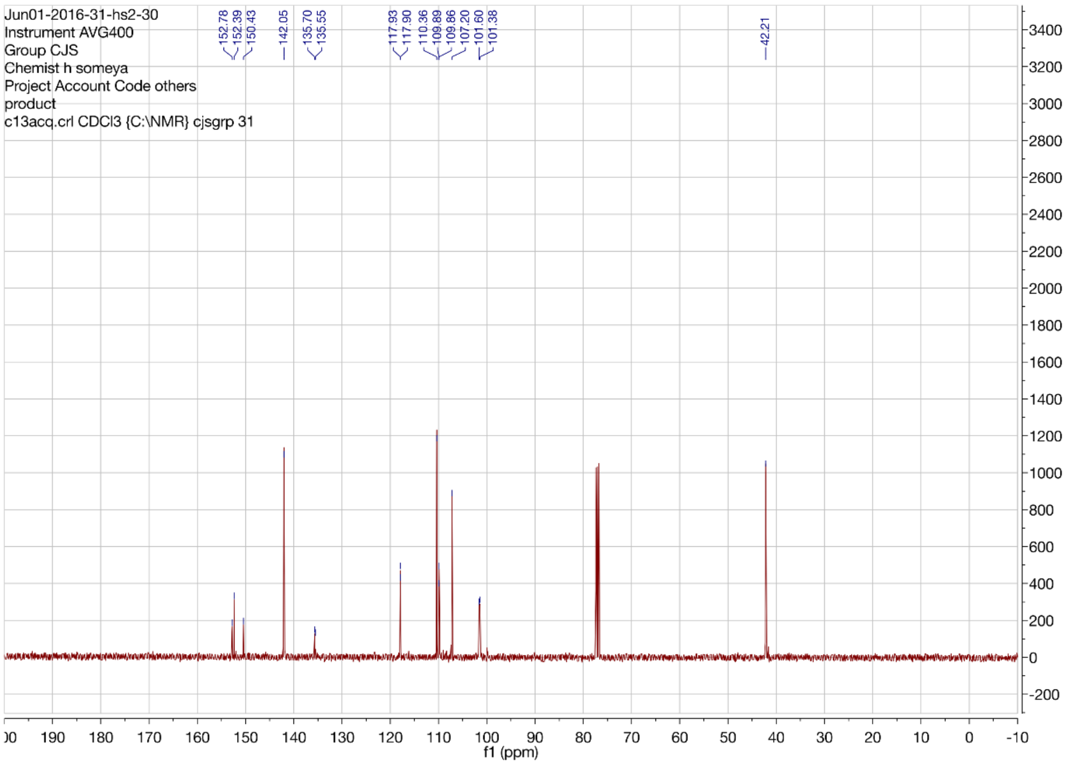

Fig. S28 ^{13}C NMR spectrum of **35a**.

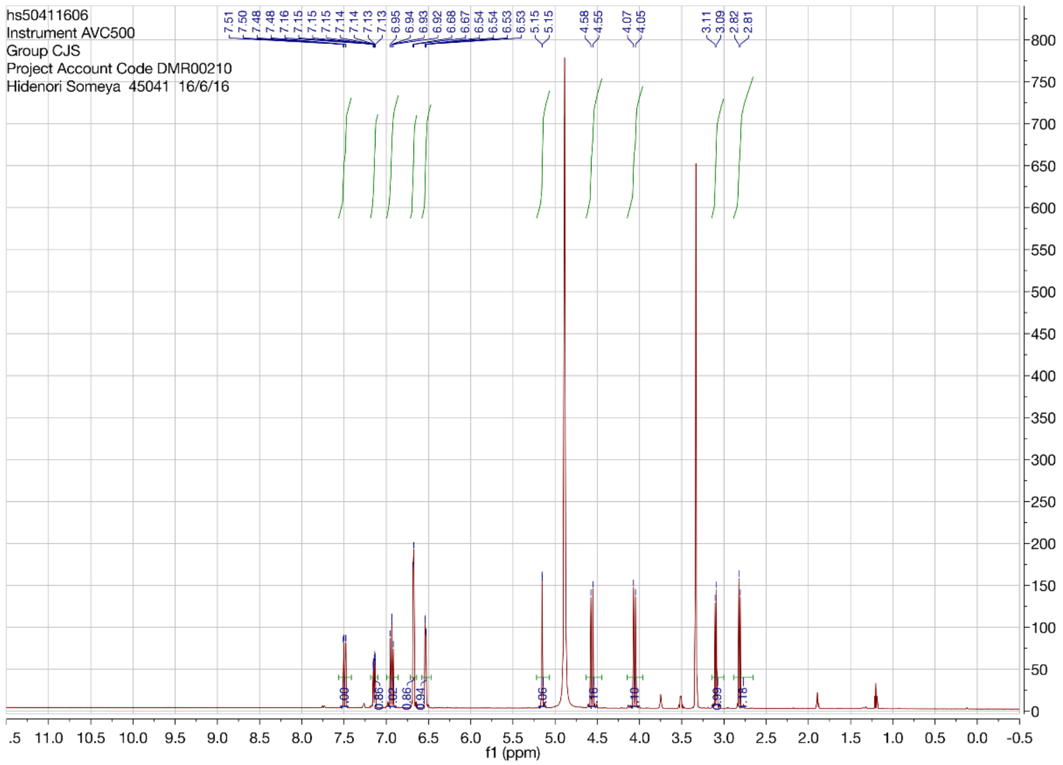

Fig. S29 ^{1}H NMR spectrum of **35b**.

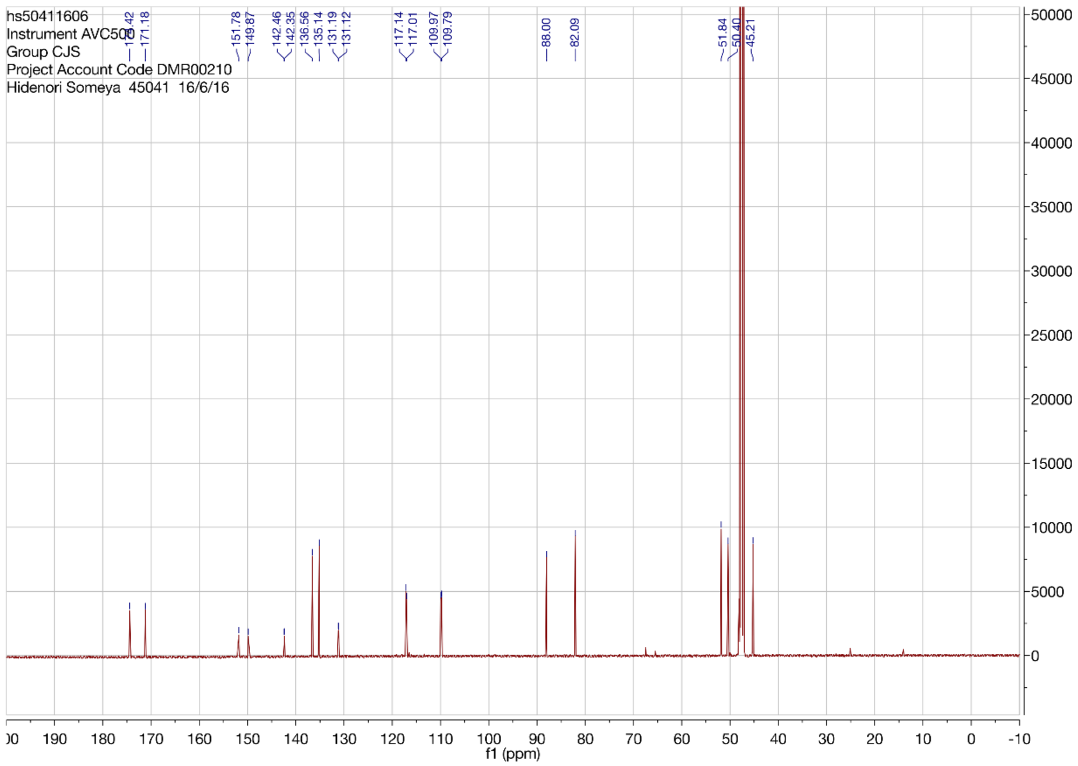

Fig. S30 ^{13}C NMR spectrum of **35b**.

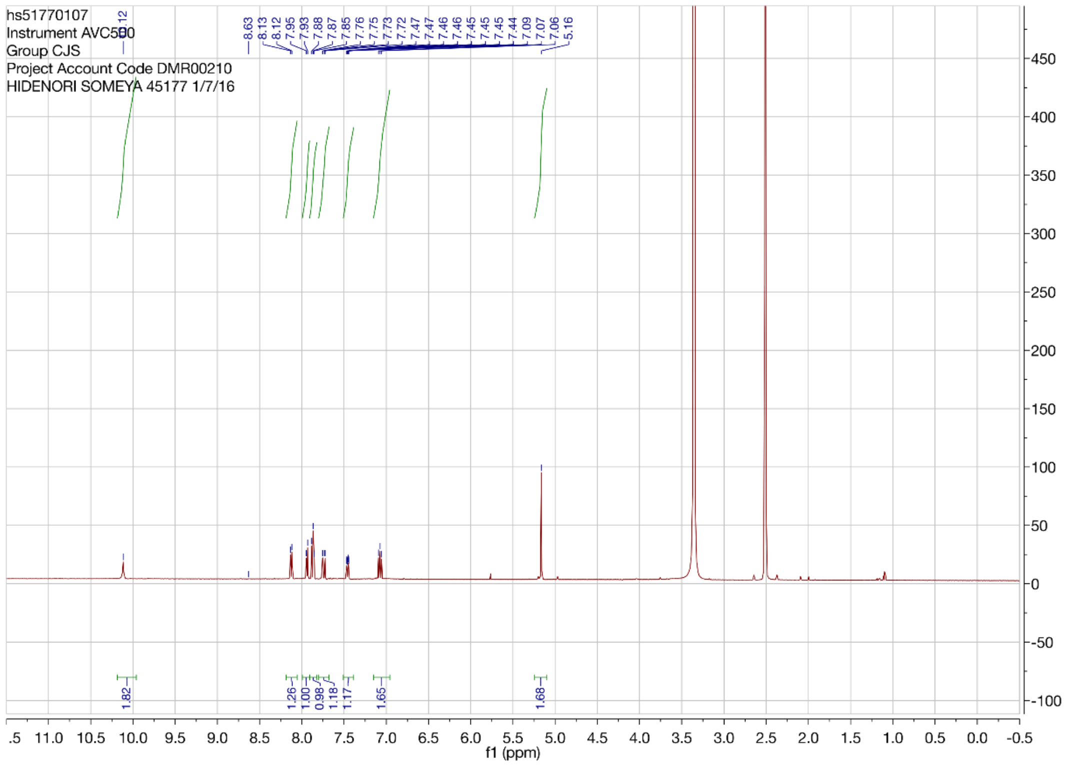

Fig. S31 ^{1}H NMR spectrum of **35**.

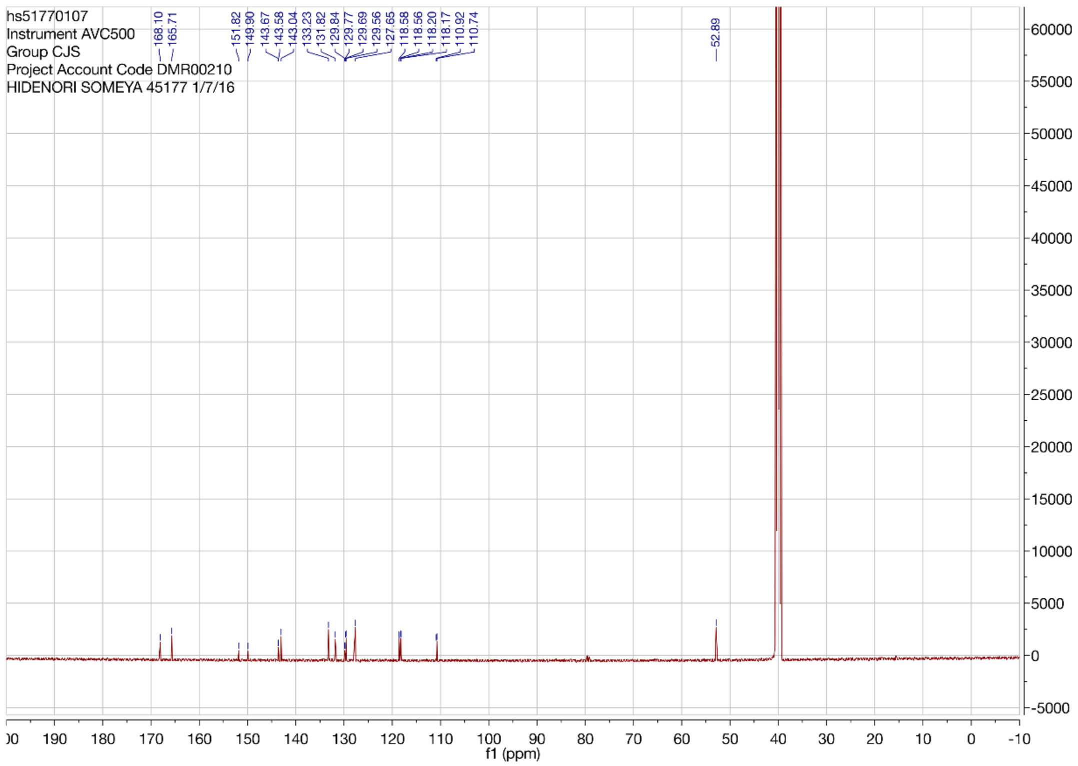

Fig. S32 ^{13}C NMR spectrum of **35**.

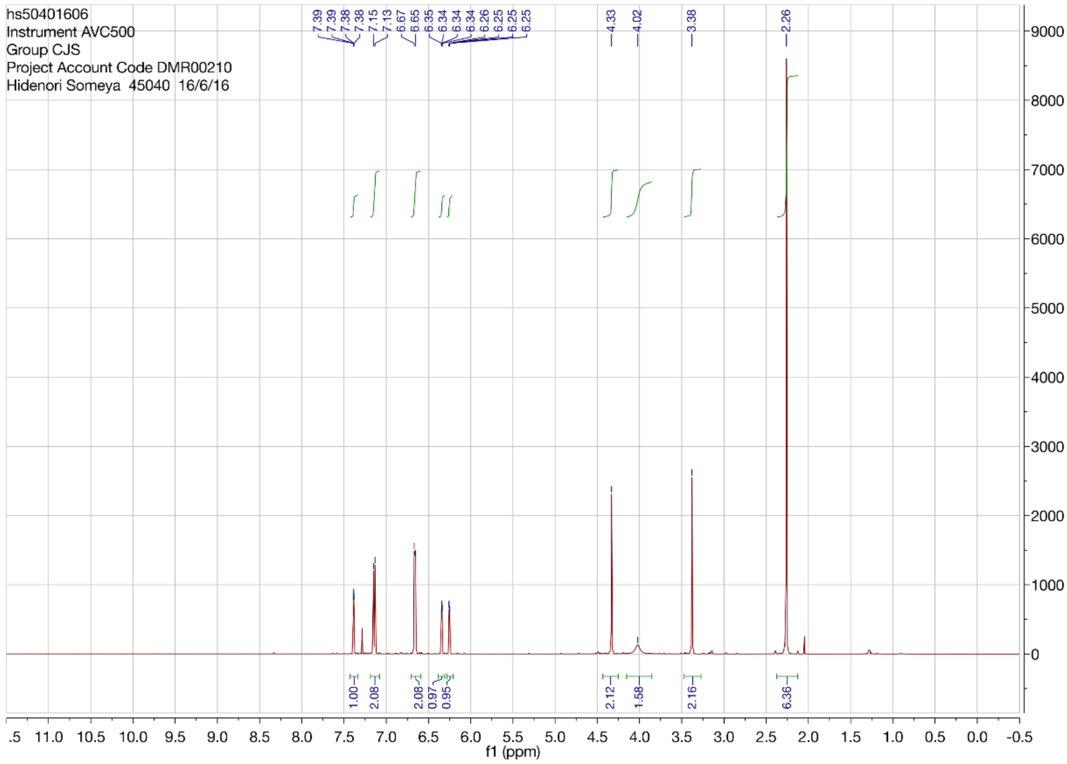

Fig. S33 ^{1}H NMR spectrum of **34a**.

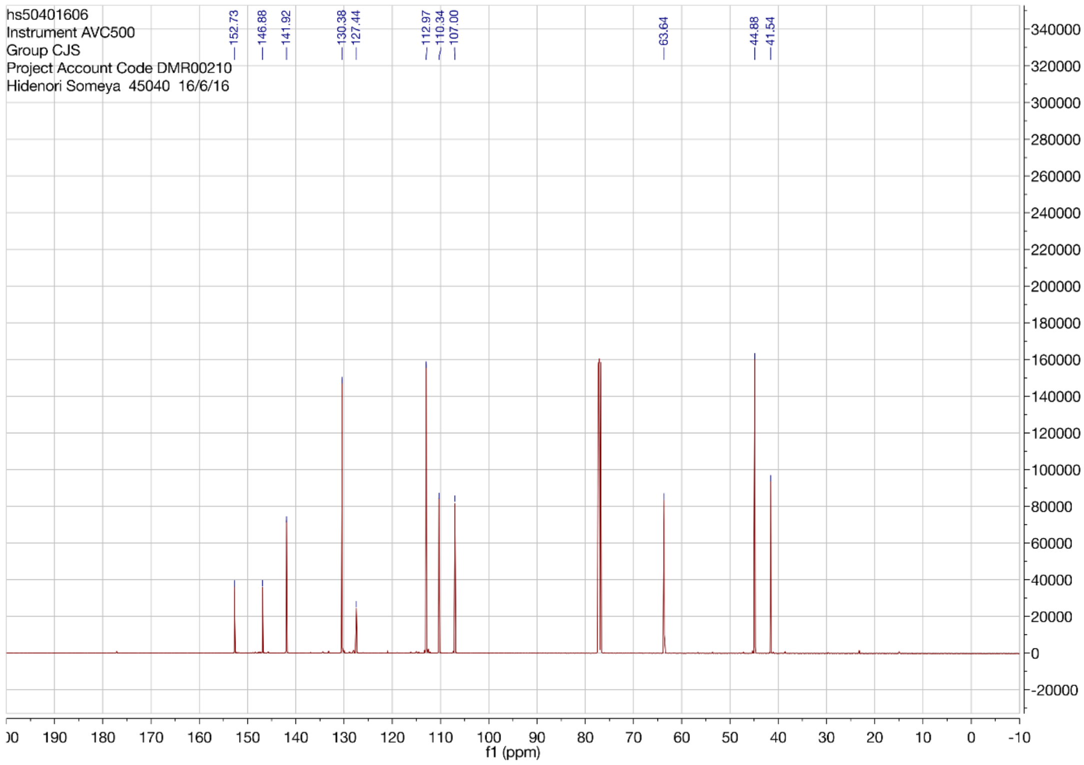

Fig. S34 ^{13}C NMR spectrum of **34a**.

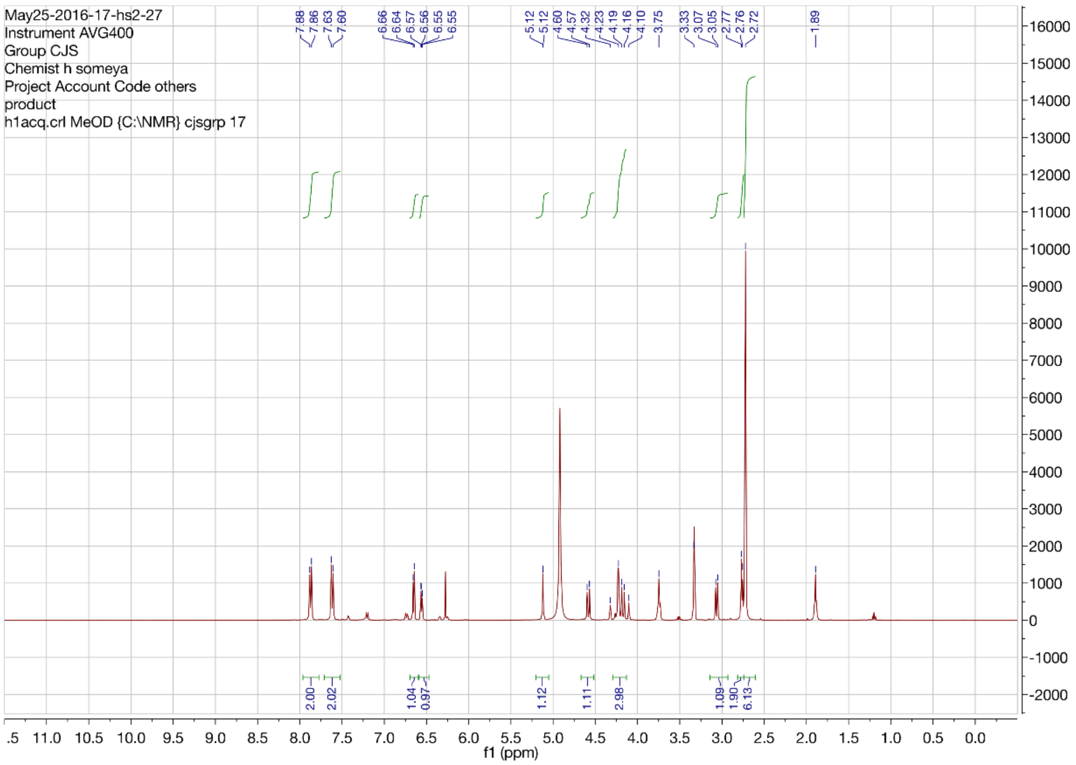

Fig. S35 ^{1}H NMR spectrum of **34b**.

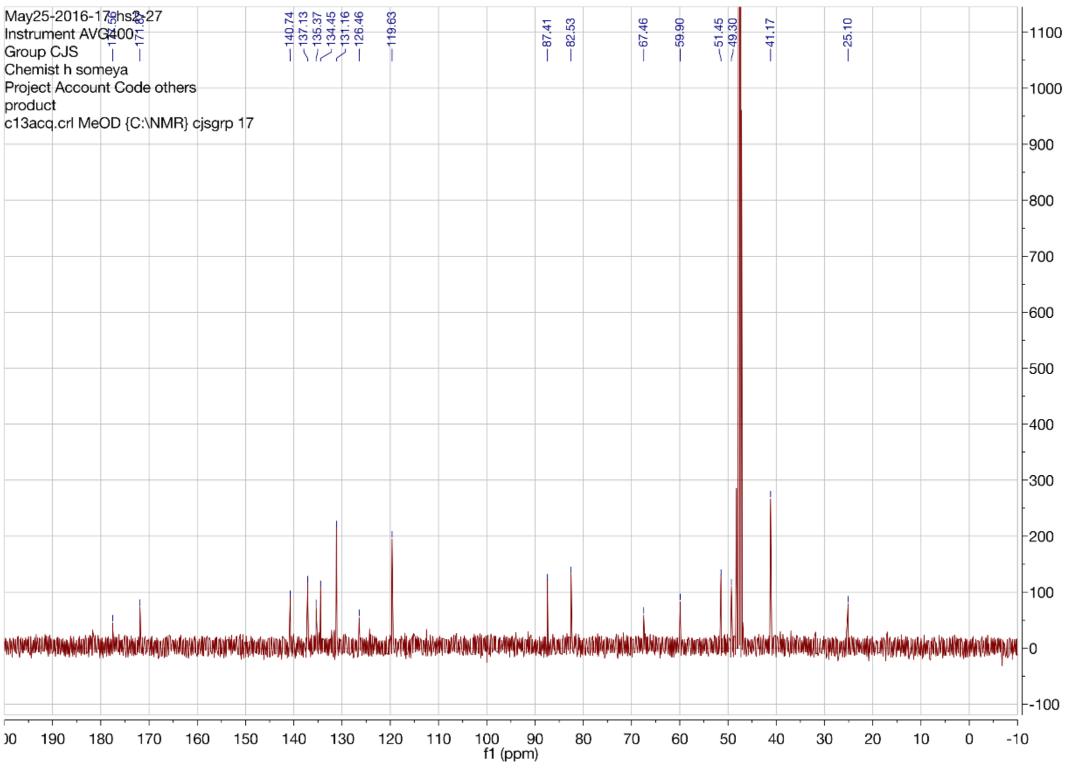

Fig. S36 ^{13}C NMR spectrum of **34b**.

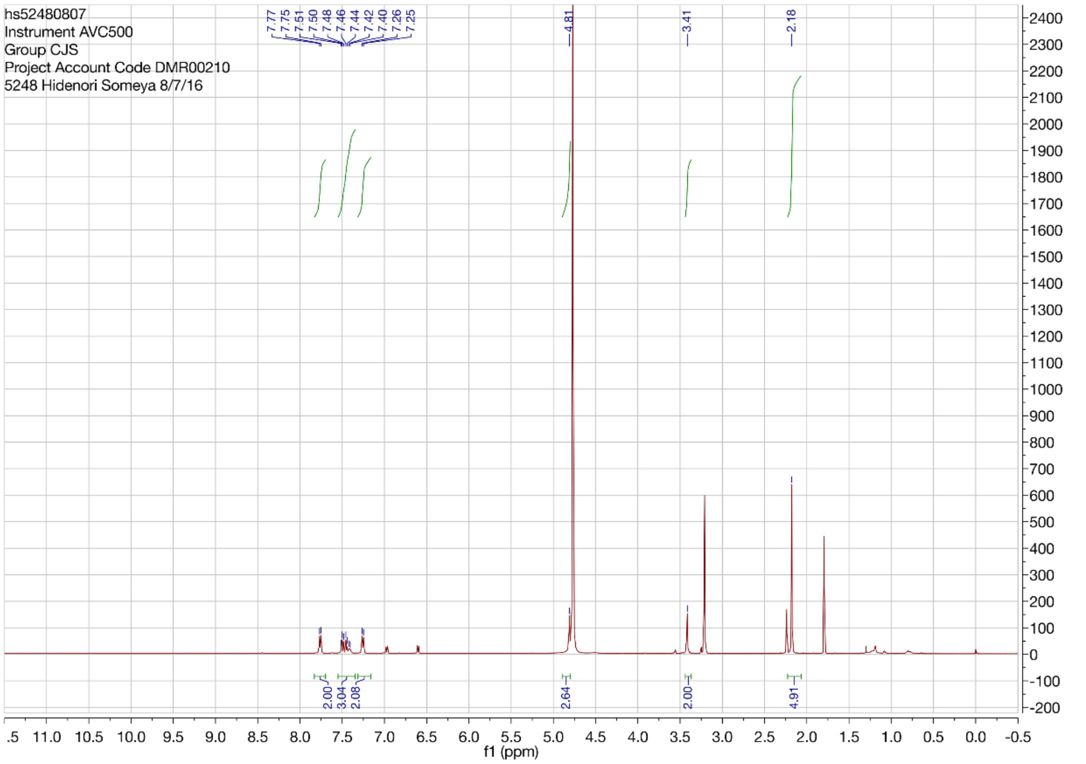

Fig. S37 ^{1}H NMR spectrum of **34**.

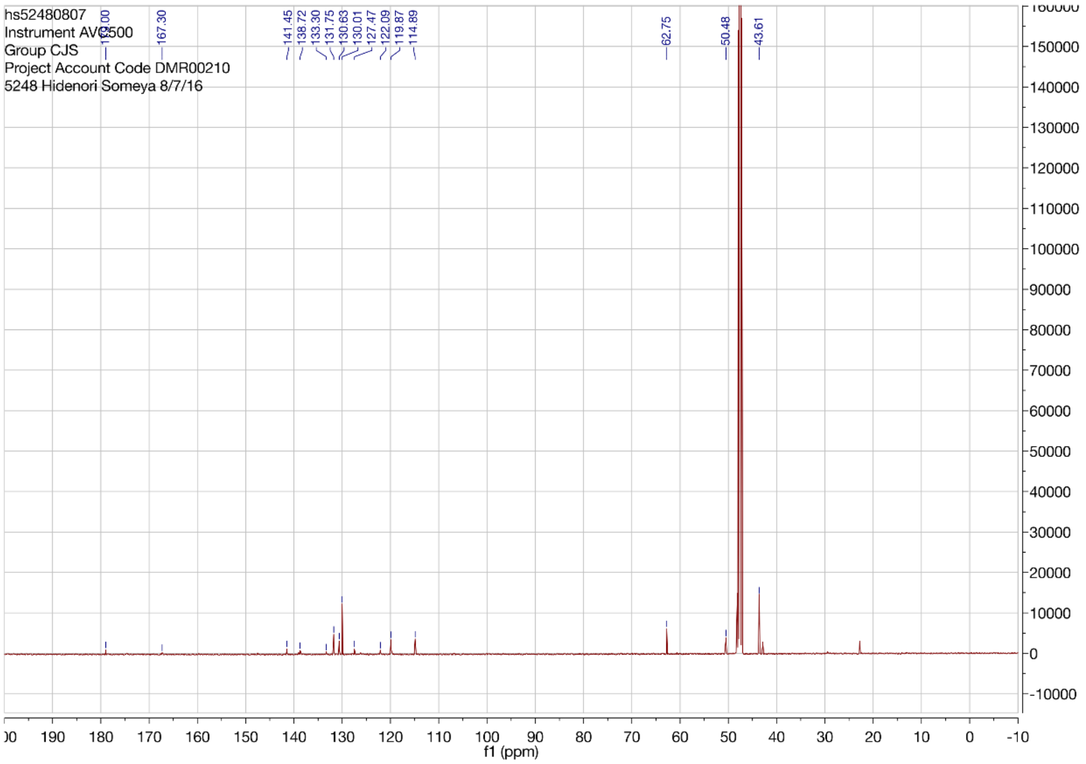

Fig. S38 ^{13}C NMR spectrum of **34**.

Supplementary Tables

Table S1. Crystallisation conditions.

Structure	Method	Protein sample composition	Crystallization reservoir condition	Experimental details
VIM-2:**16**	co-crystallization	VIM-2 in crystallization buffer[a], 1 mM TCEP, 5 mM compound **16**	0.1 M magnesium formate, 22.5% (v/v) polyethylene glycol 3350	sitting drop vapor diffusion, 1:1 protein-to-reservoir ratio, 293K
VIM-2:**17**	co-crystallization	VIM-2 in crystallization buffer[a], 1 mM TCEP, 5 mM compound **17**	0.25 M magnesium formate, 22.0% (v/v) polyethylene glycol 3350	sitting drop vapor diffusion, 1:1 protein-to-reservoir ratio, 293K
VIM-2:**32**	co-crystallization	VIM-2 in crystallization buffer[a], 1 mM TCEP, 5 mM compound **32**	0.1 M magnesium formate, 21.0% (v/v) polyethylene glycol 3350	sitting drop vapor diffusion, 1:1 protein-to-reservoir ratio, 293K
VIM-2:**35**	soaking	1 mM TCEP, 10mM compound **35**, 25% glycerol for 6 h	0.25 M magnesium formate, 21%~27% (v/v) polyethylene glycol 3350	sitting drop vapor diffusion, 1:1 protein-to-reservoir ratio, 293K
VIM-2:**39**	co-crystallization	VIM-2 in crystallization buffer[a], 1 mM TCEP, 5 mM compound **39**	0.1 M magnesium formate, 24.5% (v/v) polyethylene glycol 3350	sitting drop vapor diffusion , 1:2 protein-to-reservoir ratio, 293K

[a]Crystallization buffer = 50mM 4-(2-hydroxyethyl)-1-piperazineethanesulfonic acid (HEPES) pH 7.5, 100 mM NaCl; TECP = Tris(2-carboxyethyl)phosphine.

Table S2. Data collection and refinement statistics for PDB codes: 5LE1, 5LCA, 5LCF, 5LM6 and 5LCH.

Structure	VIM-2:**16**	VIM-2:**17**	VIM-2:**30**	VIM-2:**35**	VIM-2:**42**
PDB ID	5LE1	5LCA	5LCF	5LM6	5LCH
**Processing**					
§Radiation Source	I03	In-house (CuKα)	In-house (CuKα)	I04	In-house (CuKα)
Space Group	$P\ 2_1\ 2\ 2_1$	$P\ 2_1\ 2_1\ 2_1$	$P\ 2_1\ 2_1\ 2_1$	$C\ 1\ 2\ 1$	$P\ 1\ 2_1\ 1$
Unit Cell Dimensions *a*, *b*, *c* (Å)	45.17<br>60.72<br>97.91	40.49<br>65.31<br>70.72	40.46<br>65.64<br>70.72	102.65<br>80.07<br>68.15	39.87<br>67.84<br>40.35
Unit Cell Dimensions α, β, γ (°)	90.00<br>90.00<br>90.00	90.00<br>90.00<br>90.00	90.00<br>90.00<br>90.00	90.00<br>130.03<br>90.00	90.00<br>91.75<br>90.00
*Mol/ASU	1	1	1	2	1
Resolution Range (outer shell) (Å)	41.03-1.40 (1.43-1.40)	23.99-1.93 (2.00-1.93)	24.06-1.86 (1.91-1.86)	24.90-1.24 (1.24-1.26)	20.17-1.94 (2.0-1.94)
Number of Unique Reflections	53699	14407	16334	117930	15867
Completeness (outer shell) (%)	99.66 (96.0)	98.35 (97.0)	99.63 (98.0)	98.9(97.8)	99.33 (99.0)
$I/\sigma(I)$ (outer shell)	25.7 (2.8)	3.3 (2.4)	2.4 (1.4)	14.6(1.0)	14.6(6.7)
R_{merge} (outer shell)	9.8 (81.2)	4.7 (10.0)	6.2 (17.9)	7.3 (18.8)	5.3 (11.7)
Wilson B Factor ($Å^2$)	14.1	15.3	15.0	13.8	10.2
**Refinement**					
Overall B Factor ($Å^2$)	20.0	17.3	15.3	22.0	13.5
Protein B Factor ($Å^2$)	17.0	15.9	13.3	20.9	12.1
Ligand B Factor ($Å^2$) (occupancy)	19.8 (1.0)	31.2 (1.0)	23.8 (1.0)	34.0§§ (0.8)	19.2 (1.0)
Water B Factor ($Å^2$)	35.3	28.3	27.4	36.3	23.4
‡RMSD from Ideal Bond Length (Å)	0.014	0.009	0.010	0.009	0.013
RMSD from Ideal Angles (°)	0.948	0.647	0.771	0.970	0.816
R_{work} (%)	13.23	13.02	12.66	14.45	12.53
R_{free} (%)	15.38	16.97	16.42	16.87	16.12

§I03 and I04 are beamlines at Diamond Light Source Oxford; CuKα is in house diffractometer (CCD detector). *Mol/ASU = molecules per asymmetric unit; ‡RMSD = root mean square deviation. §§ In the VIM-2:**35** complex structure, there is clear mF_O-DF_c OMIT electron density in the active site in chain A into which **35** could be confidently modeled (Fig. S25a), the ligand density in chain B was weak and therefore not modeled.

Table S3. The assay conditions for measuring the selectivity profiles of compounds against VIM-5, VIM-1, NDM-1, SPM-1, BcII, CphA, L1, and TEM-1.

Enzyme[a]	Category[a]	[E][b], (nM)	Substrate	[Substrate], (μM)
VIM-2	Class B1 MBL	0.15	FC5	5
VIM-5	Class B1 MBL	0.15	FC5	5
VIM-1	Class B1 MBL	2	FC5	5
NDM-1	Class B1 MBL	0.5	FC5	5
SPM-1	Class B1 MBL	0.15	FC5	5
BcII	Class B1 MBL	1	FC5	5
CphA	Class B2 MBL	2.5	Meropenem	125
L1	Class B3 MBL	5	Nitrocefin	50
TEM-1	Class A SBL	2.5	FC5	5

[a] MBL: Metallo β-lactamase; SBL: serine β-lactamase; VIM, Verona Integron–encoded MBL; IMP, Imipenemase; NDM, New Delhi MBL; TEM-1: Temomeira-type 1 β-lactamase.
[b] The enzyme concentrations of the purified proteins were determined by measuring Abs@280nm using a NanoDrop (ThermoFisher) spectrometer and calculated molar extinction coefficient for each sample; concentrations of diluted solutions used in the assay were calculated from the original concentration.

Table S4. Inhibition of 4 compounds (**44-47**) that did not meet the IFP cutoff criteria as negative controls. These were tested in same assay conditions but at 2mM instead of 100uM compound concentration.

Compound	Structure	% Inhibition if VIM-2 at 2mM
44	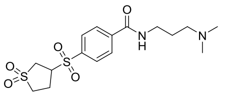	18.27% ± 6.25%
45	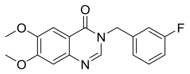	13.15% ± 6.64%
46	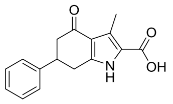	63.11% ± 3.03%
47	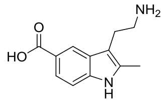	56.14% ± 5.35%

References

1. O. Trott and A. J. Olson, J. Comput. Chem., 2010, **31**, 455-461.
2. M. L. Verdonk, J. C. Cole, M. J. Hartshorn, C. W. Murray and R. D. Taylor, Proteins, 2003, **52**, 609-623.

3. J. J. Irwin, T. Sterling, M. M. Mysinger, E. S. Bolstad and R. G. Coleman, J. Chem. Inf. Model., 2012, **52**, 1757-1768.
4. S. Forli, R. Huey, M. E. Pique, M. F. Sanner, D. S. Goodsell and A. J. Olson, Nat. Protoc., 2016, **11**, 905-919.
5. T. Christopeit, T. J. O. Carlsen, R. Helland and H.-K. S. Leiros, J. Med. Chem., 2015, **58**, 8671-8682.
6. G.-B. Li, L.-L. Yang, W.-J. Wang, L.-L. Li and S.-Y. Yang, J. Chem. Inf. Model., 2013, **53**, 592-600.
7. G.-B. Li, S. Ji, L.-L. Yang, R.-J. Zhang, K. Chen, L. Zhong, S. Ma and S.-Y. Yang, Eur. J. Med. Chem, 2015, **93**, 523-538.
8. M. L. Verdonk, J. C. Cole, M. J. Hartshorn, C. W. Murray and R. D. Taylor, Proteins, 2003, **52**, 609-623.
9. G. Papeo, H. Posteri, D. Borghi, A. A. Busel, F. Caprera, E. Casale, M. Ciomei, A. Cirla, E. Corti and M. D'Anello, J. Med. Chem., 2015, **58**, 6875-6898.
10. S. S. van Berkel, J. Brem, A. M. Rydzik, R. Salimraj, R. Cain, A. Verma, R. J. Owens, C. W. Fishwick, J. Spencer and C. J. Schofield, J. Med. Chem., 2013, **56**, 6945-6953.
11. A. Makena, A. Ö. Düzgün, J. Brem, M. A. McDonough, A. M. Rydzik, M. I. Abboud, A. Saral, A. Ç. Çiçek, C. Sandalli and C. J. Schofield, Antimicrob. Agents Chemother.
, 2016, **60**, 1377-1384.
12. J. Brem, S. S. van Berkel, W. Aik, A. M. Rydzik, M. B. Avison, I. Pettinati, K.-D. Umland, A. Kawamura, J. Spencer and T. D. Claridge, Nat. Chem., 2014, **6**, 1084-1090.
13. L. Horsfall, G. Garau, B. Liénard, O. Dideberg, C. Schofield, J.-M. Frère and M. Galleni, Antimicrob. Agents Chemother.
, 2007, **51**, 2136-2142.
14. L. Nauton, R. Kahn, G. Garau, J.-F. Hernandez and O. Dideberg, J. Mol. Biol., 2008, **375**, 257-269.
15. J. A. Aguilar, M. Nilsson, G. Bodenhausen and G. A. Morris, Chem. Commun., 2012, **48**, 811-813.
16. A. J. McCoy, R. W. Grosse-Kunstleve, P. D. Adams, M. D. Winn, L. C. Storoni and R. J. Read, J. Appl. Crystallogr., 2007, **40**, 658-674.
17. P. D. Adams, P. V. Afonine, G. Bunkoczi, V. B. Chen, I. W. Davis, N. Echols, J. J. Headd, L. W. Hung, G. J. Kapral, R. W. Grosse-Kunstleve, A. J. McCoy, N. W. Moriarty, R. Oeffner, R. J. Read, D. C. Richardson, J. S. Richardson, T. C. Terwilliger and P. H. Zwart, Acta. Crystallogr. D Biol. Crystallogr., 2010, **66**, 213-221.
18. P. Emsley and K. Cowtan, Acta crystallographica. Section D, Biological crystallography, 2004, **60**, 2126-2132.
19. J. Brem, S. S. van Berkel, D. Zollman, S. Y. Lee, O. Gileadi, P. J. McHugh, T. R. Walsh, M. A. McDonough and C. J. Schofield, Antimicrob. Agents Chemother., 2016, **60**, 142-150.
20. A. I. Karsisiotis, C. F. Damblon and G. C. Roberts, Biochem. J., 2013, **456**, 397-407.
21. Y. Hiraiwa, J. Saito, T. Watanabe, M. Yamada, A. Morinaka, T. Fukushima and T. Kudo, Bioorg. Med. Chem. Lett., 2014, **24**, 4891-4894.
22. P. Hinchliffe, M. M. González, M. F. Mojica, J. M. González, V. Castillo, C. Saiz, M. Kosmopoulou, C. L. Tooke, L. I. Llarrull, G. Mahler, R. A. Bonomo, A. J. Vila and J. Spencer, Proc. Natl. Acad. Sci. USA, 2016, **113**, E3745-E3754.
23. J.-D. Docquier, M. Benvenuti, V. Calderone, M. Stoczko, N. Menciassi, G. M. Rossolini and S.

Mangani, Antimicrob. Agents Chemother.
, 2010, **54**, 4343-4351.
24. P. Lassaux, M. Hamel, M. Gulea, H. Delbrück, P. S. Mercuri, L. Horsfall, D. Dehareng, M. Kupper, J.-M. Frère and K. Hoffmann, J. Med. Chem., 2010, **53**, 4862-4876.
25. J. H. Toney, G. G. Hammond, P. M. Fitzgerald, N. Sharma, J. M. Balkovec, G. P. Rouen, S. H. Olson, M. L. Hammond, M. L. Greenlee and Y.-D. Gao, J. Biol. Chem., 2001, **276**, 31913-31918.
26. Y. Yamaguchi, W. Jin, K. Matsunaga, S. Ikemizu, Y. Yamagata, J.-i. Wachino, N. Shibata, Y. Arakawa and H. Kurosaki, J. Med. Chem., 2007, **50**, 6647-6653.
27. D. J. Payne, J. A. Hueso-Rodríguez, H. Boyd, N. O. Concha, C. A. Janson, M. Gilpin, J. H. Bateson, C. Cheever, N. L. Niconovich and S. Pearson, Antimicrob. Agents Chemother.
, 2002, **46**, 1880-1886.
28. J. H. Toney, P. M. Fitzgerald, N. Grover-Sharma, S. H. Olson, W. J. May, J. G. Sundelof, D. E. Vanderwall, K. A. Cleary, S. K. Grant and J. K. Wu, Chem. Biol. , 1998, **5**, 185-196.
29. N. O. Concha, C. A. Janson, P. Rowling, S. Pearson, C. A. Cheever, B. P. Clarke, C. Lewis, M. Galleni, J.-M. Frère and D. J. Payne, Biochemistry, 2000, **39**, 4288-4298.
30. T. Weide, S. A. Saldanha, D. Minond, T. P. Spicer, J. R. Fotsing, M. Spaargaren, J.-M. Frère, C. Bebrone, K. B. Sharpless, P. S. Hodder and V. V. Fokin, ACS Med. Chem. Lett., 2010, **1**, 150-154.
31. Y. Ishii, M. Eto, Y. Mano, K. Tateda and K. Yamaguchi, Antimicrob. Agents Chemother.
, 2010, **54**, 3625-3629.
32. P. Chen, L. B. Horton, R. L. Mikulski, L. Deng, S. Sundriyal, T. Palzkill and Y. Song, Bioorg. Med. Chem. Lett., 2012, **22**, 6229-6232.
33. W. M. Hussein, P. Vella, N. U. Islam, D. L. Ollis, G. Schenk and R. P. McGeary, Bioorg. Med. Chem. Lett., 2012, **22**, 380-386.
34. B. M. Lienard, L. E. Horsfall, M. Galleni, J.-M. Frère and C. J. Schofield, Bioorg. Med. Chem. Lett., 2007, **17**, 964-968.
35. P. Vella, W. M. Hussein, E. W. Leung, D. Clayton, D. L. Ollis, N. Mitić, G. Schenk and R. P. McGeary, Bioorg. Med. Chem. Lett., 2011, **21**, 3282-3285.
36. J. Brem, S. S. van Berkel, D. Zollman, S. Y. Lee, O. Gileadi, P. J. McHugh, T. R. Walsh, M. A. McDonough and C. J. Schofield, Antimicrob. Agents Chemother., 2015, **60**, 142-150.
37. L. Poirel, Y. Yakupogullari, A. Kizirgil, M. Dogukan and P. Nordmann, Int. J. Antimicrob. Agents, 2009, **33**, 287.
38. H. Feng, J. Ding, D. Zhu, X. Liu, X. Xu, Y. Zhang, S. Zang, D. C. Wang and W. Liu, J. Am. Chem. Soc., 2014, **136**, 14694-14697.
